# In Silico Evaluation of Some Computer-Designed Fluoroquinolone–Glutamic Acid Hybrids as Potential Topoisomerase II Inhibitors with Anti-Cancer Effect

**DOI:** 10.3390/ph17121593

**Published:** 2024-11-26

**Authors:** Octavia-Laura Oancea, Șerban Andrei Gâz, Gabriel Marc, Ioana-Andreea Lungu, Aura Rusu

**Affiliations:** 1 Organic Chemistry Department, Faculty of Pharmacy, George Emil Palade University of Medicine, Pharmacy, Science and Technology of Targu Mures, 540142 Targu Mures, Romania; andrei.gaz-florea@umfst.ro; 2 Organic Chemistry Department, Faculty of Pharmacy, “Iuliu Hațieganu” University of Medicine and Pharmacy, 41 Victor Babeș Street, 400012 Cluj-Napoca, Romania; marc.gabriel@umfcluj.ro; 3 Medicine and Pharmacy Doctoral School, George Emil Palade University of Medicine, Pharmacy, Science and Technology of Targu Mures, 540142 Targu Mures, Romania; ioana-andreea.lungu@umfst.ro; 4 Pharmaceutical and Therapeutic Chemistry Department, Faculty of Pharmacy, George Emil Palade University of Medicine, Pharmacy, Science and Technology of Targu Mures, 540142 Targu Mures, Romania; aura.rusu@umfst.ro

**Keywords:** fluoroquinolones, glutamic acid, hybrids, etoposide, anti-cancer potential, huma topoisomerase II beta-DNA complex, computational methods, docking study

## Abstract

**Background/Objectives:** Fluoroquinolones (FQs) are topoisomerase II inhibitors with antibacterial activity, repositioned recently as anti-cancer agents. Glutamic acid (GLA) is an amino acid that affects human metabolism. Since an anti-cancer mechanism of FQs is human topoisomerase II inhibition, it is expected that FQ-GLA hybrids can act similarly. **Methods:** We designed 27 hypothetical hybrids of 6 FQs and GLA through amide bonds at the 3- and 7-position groups of FQs or via ethylenediamine/ethanolamine linkers at the carboxyl group of the FQ. Hydroxamic acid derivatives were also theoretically formulated. Computational methods were used to predict their physicochemical, pharmacokinetic, or toxicological properties and their anti-cancer activity. For comparison, etoposide was used as an anti-cancer agent inhibiting topoisomerase II. Molecular docking assessed whether the hybrids could interact with the human topoisomerase II beta in the same binding site and interaction sites as etoposide. **Results:** All the hybrids acted as potential topoisomerase II inhibitors, demonstrating possible anti-cancer activity on several cancer cell lines. Among all the proposed hybrids, MF-7-GLA would be the ideal candidate as a lead compound. The hybrid OF-3-EDA-GLA and the hydroxamic acid derivatives also stood out. **Conclusions:** Both FQs and GLA have advantageous structures for obtaining hybrids with favourable properties. Improvements in the hybrids’ structure could lead to promising results.

## 1. Introduction

Recent scientific work repositioned fluoroquinolones (FQs) as potential anti-cancer agents [1]. Although their antibacterial mechanism of action is well known, several mechanisms by which they could act as anti-tumour agents are proposed. The induction of cell cycle arrest [2,3,4,5], the modulation of epithelial–mesenchymal transition [6,7,8,9], and the stimulation of cancer-specific microRNA biogenesis [1,10,11,12] are three of the possible mechanisms involved in their anti-tumour effect. Another mechanism involved in the anti-cancer effect of FQs is the induction of apoptosis.

Among the FQs, ciprofloxacin has the highest capacity to induce apoptosis. The induction of apoptosis occurs following increased levels of Bcl-2-Associated X Protein (Bax), with an imbalance between Bax and B-cell lymphoma 2 (Bcl2), mitochondrial depolarisation, and the cleavage of poly(ADP-ribose) polymerase (PARP) [2,3,10,13]. Another hypothesis of the apoptotic effect of ciprofloxacin assumes that in addition to the involvement in the mitochondrial depolarisation and dysregulation of the Bax:Bcl2 ratio, it also depends on caspases 8, 9, and 3. Ciprofloxacin has proved to be effective against several cancer cell types, such as lymphoblastoid cells, pancreatic cancer cells, colon cancer cells, and epidermoid carcinoma cells, but also colorectal, bladder, and prostate carcinoma cells [13,14,15]. Some FQs can induce apoptosis by decreasing the antioxidant glutathione levels, increasing mitochondrial dysfunction and oxidative stress, or affecting the enzyme topoisomerase II [1,16].

To achieve the purpose and objectives of this in silico study, we have further approached the following theoretical aspects in Section 1.1, Section 1.2, Section 1.3, Section 1.4 and Section 1.5: type II topoisomerase activity and the well-known inhibitors of the enzyme; the effect of FQs on this enzyme, with FQs approached as anti-cancer agents; FQ derivatives/hybrids with biological potential; the structure–activity relationship aspects among FQs, essential for repositioning them from antibacterial to anti-cancer agents; and glutamic acid’s capacity to offer derivatives with possible biological effects.

### 1.1. Type II Topoisomerase Function and Topoisomerase II Inhibitors Used in Therapy

DNA topoisomerases are enzymes that regulate the overcoiling levels of DNA during replication, transcription, recombination, and repair processes [17,18]. They work by introducing single-stranded or double-stranded breaks into DNA and restoring them. Topoisomerases are essential for maintaining DNA integrity during transcription and replication when cells proliferate because DNA supercoiling results in twisting, which affects the function of DNA or RNA polymerases [19,20,21]. Type II enzymes prevent and correct these topological problems of DNA through transient double-stranded breaks [19,20]. Type II topoisomerases perform their functions as homodimers and require divalent metal ions as cofactors, such as the Mg^2+^ ion and ATP, for catalytic activity [13,19,20].

The presence of topoisomerase in mammalian and bacterial cells and its essential role in cellular processes makes it an attractive target for antibacterial and anti-cancer drugs. Because the inhibition of DNA topoisomerases in prokaryotes results in potential antimicrobial activity, targeting their counterparts in mammals can be an effective strategy for cancer treatment [17]. The explanation lies in the fact that the inhibition of type II topoisomerase, with the impairment of its catalytic cycle, leads to apoptosis and cell death since the enzyme is considered a vital element in cell proliferation [13,20,22].

**Figure 1:**
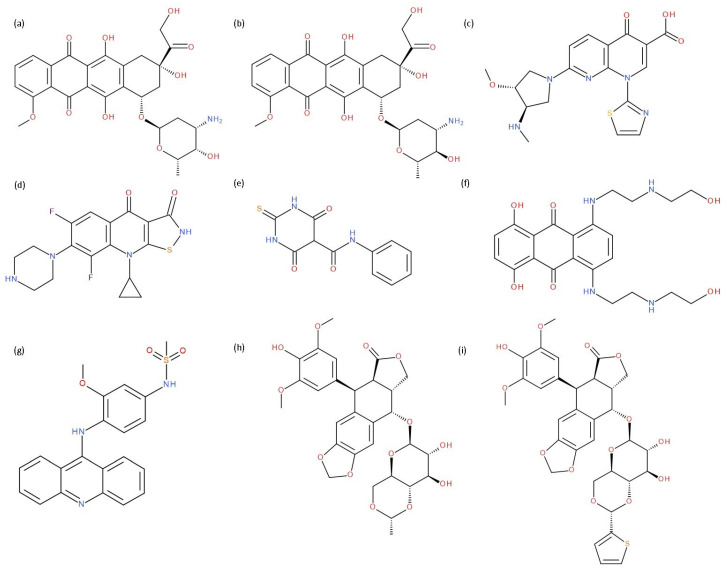
The structures of type II topoisomerase inhibitors: (**a**) doxorubicin, (**b**) epirubicin, (**c**) vosaroxin, (**d**) A-65281, (**e**) merbarone, (**f**) mitoxantrone, (**g**) amsacrine, (**h**) etoposide, (**i**) teniposide.

In cancer cells, the level of topoisomerase or its activity is enhanced to support increased cell replication and to improve cell survival. Thus, DNA–topoisomerase inhibitors could be used as an alternative therapy against cancer. Topoisomerase inhibitors target topoisomerase enzymatic activities in DNA cleavage and binding processes [19]. Doxorubicin, etoposide, teniposide, novobiocin, and FQs are compounds that “poison” type II topoisomerase [20,21]. They target the DNA–protein complex, forming irreversible covalent bonds [21]. Such compounds stop the catalytic cycle of type II topoisomerase after DNA cleavage and increase the amount of topoisomerase II-DNA cleavage complexes, resulting in genotoxic entities inside the cells. The cause of DNA damage is a deficient repair system. Consequently, accumulating these DNA breaks will eventually lead to programmed cell death [19]. Examples of topoisomerase II inhibitors and the cancer cell type on which they act are shown in Table 1. Their chemical structures are represented in Figure 1.

### 1.2. Activity of FQs on Type II Topoisomerase: FQs as Anti-Cancer Agents

The use of FQ derivatives as antiproliferative agents is of great interest to researchers, as their tumour resistance is lower than other compounds, and they have fewer chances of developing secondary tumours. Additionally, FQs present favourable pharmacological and pharmacokinetic profiles [20]. Because bacterial and eukaryotic topoisomerases are similar, FQs can inhibit DNA–topoisomerase II in mammals, even though the prokaryotic DNA gyrase is 100 times more sensitive [14,20,21]. The mechanism by which FQs induce apoptosis, manifesting its anticarcinogenic effect, appears similar to the antibacterial one [37]. It has been confirmed that stabilising the enzyme–DNA complex blocks the progression of the replication mechanism and creates DNA damage [13]. The result is the inhibition of cell division and the induction of apoptosis [17].

Among FQs, ciprofloxacin (a second-generation FQ) is distinguished by its pronounced inhibition capacity of type II topoisomerase. Moreover, it can induce the intrinsic apoptotic pathway by creating a DNA double-strand break or cell cycle arrest in the S and sub-G1 phases [17]. Ciprofloxacin has also been reported to inhibit mitochondrial type II topoisomerase, affecting cellular energy metabolism [38]. Besides ciprofloxacin [3,14,15,39,40,41,42,43,44,45], gatifloxacin [46], moxifloxacin [45], levofloxacin [47,48,49], enoxacin [48,50,51], ofloxacin [48], fleroxacin [48], and gemifloxacin [52] are other examples of FQs that are considered effective in the case of some cancerous cells. Some examples of in vitro studies in which FQs have demonstrated biological activity on different cancer cell lines and their mechanism of action are briefly presented in Table S1 from the Supplementary Materials section. Also, vosaroxin is the first quinolone derivative that belongs to a new class of antineoplastic agents [30]. Vosaroxin is one of the few drugs that targets tumours, such as acute myeloid leukaemia, that is being tested in phase III clinical trials [31,53] (Table S2—Supplementary Materials section).

### 1.3. FQ Hybrids with Different Compounds and Their Biological Effects

Quinolone derivatives are characterised by their flexibility and ease of synthesis through different procedures. Due to their structure, FQs represent a starting point for obtaining various hybrids or derivatives with other molecules to exploit their antibacterial and anti-cancer activity. Some examples of the structural derivatives of FQs or hybrids with other molecules and their anti-cancer effect are briefly mentioned below and explained in detail in Table S2 from the Supplementary Materials section. Among them are the following: hybrids of FQs with the boron atom [54]; vosaroxin [55,56]; 7-(4-(carbamoylmethyl N-substituted)) piperazine 1-yl derivatives of ciprofloxacin [57]; ciprofloxacin conjugated with fatty acids [58]; moxifloxacin/gatifloxacin/ciprofloxacin-1,2,3-triazole-isatin hybrids [59,60]; conjugates of levofloxacin with histone deacetylase inhibitors [61]; analogues of N1-decyl and C7-sec amine FQs [62]; FQ derivatives with a hydroxamic acid residue replacing the carboxyl group from position no. 3 on the quinolone nucleus [63]; the N-[4-piperazinyl]FQ-chalcone hybrid type [38]; esters formed by the condensation of (p-hydroxyphenyl)-1,2-dithiol-3-thione with an FQ [64]; C7-C7 ciprofloxacin dimers (7-(4-(alkanoyl and oxoethyl alkanoate)piperazine bond type) or C6-C6 levofloxacin dimers (C-6-(N alkylcarboxamide) bond type) [65]; moxifloxacin–copper complexes [66]; N-piperazinylquinolone derivatives [67]; N-[2 substituted-2-(2-thienyl)ethyl]piperazinyl quinolone derivatives [68]; and N-(2-oxyimino ethyl)piperazinyl quinolone compounds [69].

### 1.4. Structure–Activity Relationship Among FQs

**Figure 2:**
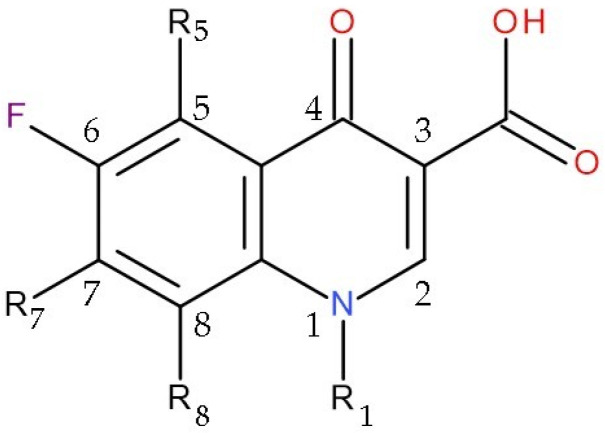
The basic core of the FQs structure.

FQs do not participate in direct associations with free nucleic acids. Their mode of action implies binding to the enzyme–DNA complex, with the formation of the new complex, enzyme–FQ-DNA. Due to their structure (Figure 2), it is considered that FQs interact with topoisomerase II through position 7 of the nucleus [67]. The quinolone activity seems directed towards prokaryotic or eukaryotic enzymes, depending on the substituent in the C7 position [13].

Among the mandatory structural features of FQs regarding the anti-cancer effect are acidic groups (carboxyl group in the C3 position) and a nitro or amino group at the C8 position [30]. The nitrogen atom in position 1 is essential for anti-cancer activity, and bulky groups, such as butyl residue, are favourable [30,70]. Also, increasing the number of hydrogen bonds involved in the molecule has a beneficial effect [30,37]. The carboxyl moiety in the C3 position and the carbonyl group in the C4 position contribute to forming hydrogen bonds with nitrogenous bases from the DNA structure, essential for the anti-tumour effect [17,65]. On the contrary, the lipophilic nature of FQs contributes to their excellent penetration capacity into the cancer cell through the lipid bilayer. The anti-tumour efficacy of FQs can be improved by increasing the lipophilicity of the new compounds [30,37]. Thus, firstly, decreasing the zwitterionic effect of the FQ molecule influences its lipophilicity and represents a way of developing new anti-tumour analogues of FQs with increased selectivity for human topoisomerase II [13,17,30,37,70]. Secondly, the interaction of FQs with single-stranded DNA can be redirected towards the double chain by adding aromatic and condensed cycles to its structure, especially in position 7 [13,17].

The inhibition of human topoisomerase II requires collinearity of the substituent from position 3 with the quinoline nucleus [13,70]. Also, the substitution in the 5-position can affect the steric configuration and planarity of the entire compound [17]. In addition, quinolones with two or more fluorine groups, such as sparfloxacin (two moieties) or trovafloxacin (three moieties), have been shown to have anti-tumour activity in the P388 murine leukaemia model [71]. Thus, the cytotoxicity of FQs increases when the C8 position is a halogen substituent [67,68].

These are some structural features to look out for when redirecting the activity of FQs to the anti-cancer one. However, the structure offers many possibilities to create structural derivatives or hybrids with other molecules, an advantageous aspect in repositioning FQs from antibacterial to anti-cancer agents. Table S3 from Supplementary Materials presents more details regarding the structure–activity relationship of FQs.

### 1.5. Design of Glutamic Acid Hybrids with FQs

Glutamic acid (GLA) is an essential amino acid with multiple implications in human metabolism. Over time, both the structure of GLA and FQs have been studied to improve some of their physicochemical or biological properties. Structural derivatives or hybrids with other molecules were designed. They were subsequently tested on cell lines, in vivo, on experimental animals, and in clinical trials, or even used as therapeutic alternatives in certain types of cancer. Some structural derivatives of GLA and their utility in the oncological sphere are presented in the paper of Moldovan O.-L. et al. (2021) [72]. Also, in the article of Moldovan O.-L. et al. (2023) [73], a compound library with possible biological potential (cytotoxicity), starting from the structure of GLA, was created. The physicochemical and biological properties were predicted using computational methods. A molecular docking study was conducted for the compound with the best-predicted properties to evaluate the interaction of the ligand with a known target. We concluded that GLA has an advantageous structure, and GLA-derived molecules could become valuable drugs.

Because of the promising results obtained in this study [73] and the FQs’ basic structure, which offers many possibilities to create structural derivatives or hybrids with other molecules (with the repositioning of FQs from antibacterial to anti-cancer agents), we concluded that GLA and FQs are compounds that could be easily manipulated, and molecules derived from both their structures have great potential to become innovative drugs. So, considering all of the above-mentioned information, we hypothesise that some of the hybrids formed between several common FQs and GLA will also bind to human type II topoisomerase. If this is confirmed, there might be a possibility of a beneficial effect occurring on certain cancer cell lines, as in the case of other topoisomerase II inhibitors with anti-cancer effects. Positive results could lead to new research directions that can contribute to new possible therapeutic alternatives in the oncological sphere.

Therefore, this paper uses computational methods to characterise the theoretically formulated hybrids by predicting their physicochemical properties, biological behaviour, anti-cancer activity, and toxicological profile. The purpose was to compare the properties of our hybrids with those of a well-known topoisomerase II inhibitor, etoposide. Furthermore, to complete our study, we assessed the interaction of our hybrids with the human topoisomerase II beta using a molecular docking tool. We evaluated if they could interact with the type II topoisomerase in the same binding site and interaction sites as etoposide.

## 2. Results

### 2.1. Evaluation of the Tanimoto Coefficient Using Etoposide as a Reference Compound. Prediction of Physicochemical, Pharmacokinetic, and Structural Properties, Biological Behaviour, Anti-Cancer Activity, and Toxicological Profile

The ID, chemical structure, molecular weight, and Tanimoto coefficient of the hybrids, evaluated using etoposide as a reference compound, are listed in Table 2. Table 3 presents some structural and physicochemical properties predicted by SwissADME (http://swissadme.ch, accessed on 25 May 2024) (the McGowen volume and geometric span were predicted with the FORECASTER platform, v 6453). In Table 4, we characterise the compounds regarding the lipophilicity profile and the partition coefficients, which were evaluated with SwissADME and MarvinSketch, v 23.11. The permeability (blood–brain barrier permeation (BBB) and skin permeation (log Kp)), the gastrointestinal absorption, and the interactions with P-gp predicted with SwissADME are shown in Table 5. We also listed the Central Nervous System Multiparameter Optimization (CNS MPO) score here, which was evaluated with MarvinSketch. In Table 6, we present the number of broken rules, according to Lipinski, Ghose, Veber, Egan, and Muegge, the bioavailability score, the lead-likeness score, the synthetic accessibility score (SwissADME), and the drug-likeness score (Osiris Property Explorer, https://www.organic-chemistry.org/prog/peo/, accessed on 25 May 2024).

The enzyme inhibitory effect of the hybrids and etoposide on several isoforms of cytochrome P450 (CYP1A2, CYP2C19, CYP2C9, CYP2D6, CYP3A4) was also assessed using the SwissADME platform. None of the hybrids showed inhibitory activity against these isoforms. Etoposide presented inhibitory activity only on the CYP2D6 isoform.

The bioactivity was assessed with the platform Molinspiration, v 2022.08 (the activity on G-protein-coupled receptors (GPCR) and nuclear receptors, the ion channel modulator capacity, and the kinase, protease, and enzyme inhibitory capacity) and it is detailed in Table 7. The topoisomerase II inhibitory activity of the hybrids and etoposide assessed with PASS Online, v 2.0 is also presented here. The number of hybrids that have anti-cancer effects on different cancer cell lines is shown in Table 8 (CLC-Pred, v 2.0). Table 9 also shows the most probable toxic/adverse effects and the number of hybrids that could produce them (PASS Online).

Also, valuable results are presented in the Supplementary Materials section, Tables S4–S15:

- Table S4 presents the SMILES string, IUPAC name, InChI string, and InChI key (Biovia Draw, v 21.1), and Table S5 offers information regarding the water solubility of the compounds (according to Aquasol, v 2.0 and SwissADME platforms). Table S6 presents the pKa score, isoelectric point, molar polarizability, and best conformation for each hybrid (E min) assessed with MarvinSketch;
- Tables S7 and S8 complete the information regarding the bioactivity: the mechanisms of action and the adverse/toxic effects were assessed with the PASS Online platform (Table S7), and the most probable cancer cell lines for which compounds exhibited cytotoxicity (CLC-Pred platform) (Table S8). Also, the programme Toxtree, v 3.1.0.1851 and the platform SmartCyp, v 3.0 were used to characterise the metabolic behaviour of the compounds (Tables S9 and S10);
- The characterisation of the compounds regarding the multiple toxicity aspects described in Table 9 was completed with the information from Tables S11–S15, as follows: the acute toxicity in rodents when administered intraperitoneally, intravenously, orally, and subcutaneously—LD50 in mg/kg (Table S11); the acute toxicity in rodents—the classification of chemicals by the OECD Project (Table S12); the toxicity according to Cramer’s rule, Kroes TTC, and the Verhaar scheme, assessed with the Toxtree programme (Table S13); the carcinogenic (genotoxic and non-genotoxic) and mutagenic effects evaluated using Toxtree and Osiris Property Explorer (Table S14); the irritant/corrosive effect on the skin and eyes; the effect on the reproductive system; the biodegradability; and the protein and DNA binding alerts, assessed using Toxtree and Osiris Property Explorer (Table S15).

### 2.2. Self-Docking Results

**Figure 3:**
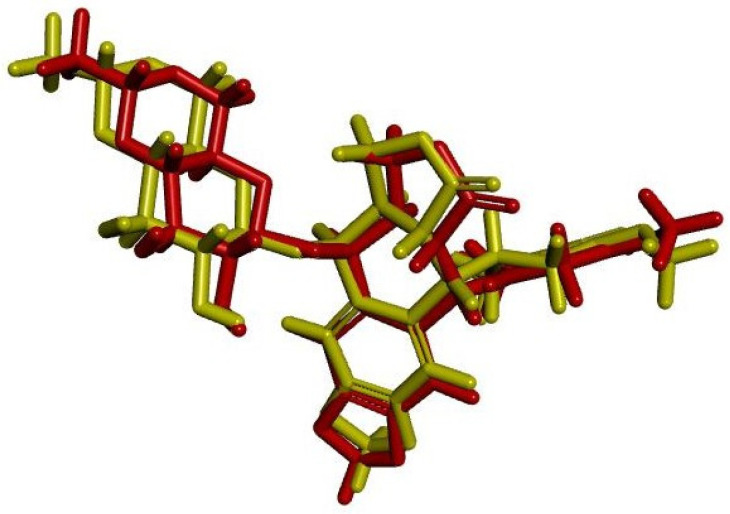
Comparison of the docked pose (yellow) and the original ligand (red) structures.

**Figure 4:**
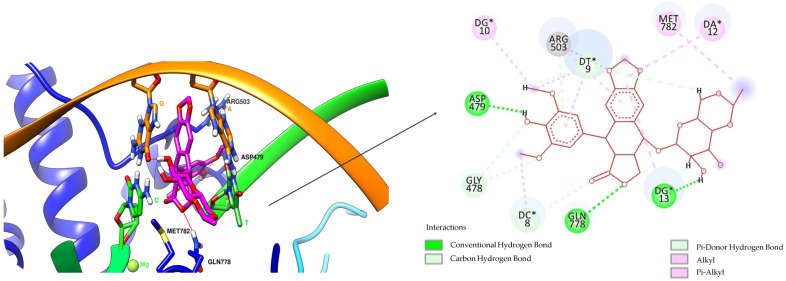
The interactions of etoposide with the residues of the enzyme–DNA complex binding site—3D (**left**) and 2D (**right**) structures.

The self-docking step was performed with the FORECASTER platform to validate the method intended for assessing the hybrids’ interaction with the target enzyme. This step involved the docking of etoposide to the human topoisomerase II beta—DNA complex (the human topoisomerase II beta complexed with DNA and etoposide provided by the Protein Data Bank (PDB, https://www.rcsb.org/, accessed on 27 June 2024)—with the PDB complex code of 3QX3—was used) and the results were as follows: an internal energy (kcal/mol) of −95.7; a root mean square deviation (RMSD) of 0.56 Å; a Rank Score of −32.3; a Match Score of 50.1; and a Fitted Score of −40.3 (the values obtained for the best docking score after three runs). The binding site amino acid residues in the case of the self-docking and docking procedure with the FORECASTER platform were the following: LYS456, THR476, GLU477, GLY478, ASP479, SER480, ALA481, LYS482, PRO501, LEU502, ARG503, GLY504, LYS505, ALA521, GLU522, THR556, ASP557, ILE565, GLY776, GLU777, GLN778, ALA779, MET781, MET782, VAL785, ALA816, ALA817, SER818, PRO819, ARG820, ARG820, and TYR821. Figure 3 presents the docked pose and the original ligand (crystal ligand), and Figure 4 shows the interactions of the ligand with the residues of the enzyme–DNA complex binding site as 3D (left) and 2D (right) structures.

### 2.3. Docking Results

The docking step was conducted for all 27 hybrids using the topoisomerase-DNA complex’s binding site and interaction sites obtained with the FORECASTER platform in the self-docking phase for etoposide. The docking results (after three runs) for the hybrids (the internal energy, Rank Score, Match Score, and Fitted Score) are presented in Table 10.

Also, molecular docking was performed for all 27 hybrids with the software AutoDock Vina, v 1.1.2 and AutoDock, v 4.2 and the results are presented in Table 11.

**Figure 5:**
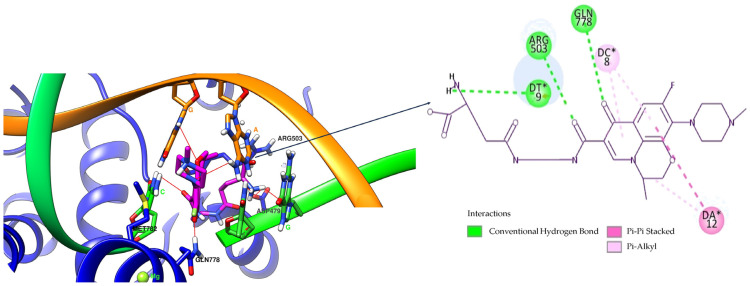
The interactions of the ligand (the hybrid OF-3-EDA-GLA) with the residues of the enzyme–DNA complex binding site—3D (**left**) and 2D (**right**) structures.

**Figure 6:**
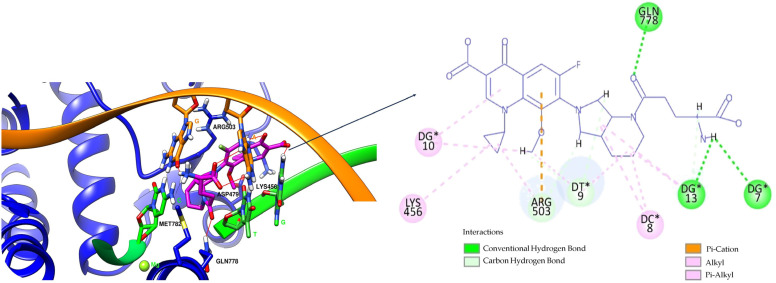
The interactions of the ligand (the hybrid MF-7-GLA) with the residues of the enzyme–DNA complex binding site—3D (**left**) and 2D (**right**) structures.

**Figure 7:**
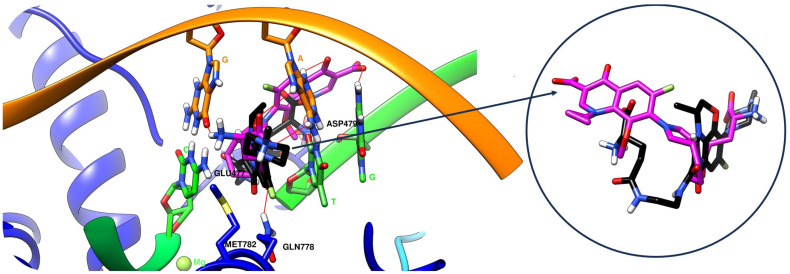
The overlay of binding poses of the hybrids OF-3-EDA-GLA (black) and MF-7-GLA (purple).

Figures S2–S6 illustrate the conformations of the ligands in the binding site and their interaction with the enzyme–DNA complex. Figure 5 and Figure 6 present the interactions (3D to the left and 2D to the right) of the hybrids with good binding affinity for the binding site of the enzyme–DNA complex obtained with all three software: the hybrids OF-3-EDA-GLA (Figure 5) and MF-7-GLA (Figure 6). Figure 7 presents the overlay of the binding poses of the hybrids OF-3-EDA-GLA (black) and MF-7-GLA (purple).

## 3. Discussion

The classes of hybrids were selected considering new structural variants with few changes in the molecules of the primary compounds. We focused on not negatively influencing the properties of the FQs but also proposing compounds that could be synthesised through accessible chemical reactions. Our design strategy preserved the two C=O bonds in the C4 position (as ketone) and C3 position (as carboxyl or amide) of the FQ’s ring, which is a common structural motif of the potent topoisomerase II inhibitors, such as merbarone, vosaroxin, and A-6528 (Figure 1). This aspect is essential for coordinating with the Mg^2+^ ion, which is critical in promoting the DNA cleavage-joining activity of the type II topoisomerase. This strategy was applied to all the formulated hybrids [20].

The positions C3 and C7 of the FQs ring were our targets, and introducing the GLA molecule increased the number of hydrogen bonds that would be beneficial for biological activity. Due to the carboxyl group in the C3 position, an amide bond with the amino group of GLA was possible. Various ways of binding GLA to the carboxyl group were created using linkers of at least two carbon atoms (ethanolamine/ethylenediamine). In these hybrids, binding was achieved through amide (ethylenediamine) or ester and amide (ethanolamine) bonds.

The hybrids formed by modifying the C7 position of the FQs ring were formed by amide bonds between the carboxyl group of GLA (gamma position) and the nitrogen atom of the FQs heterocycle grafted at the C7 position. The aim was to exploit the possibility of forming amide bonds between GLA and FQs.

In this study, we have chosen the hydroxamic acid class, which is considered to have intense anti-cancer activity by inhibiting histone deacetylase [84]. Thus, these hybrids showed potential for our study.

As far as we studied the scientific literature, no such hybrids have been designed or synthesised. We characterised the hybrids, trying to outline a complex profile by approaching multiple perspectives and using several programmes and platforms to obtain all the predicted properties. We compared the proposed hybrids with etoposide, as it is a well-known topoisomerase II inhibitor used in anti-cancer therapy, for which PDB provided a high-resolution crystalline structure in complex with the protein and the DNA. We hypothesised that our hybrids would link to the enzyme in the same binding site as the etoposide, considering that etoposide and FQs are topoisomerase II inhibitors.

Etoposide is a semisynthetic derivative of podophyllotoxin included as part of initial chemotherapy for many cancer types, including small-cell lung cancer, testicular cancer, or Kaposi’s sarcoma [85,86]. Etoposide’s effect is completed with other drugs, administered in combination, for treating different types of cancer, such as lung cancer, lymphoma, or leukaemia, mainly associated with cisplatin, carboplatin, and cyclophosphamide [87].

The inhibition of type II topoisomerase is considered a primary anti-cancer mechanism of etoposide. Primarily, etoposide operates during the late S and G2 phases of the cell cycle [85]. This interplay between the protein, the DNA, and the etoposide explains its structure–activity relationship. Chyuan-Chuan W. et al. (2011) [88] analysed the crystal structure of the etoposide-stabilised cleavage complex. The bound etoposide interacted extensively with protein and DNA; the interaction is detailed in the author’s paper. All the DNA base pairs, two etoposide molecules, and six Mg^2+^ ions were visible in the electron density maps—the active centre of the enzyme was assembled in trans with the catalytic tyrosine (WHD domain), and the Mg^2+^ was chelating three acidic amino acid residues (TOPRIM domain). The etoposide partially stabilised the cleavage complex by disfavouring the relegation of cleaved DNA ends by decoupling key catalytic residues. The interactions between the etoposide and the surrounding amino acid residues outlined the topoisomerase’s II central role in stabilising the interaction between the etoposide and DNA, indicating that the drug displayed a low affinity for free DNA [88].

### 3.1. Tanimoto Coefficient. Physicochemical Properties

Virtual screening of databases is a popular method for preselecting available compounds for physical screening. Similarity searching is a relatively simple technique based on the assumption that increasing chemical similarity correlates with an increasing chance that two compounds share the same activity (the similarity–property principle) [89,90]. Quantifying the similarity between molecules implies extracting structural features as subgraphs from the graph of molecular structures. The set of extracted structural features will characterise the molecule. This set of features is often referred to as a topological fingerprint, and the degree of similarity between the two molecules increases with the number of features they have in common [91]. As defined by the fingerprints, the similarity of these molecules can be assessed using a coefficient [92].

The similarity coefficient most frequently combined with the use of fingerprints is the Tanimoto coefficient (Tc), also known as the Jaccard coefficient [91,92,93,94,95].

Many different types of molecular fingerprints are used to calculate the similarity between two molecules. Two of the most widely used are the molecular access system (MACCS) fingerprint and the extended connectivity fingerprint (ECFP) [92]. In our study, we calculated the Tc using the MACCS keys, a classical fingerprint in cheminformatics consisting of a dictionary of 166 structural fragments [90,92].

The Tc has a range from 0 to 1, corresponding to no fingerprint overlap (lowest similarity) and fingerprint identity (highest similarity) [90,91]. Regarding a significance threshold of the Tanimoto similarity coefficient, a value of 0.85 for MACCS was widely considered relevant for bioactivity [92]. Thus, a Tc > 0.85 indicates that two molecules have similar activities. However, a Tanimoto similarity of 0.8 [90] or even just 0.7 is elsewhere considered as a cut-off for read-across, with a 70% overlap in features suggesting a substantial molecular resemblance [92]. In our study, when comparing the hybrids to etoposide, seven hybrids reached the Tc threshold of at least 0.7, MF-3-GLA, MF-7-GLA, NF-3-GLA-HA, OF-3-GLA-HA, LF-3-GLA-HA, PF-3-GLA-HA and MF-3-GLA-HA, the last hybrid presenting the highest value, 0.72. Interestingly, five of the seven hybrids were hydroxamic acid derivatives (out of six FQ-3-GLA-HA designed hybrids), and the other two were derived from moxifloxacin. The additional -OH group may explain the high Tc for the hydroxamic acid derivatives compared to the other hybrids. The etoposide molecule has three hydroxyl groups. These Tc values may indicate that, based on structural similarity, the hybrids could exhibit similar activity to etoposide or present similar activity trends. However, when choosing the hybrid with the best properties, we considered the Tc, but we correlated it with the molecular docking results and the predictions for topoisomerase II inhibitory activity.

We started to characterise the hybrids and etoposide by assessing their structural features and physicochemical properties, such as the heavy atoms (HA), the heavy aromatic atoms (HAA), the Csp3 fraction, the H-bond acceptors, the H-bond donors, the rotatable bonds (RB), the molar refractivity (MR), the topological polar surface area (TPSA), the McGowan volume, and the geometric span. We tried to observe if a class of hybrids or a specific hybrid had a closer structural and physicochemical profile to etoposide. Although choosing one specific hybrid that matched most of these properties with etoposide was difficult, we tried to evaluate which one seemed to be the most similar from this perspective. Considering the molecular weight (MW) of the compounds, the ones that were the closest to etoposide (MW 588.6 g/mol) were the hybrids D6 and E6 (MF-3-EA-GLA and MF-3-EDA-GLA). Regarding the HA and moxifloxacin derivatives, such as MF-3-EA-GLA and MF-3-EDA-GLA, presented the closest values (41) to etoposide (42). Also, the HAA number for all the hybrids was 10, which was very close to the etoposide (12). The predicted Csp3 fraction indicates that the moxifloxacin derivatives were closer to etoposide: MF-3-GLA, MF-7-GLA, MF-3-GLA-HA, MF-3-EA-GLA, and MF-3-EDA-GLA.

Regarding the RB, according to SwissADME, variations between etoposide (5 RB) and the hybrids were observed (the hybrids presented at least 8 RB). The highest value for the H-bond acceptors was for etoposide (13), while the hybrids presented a maximum of 10 in the case of the FQ-3-EA-GLA hybrids. The number of H-bond donors for the hybrids was very close to the one for etoposide.

MR measures the polarisability of a molecule [96]. In our study, several hybrids from different classes presented close values to etoposide: PF-3-EDA-GLA was the closest one, followed by PF-EA-GLA and CF-3-EDA-GLA. Also, the MF derivatives (MF-3-GLA and MF-7-GLA) and OF-3-EA-GLA and LF-3-EA-GLA can be mentioned here.

TPSA increases with the number of polar groups in a structure, an indicator of liposolubility and biological membrane penetration capacity [97]. A TPSA value under 140 Å^2^ indicates good intestinal absorption, with values lower than 60 Å^2^ corresponding to good BBB penetration. TPSA is a good predictor for cellular absorption, with the ideal range for absorption being between 60 and 140 Å^2^. In our study, all the values, including etoposide, were above 140 Å^2^, except PF-3-GLA (132.18 Å^2^). These results suggest that the compounds were characterised by low lipophilicity, an idea confirmed by the other parameters predicted and detailed below. Some of the hybrids had values closer to the ones of etoposide (160.83 Å^2^): the compounds from the FQ-3-EA-GLA, FQ-3-EDA-GLA classes, and the MF-7-GLA and MF-3-GLA-HA hybrids.

The McGowen volume, expressed as ml/mol, is considered to be the actual volume of a mole when molecules are not in motion. It is proportional to the parachor—the molecular volume at temperatures where surface tensions are equal [98]. The compounds with the closest McGowen volume to etoposide were MF-3-GLA-HA, OF-3-EDA-GLA, and LF-3-EDA-GLA.

The geometric span, considered a size descriptor, is defined as the radius of the smallest sphere, centred on the centre of mass, completely enclosing all molecule atoms. The geometric span of the etoposide was closer to that of the CF-3-GLA and NF-3-GLA hybrids, followed by the MF-7-GLA and ciprofloxacin and norfloxacin derivatives from the FQ-3-GLA-HA class.

### 3.2. Pharmacokinetic Properties, Drug-Likeness, and Lead-Likeness

We also used SwissADME to predict pharmacokinetic properties such as gastrointestinal absorption, the blood–brain barrier and skin permeability, and the interaction with P-gp. According to the predictions, most hybrids have low gastrointestinal absorption (except the compounds from class FQ-3-GLA, without MF-3-GLA). A higher molecular weight could be one of the impediments to good gastrointestinal absorption. Also, the HLB values, the logP score, and the logD score of the hybrids indicate that the compounds present high hydrophilicity. The high HLB values from MarvinSketch indicate the hybrids’ hydrophilic nature. Also, the parameters logP and logD are descriptors for lipophilicity, helping to predict the in vivo permeability of the active compounds in drug discovery. The negative values for logD indicate that this compound would be more susceptible to higher aqueous solubility and lower lipophilicity, expecting poor membrane permeability [99], which appeared to be our case when predicting it with MarvinSketch.

Regarding logP, it is believed that a value between 0 and 2 should be optimal, and water-soluble drugs tend to have negative logP values. Some sources also consider that ranges between 4 and 5 appear in control release drugs, while values between 1 and 3 should present favourable pharmacokinetic results [100]. We conducted logP evaluation through multiple methods, described in the legend of Table 4 (SwissADME), and we also predicted the logP score using MarvinSketch. We consider that the logP results also indicate that, in general, the hybrids tend to be hydrophilic. Therefore, we hypothesise that, based on the abovementioned information, the hybrids tend to have low lipophilicity, influencing gastrointestinal absorption.

Weak lipophilicity was also confirmed by the results obtained after evaluating the hybrids’ BBB penetration capacity, observing that none could cross it. Furthermore, the CNS MPO score supports this result. Central Nervous System (CNS) drugs are the ones that have to penetrate the BBB to have any effect on their target [101]. The CNS MPO score is between 0 and 6.0, with values ≥ 4.0 (desirability score) widely used as the cut-off to select candidates for hit finding in CNS therapeutic area drug discovery studies [102]. In our case, neither the hybrids nor etoposide equalled or exceeded the limit score of 4. Since we did not focus on passing the BBB and manifesting any effects at the CNS, this did not represent a problem. Also, we consider this to be a favourable aspect of safety. Additionally, with a few minor exceptions, most of them were substrates for P-gp.

Skin permeation was also predicted for all the hybrids to outline a more complex pharmacokinetic profile, and negative values were obtained. It is considered that the lower the log Kp (with Kp in cm/s), the less permeant the molecule is to the skin [103,104]. Generally, a low skin permeation is confirmed when a molecule’s log Kp exceeds −2.5 cm/s [105]—all the hybrids and etoposide presented lower values.

Besides absorption and distribution, we also evaluated the metabolic pathways in which they participated (Table S9—Supplementary Materials), and we identified the most likely site of metabolism (the most reactive atom involved in the interactions with CYP3A4, CYP2D6, and CYP2C9). A lower score corresponded to an increased probability of being a site of metabolism (Table S10—Supplementary Materials) [106]. Also, because Cytochromes P450 are the most crucial class of drug-metabolising enzyme [107], we wanted to observe if any of the hybrids could inhibit the CYP enzymes (CYP1A2, CYP2C19, CYP2C9, CYP3A4, CYP2D6). The results were negative for all the hybrids and all the CYP isoforms. These predictions also suggest that etoposide was the only compound inhibiting the CYP2D6 isoform.

Drug-likeness refers to the resemblance of a new compound to existing drugs and assesses, qualitatively, the chance for a molecule to become an oral drug concerning bioavailability [108]. In our study, besides Lipinski’s rule, we evaluated if the compounds corresponded to other rules, such as the ones of Ghose, Veber, Eagan, and Muegge. The characteristics of each rule, according to SwissADME, are presented in Figure S1 in the Supplementary Materials section. Most of the hybrids broke Lipinski’s rule, except NF-3-GLA, PF-3-GLA, CF-3-GLA, NF-7-GLA, and CF-7-GLA. It also predicted a high gastrointestinal absorption for the first three compounds mentioned here, following the abovementioned information regarding the pharmacokinetic profile. Also, most of the hybrids violated at least one characteristic of each of the other drug-likeness rules, with a few exceptions (Table 6). However, we must consider that over time, the studies came up with a new approach—to analyse this concept of drug-likeness beyond such rules, as a rigid interpretation of Lipinski’s rules comes at the expense of chemical diversity. This aspect can be observed even in the case of successful drugs such as atorvastatin, montelukast, cefixime, macrolides, rifampicin, tacrolimus, cyclosporine A, itraconazole, and digoxin, which are approved and used in therapeutic practice as well as in compounds that are in clinical studies [109,110]. We also mention the well-known antiviral agents, such as HIV protease inhibitors, and the new approvals of direct-acting antivirals, including hepatitis C (HCV) NS5A, and NS3/4A protease inhibitors, as well as oncology drugs, such as Bcl-2 inhibitors [111].

Regarding the drug-likeness score provided by Osiris Property Explorer, it is considered that a positive drug-likeness value indicates that the molecule contains predominantly fragments, which are frequently prevalent in commercial drugs [112]. However, it does not necessarily mean these fragments are well-balanced in terms of other properties. A molecule may be composed of a drug-like but lipophilic fragment only. This molecule will have a high drug-likeness score, although it will not qualify as a drug because of its high lipophilicity [113]. In our case, the drug-likeness, according to this platform’s predictions, was negative for all the hybrids, as well as for etoposide. All the hybrids from classes FQ-7-GLA and FQ-3-EA-GLA, as well as CF-3-EDA-GLA and MF-3-EDA-GLA, presented values lower than -10.

TheBD score assigns the probability that a compound will have BD > 10% in the rat, according to Yvonne C. Martin (2005) [114]. The bioavailability is measured from 0 to 1, but can also be represented as a percentage [115]. Any moiety with a bioavailability score of ≥0.55 is considered ideal and is absorbed very well by the body [116]. In our study, 12 hybrids out of 27 presented a BD score equal to or higher than 0.55, a promising aspect of their pharmacokinetic profile. It seems that there were representatives from all classes, but especially from class A (compounds NF-3-GLA, OF-3-GLA, LF-3-GLA, PF-3-GLA, CF-3-GLA), B (CF-7-GLA, NF-7-GLA), and C (NF-3-GLA-HA, PF-3-GLA-HA, CF-3-GLA-HA). All five of the compounds derived from norfloxacin presented a good BD score, while the hybrids derived from moxifloxacin were in the opposite situation.

Lead-likeness is a tactical guide for selecting chemical optimisation starting points that offer the best chance to deliver drug-like candidates [117]. Therefore, leads are required to have lower molecular complexity when compared to other drugs, a smaller number of rings and RB, a lower MW, and be less hydrophobic than drug-like molecules [74,118]. The online platform SwissADME predicted that all the proposed hybrids violated two lead-likeness rules, while etoposide failed only one [83].

SA is the synthetic accessibility score, which estimates the ease of synthesising a drug-like molecule for virtual screening by considering, among other aspects, the number of aromatic rings, stereocentres, macrocycles, or the size of the molecule. TheSA score varies from 1 to 10, with 1 being very easy to synthesise and 10 being very difficult [119,120,121]. This parameter was evaluated for all the hybrids because the subsequent synthesis of the proposed structures would depend heavily on it. According to the platforms’ results, the most difficult to synthesise from scratch was etoposide (6.27), followed by MF-3-EA-GLA (5.12) and MF-3-EDA-GLA (5.09). However, synthesising the theoretically formulated hybrids is more accessible because their synthesis involves connecting the two biologically active units (FQ and GLA) instead of starting from scratch.

### 3.3. Bioactivity and Anti-Cancer Potential

The bioactivity predicted with the Molinspiration platform indicated that all the hybrids had GPCR ligand properties. The score varied from −2 to 2 for all the predicted bioactivity parameters according to the platform instructions, and the higher the value of the score, the higher the probability that the particular molecule would be active [122]. All the hybrids had GPCR ligand score values higher than 0.2. Regarding the ion channel modulator activity, the kinase inhibition capacity, and the nuclear receptor ligand potential, the results were in the mentioned range, but the score was relatively low, presenting even negative values. In the case of the protease inhibitor activity of the hybrids, the highest values, above 0.2, were observed for the hydroxamic acid derivatives. Also, even though all the hybrids had positive values higher than 0.2 when assessing the enzyme inhibitory activity, the best results (higher than 0.5) were observed for the compounds from the FQ-3-GLA-HA class, especially for the MF-3-GLA-HA compound.

Our point of interest regarding enzymatic inhibition is the topoisomerase II inhibitory capacity of the hybrids, which was evaluated with the PASS Online platform. The platform offered information about the most likely mechanism of action for the hybrids and etoposide. The set condition was that the probability of being active (Pa) should be higher than that of being inactive (Pi) [123]. This platform suggested that our hybrids could manifest topoisomerase II activity, but the Pa score varied. The highest Pa score was shown by MF-7-GLA, 0.58, compared to 0.945 for etoposide. The hybrid was followed by MF-3-GLA (0.517) and CF-7-GLA (0.505). Since PASS Online offered targeted information on our enzyme of interest, we may also consider these results when outlining the compounds’ possible bioactivity potential. When analysing the Tc values, we could also see that MF-7-GLA and MF-3-GLA had a good score of 0.7, and this could also be an interesting aspect to observe. Also, we want to point out that the high enzyme inhibitory activity of the hybrids from class FQ-3-GLA-HA was most probably determined by the hydroxamic acid group, known for its intense anti-cancer activity. However, their mechanism of action involved the inhibition of histone deacetylase [84]. The enzyme inhibitory effect assessed with Molinspiration may have been the highest, but the probability of being active on topoisomerase II (according to PASS Online) was not that high; this may have resulted in good inhibitory activity, but maybe on enzyme histone deacetylase. Also, they were not among the hybrids with the best Fitted Score. This example confirms that we must consider the Tc, inhibitory activity, and docking score when analysing hybrids.

The CLC-Pred platform offered us information regarding the most probable cancer cell lines on which each hybrid had activity. Each hybrid was active on different cancer cell lines (Table S8—Supplementary Materials). The results obtained for Pa > 0.4 indicated that the cell lines on which most of the hybrids could exhibit cytotoxicity were as follows: in first place, adult acute myeloid leukaemia (OCI-AML2) and bladder carcinoma (UMUC3), with 23 of 27 hybrids; in second place, 22 hybrids presented activity on promyeloblast leukaemia (HL-60). Plasma cell myeloma (OPM-2) was indicated for 18 hybrids and pancreas adenocarcinoma (CAPAN-1) for 17 out of 27. The platform suggested cytotoxic activity on other cancer cell lines for the proposed hybrids, but fewer were active against them. According to CLC-Pred, etoposide is very active on lung small cell carcinoma (GLC4) and on lung carcinoma (A549), but also has activity in lung adenocarcinoma (SK-LU-1), breast carcinoma (ZR-75-1), adenosquamous lung carcinoma (NCI-H647), cervical adenocarcinoma (HeLa), blast phase chronic myelogenous leukaemia, BCR-ABL1 positive (K562/Adr), colorectal adenocarcinoma (SW48), breast carcinoma (MCF7), and squamous cell carcinoma (NCI-H520). Among these cell lines, SK-LU-1 and SW48 were also mentioned in the case of some hybrids: OF-3-GLA, LF-3-GLA, and MF-3-GLA for the first one, and OF-3-GLA-HA, LF-3-GLA-HA, and MF-3-GLA-HA for the second one.

We also want to highlight a few aspects regarding the hybrids’ specificity for the first three cancer cell lines (OCI-AML2, UMUC3, HL-60)—vosaroxin, a fluoroquinolone derivative, is used in phase III clinical trials against acute myeloid leukaemia [31,53]; all-trans retinoic acid–glutamate derivatives are effective against promyeloblastic leukaemia cell lines (HL60) [124]. The HL60 cell line was predicted for all the FQs (Pa > 0.4) using CLC-Pred; OCI-AML 2 was positive for pefloxacin, ciprofloxacin, ofloxacin, and levofloxacin and several cell lines predicted for the hybrids were also predicted for the FQs. Also, GLA and the hydroxamic acid derivative of GLA (gamma position) appeared to be efficient against UMUC3 and OCI-AML2 cell lines and against several other cell lines that coincide with the ones of the hybrids. Although GLA is not known for anti-cancer activity, we hypothesise that, at least in some cases, the hybrids tend to maintain the effects of the primary compounds.

### 3.4. Toxicity and Adverse Effects

The toxicity of the compounds was evaluated from multiple perspectives and based on different rules. According to Cramer’s rule, the hybrids are in class III, suggesting high toxicity. However, etoposide and FQs, such as ciprofloxacin, were included in the same class. Meanwhile, Kroes’s TTC classification estimated that the hybrids were not expected to represent a safety concern. Still, structural alerts for potential genotoxic carcinogenicity should be verified. The Verhaar scheme includes most of the hybrids in the fifth class, indicating that it is impossible to classify them according to these rules. The exceptions were CF-7-GLA and MF-7-GLA, which are in class 3, with unspecific reactivity. On the other hand, etoposide corresponded to the first class, being responsible for narcosis or baseline toxicity.

According to the PASS Online platform, the most frequent adverse effect among the hybrids was allergic contact dermatitis (23 out of 27 hybrids), followed by stomatitis in 19 cases and photoallergy dermatitis in 10 cases. Other adverse effects were predicted for the hybrids, but all of them were present in less than ten compounds. We evaluated the adverse effects of each FQ used in the hybrids’ structures and GLA with PASS Online (Pa > Pi). All the compounds were positive for the first three adverse reactions of the hybrids (with different Pa values). The FQs presented most of the adverse effects of the hybrids. According to the scientific literature, FQs are responsible for photosensitivity dermatitis because of their photohaptenic moiety, which can induce immune responses [125,126]. Also, there is a relationship between the chemical structure and the side effects among FQs [127]. We conclude that the hybrids maintained the adverse effects of the compounds from which they derived.

Carcinogenicity and mutagenicity were evaluated using Toxtree and Osiris Property Explorer. None of the compounds presented genotoxic or non-genotoxic toxicity and were also not responsible for tumorigenesis. Osiris indicated negative results for all the hybrids when predicting mutagenicity, while the Ames test indicated that hydroxamic acid derivatives have mutagenic activity (*Salmonella typhimurium*).

Regarding the irritative or corrosive properties, Osiris provided negative results for all the hybrids, and Toxtree also indicated that the hybrids are not corrosive to the skin. Also, the hybrids do not produce R34 and R35 types of skin corrosion to the eyes (R34 means that the substance can cause burns, and R35 indicates severe burns [128,129,130]). In the case of etoposide, no R34, R35, R36—significant eye irritation (middle category of irritancy)—or R41—severe eye irritation (risk of serious damage to eyes)—lesions were indicated [131,132].

Regarding the effect on the reproductive system, most of the results were negative, except for the pefloxacin-derived hybrids, which seemed to have medium-risk fragments for this type of toxicity. Because of this prediction, we also evaluated the reproductive system toxicity for pefloxacin, and the same result was obtained.

Using several platforms/programmes that give different information regarding toxicity could be quite confusing. That is why we comment on a few aspects:

- According to Kroes TTC, although the hybrids are not expected to represent a safety concern, the Toxtree programme indicated to verify the potential genotoxic carcinogenicity—the same programme (Toxtree) permits evaluating genotoxic carcinogenicity, and negative results were found for all the hybrids;
- Both Toxtree and Osiris Property Explorer assessed the mutagenicity, and there was a consensus between the results for all the hybrids except for the hydroxamic acid derivatives;
- Regarding producing irritation or corrosion, both Toxtree and Osiris Property Explorer offered negative results;
- Even if, according to Cramer’s rule, the hybrids were classified as having high toxicity, the same result obtained for FQ did not necessarily indicate a safety concern, especially since, according to the Verhaar scheme, they could not be classified as not offering a relevant result for the study; we consider that the results predicted with PASS Online could be more pertinent, which is more specific on which are the most probable adverse/toxic effects that may occur.

These toxicity results will not prevent us from synthesising any of the hybrids. In vitro and in vivo tests will be conducted to confirm/infirm these platforms’ predictions after synthesising the most promising compounds in this study.

### 3.5. Self-Docking of Etoposide and Docking of the Hybrids to Human Topoisomerase II Beta-DNA Complex

We used the FORECASTER platform to proceed with the docking study. The FORECASTER platform includes several modules for different steps. Therefore, the PREPARE and PROCESS tools were used to prepare and set the protein for docking; SMART is a module for ligand preparation, and FITTED is the docking programme.

In this type of experiment, a self-docking step should first be performed as a sanity check to validate the method and demonstrate that the software can model and select the conformation of a ligand bound to the protein target. Self-docking means using the native ligand from the crystal structure and trying to dock it back to the protein correctly. This step should always be performed if we have an available crystal structure with a co-crystalized ligand (human topoisomerase II beta co-crystalized with DNA and etoposide, in our case).

The first step in the protein self-docking procedure was to take the protein–ligand complex from the PDB file and prepare the corresponding files using the PREPARE tool. Thus, we ensured that the protein file was consistent and could be used further. The ligand residue must be identified and extracted from the protein–ligand complex to define the active site. Therefore, we identified the ligand residue and generated protein and ligand files using the PREPARE tool. These files were ready for molecular modelling tools, including PROCESS and SMART.

The extracted ligand was further prepared with the SMART (Smart Molecule Atom-typing and Rotatable Torsion Assignment) tool, ready for FITTED docking. Also, PROCESS (Protein Conformational Ensemble System Setup) took the prepared protein file (result after adding hydrogens to the protein). It created all the necessary binding site files and interaction sites that would be used for scoring the dock poses (FITTED tool). Also, another file was created, which was the actual file that FITTED used for docking (the binding site). FITTED (Flexibility Induced Through Targeted Evolutionary Description) is a tool that can dock small molecules to proteins and predict the ligand binding mode and optimal conformation of proteins. FITTED does not use grids, but spheres were used to approximate the cavity space, as it was found to be more convenient regarding the required docking time.

When self-docking etoposide to the protein–DNA complex (after three runs), the results obtained for the best docking scores were as follows: the internal energy (−95.7 kcal/mol); the Rank Score (−32.3)**,** which iswas a set of scoring functions based on energy terms and other termswhich areall scaled to model the observed binding free energies better—the lower the Rank Score the better; the Match Score (50.1), which is a measure of how well the ligand fit inside the active site, based on the interaction sites generated by PROCESS—the higher the score, the better the ligand matched the protein interaction sites; the Fitted Score (−40.3), a score based on the Rank Score and Match Score, representing the measure of how the ligand interacted with the active site from an energetic and geometric point of view. The Fitted Score is the measure of binding affinity, and it is unitless, reflecting the relative ranking. It is considered the metric of choice when ranking compounds. Also, all these metrics are used to rank ligands within the same target.

In addition, evaluating the RMSD was essential in the case of self-docking. The self-docking step is mandatory when using a protein co-crystalized with a ligand (etoposide, in our case); the result of this process indicates the accuracy of the platform for docking the extracted ligand in the same binding site as the one for the crystallised form of the ligand. An RMSD value below 2 Å indicates that the platform can detect the correct binding site of the reference compound (etoposide) from the crystallised complex and re-dock it properly in the same place. Since our purpose was to evaluate the interaction of the hybrids with the binding site of the reference compound, an accurate detection of the binding site and the validation of the platform’s ability to perform docking correctly using this specific protein was mandatory. If an RMSD value is more significant than 2 Å, it may indicate that the software is unsuitable for docking to that protein target. In our case, the RMSD value was 0.56 Å, and such a small value means that FITTED was performing very well at docking to this specific protein. When comparing the docked pose to the original ligand (Figure 3), we can observe how well the two matched [133,134,135,136].

After performing the self-docking step, we used the FORECASTER platform to conduct the docking study for all 27 hybrids, using the same binding site and interaction site files generated in the self-docking step. The best results (the best Fitted Score) were obtained for the hybrid OF-3-EDA-GLA (−32.4), followed by MF-7-GLA (−30.1) (Table 10 and Figure 5 and Figure 6). According to these predicted results, we can observe that none of the proposed hybrids exceeded the Fitted Score obtained for etoposide (−40.3), resulting in the reference compound having the best interaction with the active site.

Among all the scores predicted by the platform, the Fitted Score was the indicator for the best interaction with the active site, including the Match Score (matching in the active site) and Rank Score (related to the binding free energy). So, we can affirm that a lower Fitted Score is correlated with an increased binding affinity. However, we do not affirm that it directly correlates with the enzyme inhibitory activity. According to the scientific literature, binding affinity is the change in the free energy associated with a binding process. The binding affinity measures how strong the interaction between a ligand and a protein is. Therefore, it is often directly related to the potency of the ligand [137]. We emphasise that the mechanism of etoposide and fluoroquinolones as topoisomerase II inhibitors involves forming irreversible bonds with the DNA–protein complex. Thus, they stop the catalytic cycle of type II topoisomerase after DNA cleavage and increase the amount of topoisomerase II-DNA cleavage complexes. Therefore, there may be a possibility that a better interaction with the enzyme results in increased inhibitory activity. However, PASS Online predicted a better topoisomerase II activity for the other hybrids with a lower Fitted Score.

We also used the software AutoDock Vina and AutoDock to perform the docking of all 27 hybrids to the topoisomerase II beta-DNA complex, for the high accuracy of our results (Table 11). All the compounds from the B class (FQ-7-GLA) were predicted to have the highest interaction with the target. From the respective compounds, MF-7-GLA had the most favourable binding energy from the whole series of compounds, with ΔG = −13.58 kcal/mol. The relatively large number of conformations for the respective compound (10 conformations) suggested higher confidence and reproducibility in the binding mode of the MF-7-GLA. Other compounds such as CF-3-GLA, OF-3-EA-GLA, LF-3-EA-GLA, and OF-3-EDA-GLA were also the top binders of the respective series, exhibiting good values for ΔG (less than −11.5 kcal/mol), as well as for top binding conformation, but also for the mean ΔG of the cluster containing the top binding conformation.

All FQ-7-GLA and FQ-3-EA-GLA hybrids have a high binding affinity (more than −14.46 kcal/mol), taking the total intermolecular (ligand–target) energy into account. A deeper analysis of the data, taking into account the energy decomposition, indicated that the good binding of the respective compounds was mainly driven by the electrostatic interactions (between −6.02 and −7.54 kcal/mol). Other compounds, such as CF-3-GLA, FQ-3-EA-GLA class, CF-3-EDA-GLA, OF-3-EDA-GLA, and LF-3-EDA-GLA also had high intermolecular energy (more than −14.42 kcal/mol), but this was mainly driven by a mixture of van der Waals, hydrogen bonding, and desolvation energy (more than −10.58 kcal/mol) rather than electrostatic interactions (maximum −6.26 kcal/mol). A higher torsional energy was identified for all the compounds from the FQ-3-EA-GLA and FQ-3-EDA-GLA classes and interestingly, for all the moxifloxacin-derived compounds, in every dataset of compounds the respective compounds had the highest torsional energy.

Correlating the results, the hybrids that stood out with high binding affinity for the topoisomerase II beta-DNA complex according to all three software (in the top 25% of results in each case) were MF-7-GLA and OF-3-EDA-GLA.

Regarding the interactions between the etoposide and the target enzyme–DNA complex, the alkyl and Pialkyl bonds between the ligand and MET 782, ARG 503, and the residues Adenine 12 (DA*12), Guanine 10 (DG*10), Guanine 13 (DG*13), Thymine 9 (DT*9), and Cytosine 8 (DC*8) were observed. Hydrogen bonds were made between ASP 479 and the phenolic hydroxyl group, GLN 778 and the lactonic oxygen atom, and Guanine 13 (DG*13) and the alcoholic hydroxyl group.

OF-3-EDA-GLA presented hydrogen bonds between GLN 778 and the keto group of the hybrid, but also between ARG 503 and the amidic carbonyl group in position C3 of the quinolinic ring and between residue Thymine 9 (DT*9) and the amino group (from GLA rest). Pi-alkyl interactions between the ligand and residues Cytosine 8 (DC*8) and Adenine 12 (DA*12) were observed. A Pi-Pi stacked interaction between Adenine 12 (DA*12) and the aromatic ring is highlighted.

MF-7-GLA formed a hydrogen bond with GLN 778 through the amidic oxygen of the ligand (the amidic group formed between the rest in position C7 of MF and GLA). Also, the primary amino group formed hydrogen bonds with residues Guanine 7 (DG*7) and Guanine 13 (DG*13). Carbon-hydrogen bonds with Guanine 13 (DG*13) and Thymine 9 (DT*9) were formed. Also, several alkyl and Pi-alkyl interactions are highlighted, including with ARG 503. This amino acid was also involved in a Pi-cation interaction with the aromatic ring.

When comparing the ligand–enzyme–DNA complex interactions, we can observe that GLN 778 is a key amino acid, asboth etoposide and the two hybrids formed hydrogen bonds. Also, ARG 503, even though it was involved in different types of interactions, appeared in all three cases. As the ligands were involved in several interactions with the two amino acids (GLN 778 and ARG 503), we can affirm that a compound with high hydrophilicity is the most suitable candidate as a ligand; this aspect was consistent with the TPSA values that were among the highest for the two analysed hybrids.

### 3.6. The Hybrid with the Most Promising Predicted Properties. Our Proposal for Its Synthesis

In silico studies are a filtering method of a library of theoretically proposed compounds; this helps minimise the time and effort required for drug development. Therefore, after analysing all the predicted results, we concluded that the hybrid that would be the ideal candidate as a lead compound was MF-7-GLA (B6). We affirm this because of some physicochemical properties with close values to the ones of etoposide (Csp3 fraction, MR, TPSA, geometric span) and the Tc of 0.7, which indicates a substantial molecular resemblance with the reference compound. When predicting the biological activity, specifically the ability to inhibit the type II topoisomerase (PASS Online platform), MF-7-GLA presented the highest score among all the hybrids (Pa of 0.58). The platform Molinspiration was consistent with these findings when predicting the enzyme inhibitory activity of the hybrids, although in more general terms, with a score of 0.49. Our choice is also supported by the good Fitted Score that the hybrid presented (−30.1)—the second one, after the hybrid OF-3-EDA-GLA (FORECASTER platform)—and the ΔG values obtained with AutoDock Vina (−10.5) and AutoDock software (−13.58), suggesting a high binding affinity. All these data indicated that MF-7-GLA was the hybrid with the most promising predicted properties in our study and the main one that should be considered for synthesis.

Although both MF-7-GLA and OF-3-EDA-GLA presented favourable properties, we have considered MF-7-GLA instead of the second one as a potential leader compound for the following reasons:

- The higher Tc;
- Although both of them had high similarity for physicochemical properties with etoposide, MF-7-GLA had closer values to the reference compound for some parameters (Csp3 fraction, MR, H-bond donors, geometric span);
- Better score for the enzyme inhibitory capacity (Molinspiration);
- The best Pa for topoisomerase inhibitory activity among all the hybrids (PASS Online);
- Although OF-3-EDA-GLA had a better predicted Fitted Score, and a lower ΔG obtained with AutoDock Vina, the values for MF-7-GLA were very close to the ones of the OF derivative. Also, MF-7-GLA had a lower ΔG than OF-3-EDA-GLA, according to AutoDock.

**Figure 8:**
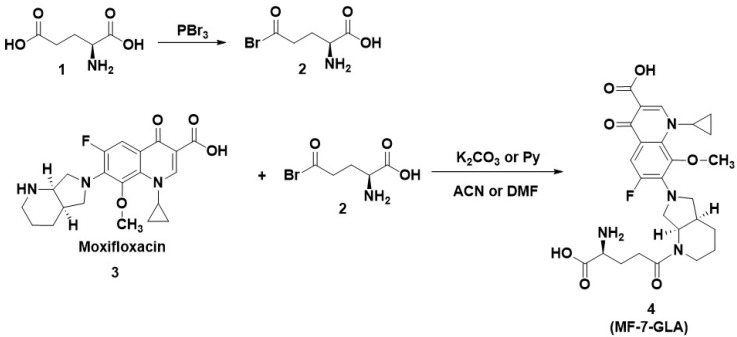
Synthesis proposal for hybrid MF-7-GLA.

In this regard, we proposed, in Figure 8, the synthesis approach for hybrid MF-7-GLA starting from commercially available moxifloxacin and GLA. The first step involves the synthesis of glutamyl bromide (2) (much more reactive [138]) from GLA and PBr_3._ The next step is the acylation of the secondary nitrogen of moxifloxacin [139], using a base for proton trapping, in a polar aprotic solvent, which should lead to the hybrid MF-7-GLA.

We also shaped some synthesis possibilities for OF-3-EDA-GLA—the hybrid with the best Fitted Score (−32.4) and good physicochemical properties (for example the McGowen volume and TPSA) and the hydroxamic acid derivatives. Good results were obtained for the FQ-3-GLA-HA class with the Molinspiration platform for the enzyme inhibitory capacity and the Tc (MF-3-GLA-HA having the highest score predicted with Molinspiration and the highest Tc among all the hybrids) (Figures S7 and S8—Supplementary Materials section). These compounds could also be taken into consideration when developing this research direction.

## 4. Materials and Methods

### 4.1. Design of the Hybrids

After analysing the scientific literature and FQs’ and GLA’s structures, we designed a series of new hypothetical hybrids of FQ-AGL with essential pharmacophoric characteristics for type II topoisomerase inhibition. Therefore, 27 FQ-AGL hybrids were created using the Biovia Draw 21.1 programme [140]. The FQs used for the computational design were ciprofloxacin (CF), norfloxacin (NF), pefloxacin (PF), ofloxacin (OF) (second generation), levofloxacin (LF) (third generation), and moxifloxacin (MF) (fourth generation). The library of compounds contains the following types of hybrids:

- Class FQ-3-GLA/Class A: Hybrids in which GLA was linked to the FQs carboxyl group (position 3) through the amino group (amide bond) (hybrids A1–A6);
- Class FQ-7-GLA/Class B: Hybrids in which GLA was linked via the carboxyl group (gamma position) to the nitrogen atom of the heterocycle in position 7 of the FQs—for CF (hybrid B1), NF (hybrid B2), and MF (hybrid B6);
- Class FQ-3-GLA-HA/Class C: Hybrids in which GLA was linked to the FQs carboxyl group (position 3) through the amino group (amide bond), with a hydroxamic acid functional group (HA) in the gamma position of GLA (hybrids C1–C6);
- Class FQ-3-EA-GLA/Class D: Hybrids in which GLA (the carboxyl group in the gamma position) was linked to the FQs carboxyl group (position 3) through an ethanolamine (EA) bridge (hybrids D1–D6);
- Class FQ-3-EDA-GLA/Class E: Hybrids in which GLA (the carboxyl group in the gamma position) was linked to the FQs carboxyl group (position 3) through an ethylenediamine (EDA) bridge (hybrids E1–E6).

**Figure 9:**
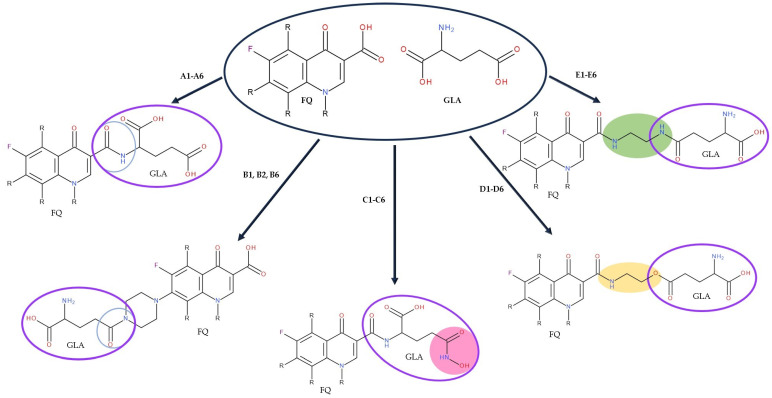
The general structures of each class of hybrids.

The general structures of each class of hybrids are presented in Figure 9, and the classification of the hybrids and their IDs are presented in Table 12.

After designing the 27 hybrids, we applied an original virtual screening protocol to analyse them. We assessed the physicochemical and pharmacokinetic properties, biological behaviour, anti-cancer activity, and toxicological profile.

### 4.2. Prediction of Physicochemical, Pharmacokinetic, and Structural Properties, Biological Behaviour, Anti-Cancer Activity, and Toxicological Profile. The Tanimoto Coefficient

The hybrids were characterised in terms of structure, physical, and chemical properties (number of HA, HAA, Csp3 fraction—the ratio between the number of sp3-hybridised carbon atoms versus the total carbon number of the molecule, rotatory bonds, hydrogen acceptors and donors, molar refractivity, and topological polar surface area) using the SwissADME platform [74,119].

Also, the SwissADME platform was used to predict water solubility [141,142], lipophilicity and partition coefficients, gastrointestinal absorption, permeability through the blood–brain barrier, and interaction with P glycoprotein. The enzyme inhibitory effect on several isoforms of CYP450, the number of broken rules, according to Lipinski, Eagan [143], Veber [144], Ghose [145], and Muegge [146] (Supplementary Materials section—Figure S1), the bioavailability score, the synthetic accessibility, and the lead-likeness were also evaluated [117]. The solubility of the hybrids in water was predicted using the online platform Aquasol [147].

The MarvinSketch programme (v 23.11) helped to evaluate some structural and physicochemical properties (pKa, isoelectric point, molar polarizability). It was possible to characterise the hybrids in terms of lipophilicity (logP, logD at pH 7.4, HLB), the CNS MPO score, and the best conformations of the hybrids [101].

Regarding the bioactivity of the hybrids, we used the online platform Molinspiration (v 2022.08), with which we analysed the activity of G protein-coupled receptors and nuclear receptors, as well as the ion channel modulator capacity, and kinase, protease and enzyme inhibitory capacity [122]. The PASS Online platform [123] provided information about the most likely mechanisms of action by which the hybrids manifested their biological activity.

The CLC-Pred 2.0. online platform evaluated the anti-cancer activity by suggesting cancer cell lines for which the hybrids could show an anti-cancer effect [148].

The toxicity of the hybrids was evaluated from multiple perspectives using several platforms and programmes. The Gusar platform (http://www.way2drug.com/gusar/acutoxpredict.html (accessed on 23 May 2024)) [149] provides information on acute toxicity in rodents (LD50 (mg/kg)) after oral, intravenous, intraperitoneal, and subcutaneous administration and about the classification of chemical compounds according to the OECD Project (Classification of Chemicals by OECD Project) [128].

The Toxtree programme v 3.1.0.1851 [150] assesses the degree of toxicity of a substance according to Cramer’s rule [151], but also according to Kroes TTC (thresholds of toxicological concern) [152] and after the Verhaar scheme [153,154]. The programme also provides information related to carcinogenicity, genotoxicity, and in vitro mutagenesis (Ames test) [155,156,157], but also to skin [128,129,130] and eye irritation [158,159,160,161]. At the same time, the biodegradability degree of the hybrids, their binding alerts to protein and DNA [162,163], and the CYP450-mediated metabolism were predicted using Toxtree [164].

The Osiris Property Explorer programme was used to complete the data about toxicity with details about mutagenesis and tumorigenesis risk, as well as the possibility of producing irritations and affecting the reproductive system [113]. Also, this programme offered information regarding the drug-likeness of the compounds [117].

Using the SmartCyp (v 3.0) platform, the hybrids’ sites involved in metabolism via CYP3A4, CYP2D6, and CYP2C9 isoforms were predicted [106,107].

We also assessed the similarity of our hybrids to etoposide in terms of their functional groups/structural features (the Tanimoto coefficient) using the FORECASTER platform (SELECT function) [165]; the McGowen volume and molecular span were predicted with this programme to complete the physicochemical profile determined by using the programmes and platforms already mentioned above.

### 4.3. Self-Docking and Docking Study

To analyse the interaction of the hybrids with the human topoisomerase II enzyme, we used the human topoisomerase II beta in a complex with DNA and etoposide provided by PDB (PDB complex code: 3QX3). PDB provides a high-resolution crystal structure of the DNA-binding and cleavage core of the human TOP2 b isoform (residues 445 to 1201) in a complex with DNA and the anti-cancer drug etoposide (resolution 2.16 Å) [88]. We used the etoposide-co-crystalised enzyme because FQs and etoposide act as topoisomerase II inhibitors through the same mechanism. Therefore, we planned to observe if our hybrids, derived from FQs, could interact with the target enzyme and bind to it like etoposide, a well-known compound used in anti-cancer therapy. Then, we conducted the docking study of our proposed hybrids to the enzyme after validating the method by docking the etoposide to the target enzyme (self-docking) with the FORECASTER platform. The final step was to proceed with docking our hybrids to the target enzyme (using the FORECASTER platform), assessing if they could interact with the type II topoisomerase in the same binding site and interaction sites as etoposide, using the same programme.

In addition, for high accuracy of the results, molecular docking was also performed using the software AutoDock Vina, v 1.1.2 [166] and AutoDock, v 4.2 [167]. The molecular docking study using AutoDock Vina and AutoDock was performed using the previously reported protocol by our group, using AutoDockTools 1.5.6, which conducted the removal of other molecules, proper protonation, and charge assignment [167,168]. The search space was set as a cube with sides equal to 22 Å for AutoDock Vina and 60 for AutoDock, with 0.375 spacing. The centre of the search space was set to x = 32.783, y = 95.265, and z = 52.584 for both software. The search space was configured as presented, to include the previously bound etoposide in the crystal structure of the targeted complex PDB 3QX3 [88]. AutoDock Vina was set to give 20 poses for each ligand, while AutoDock was set to give 50 poses for each ligand, grouped in 2 Å clustering using the built-in function. For the top binding conformation of each compound given by AutoDock, an energy decomposition analysis was performed using the built-in function.

The structures were visualised with the programme Biovia Discovery Studio 2021, v 21.1.0.20298 [169] and Chimera, v 1.10.2 [170].

## 5. Conclusions

In this paper, we theoretically formulated hybrids between different FQs and GLA with possible anti-cancer properties based on the idea that FQs and etoposide are topoisomerase II inhibitors. Therefore, using computational methods, we evaluated the hybrids from multiple perspectives and outlined a complex profile for them, trying to approach this evaluation by comparing them with etoposide. We used several online platforms and programmes to predict different properties. Also, we performed a docking study to observe if the hybrids would interact with the human topoisomerase II beta-DNA complex in the same way as the etoposide.

According to SwissADME, all the hybrids violated at least one of the Lipinski/Ghose/Veber/Egan/Muegge rules or lead-likeness criteria, similar to some essential drugs used in therapy. The hybrids presented a relatively hydrophilic profile with a decreased capacity to cross some critical biological membranes. Although we considered it positive that they did not pass the BBB, assuming that they did not affect the CNS, some structural improvements may be desired to enhance gastrointestinal absorption.

According to the PASS Online platform, all the hybrids presented possible topoisomerase II inhibitory activity, and the most targeted cancer cell lines, according to the CLC Predictor, were adult acute myeloid leukaemia, bladder carcinoma, and promyeloblast leukaemia.

The toxicological profile was contoured by several specific rules, such as Cramer’s rule, which classified the hybrids and etoposide in the class III of toxicity (high). However, the same result was obtained for CF. According to some other rules, hybrids were not expected to be a safety concern (Kroes TTC), or it was impossible to classify them according to these rules (Verhaar scheme). The hybrids generally did not present carcinogenicity or mutagenicity risk, irritation or corrosion effect on the skin and eyes, or adverse effects on the reproductive system. Some of the most common adverse or toxic effects, according to PASS Online, were allergic contact dermatitis, stomatitis, or photoallergy dermatitis.

Regarding the molecular docking study, the step of self-docking the co-crystalized etoposide to the enzyme, with an RMSD of 0.56 Å, confirmed that the FORECASTER platform was suitable for performing docking with the human topoisomerase II beta-DNA complex. When conducting the docking study using the same binding site and interaction sites (FORECASTER platform) as for etoposide, the hybrids with the best results (Fitted Score) were OF-3-EDA-GLA and MF-7-GLA. These results were also supported by AutoDock Vina and AutoDock software, which indicated a high binding affinity for both the hybrids.

The hydroxamic acid derivatives were the hybrids that presented good scores for some properties, including the Tc and bioactivity. Five among all six of them had a Tc of at least 0.7, with the MF derivative having the highest score of all the hybrids, of 0.72. Also, they presented the highest scores for the enzyme inhibition capacity, according to the Molinspiration platform (higher than 0.5), especially the MF-3-GLA-HA.

However, among all the hybrids, MF-7-GLA would be the ideal candidate as a lead compound. The high similarity for physicochemical properties with etoposide (Csp3 fraction, H-bond donors, MR, geometric span), the good Tc (0.7), the good score for the enzyme inhibitory capacity (0.49) assessed with Molinspiration, the best Pa for topoisomerase II inhibitory activity, and the second best Fitted Score assessed with the FORECASTER platform supported by very good ΔG values predicted by AutoDock Vina and AutoDock software are our reasons for selecting this hybrid as the one with the most promising predicted properties.

In conclusion, we consider that FQs and GLA have advantageous structures for obtaining hybrids with biological potential, but the topic should be further studied. Among all the proposed hybrids, MF-7-GLA is the one for which we consider continuing this study with the synthesis step and in vitro bioactivity evaluation.

## 6. Limitations

- One of the aspects that we consider a limitation of this study is that some of the platforms or programmes did not make the difference between optical isomers. As a result, some variations in the predicted properties could exist for some hybrids;
- Another limitation is the difficulties in correlating the predictions obtained for different properties in order to select a potential lead compound; this aspect derives from the fact that there was not a single hybrid that presented the most favourable results for all the estimated properties, making the selection process of the most promising hybrids more difficult.

## Figures and Tables

**Table 1:** Topoisomerase II inhibitors and the type of cancer cell they act on.

No.	Class	Compounds	Cancer Cell Type	Reference
1.	Anthracenediones	Mitoxantrone	Bladder cancer, breast cancer, non-Hodgkin lymphoma, acute myeloid leukaemia	[20,23,24,25]
2.	Anthracyclines	Doxorubicin	Breast cancer, lung cancer, gastric cancer, ovarian cancer, thyroid cancer, bladder cancer, Hodgkin and non-Hodgkin lymphoma, multiple myeloma, sarcoma (approved by FDA in 1974)	[20,21,23,26,27]
		Epirubicin	Breast cancer	[21,28,29]
3.	Isothiazoloquinolones	A-65281	-	[20]
4.	Quinolones	Vosaroxin	Acute myeloid leukaemia (Phase III clinical trials)	[20,23,30,31]
5.	Semisynthetic derivatives of podophyllotoxin	Etoposide	Refractory testicular tumours, small cell lung cancer, lymphoma, glioblastoma, non-lymphocytic leukaemia, sarcoma, neuroblastoma, Hodgkin lymphoma, stomach cancer	[20,21,23,32]
		Teniposide	Refractory acute lymphoblastic leukaemia	[33]
6.	Sulphonamide	Amsacrine	Acute myeloid leukaemia, malignant lymphoma	[20,23,34]
7.	Thiobarbituric derivatives	Merbarone	-	[20,35,36]

**Table 2:** The ID, chemical structure, and molecular weight corresponding to each hybrid. The Tanimoto coefficient of the hybrids evaluated using etoposide as a reference compound.

Hybrid Class Abbreviation/ID	Hybrid Abbreviation/ID	Hybrid Structure	Molecular Weight	Tanimoto Coefficient
FQ-3-GLA/A	CF-3-GLA/A1	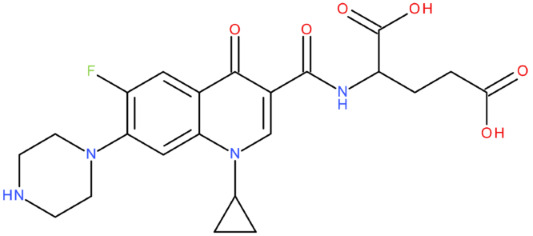	460.46	0.67
	NF-3-GLA/A2	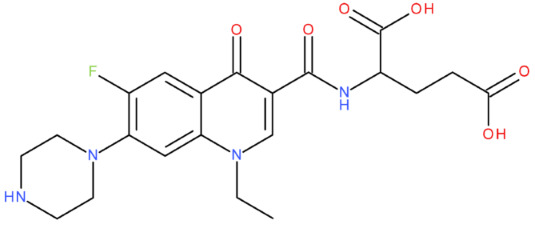	448.44	0.67
	PF-3-GLA/A3	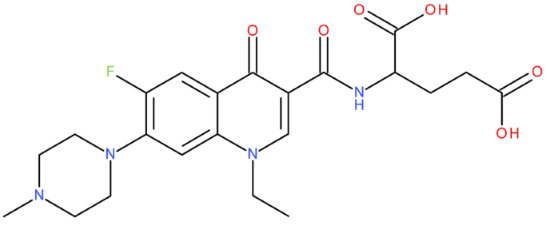	462.47	0.67
	OF-3-GLA/A4	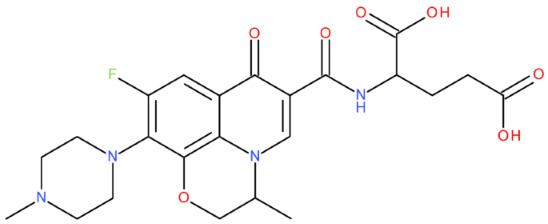	490.48	0.68
	LF-3-GLA/A5	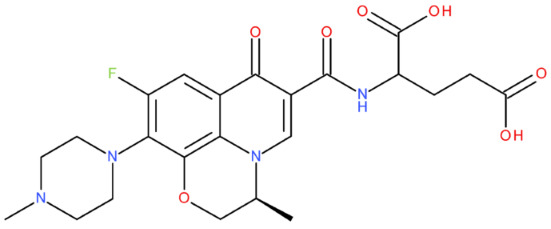	490.48	0.68
	MF-3-GLA/A6	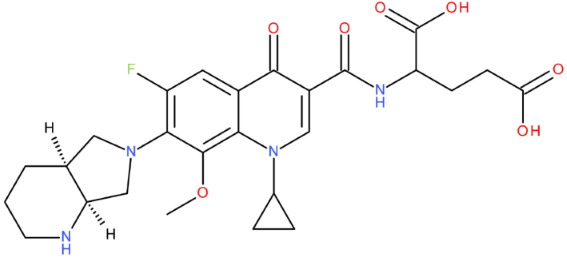	530.55	0.7
FQ-7-GLA/B	CF-7-GLA/B1	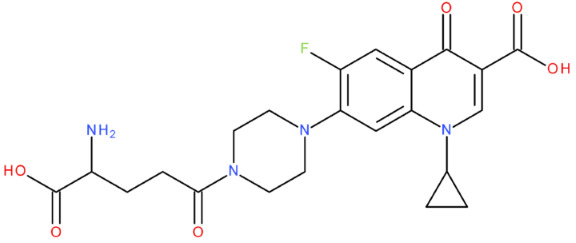	530.55	0.67
	NF-7-GLA/B2	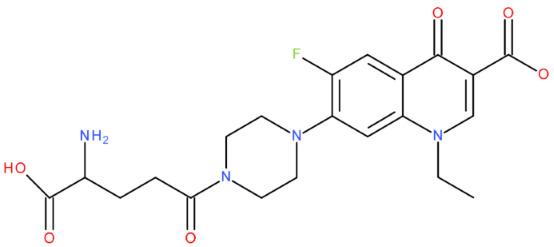	448.44	0.68
	MF-7-GLA/B6	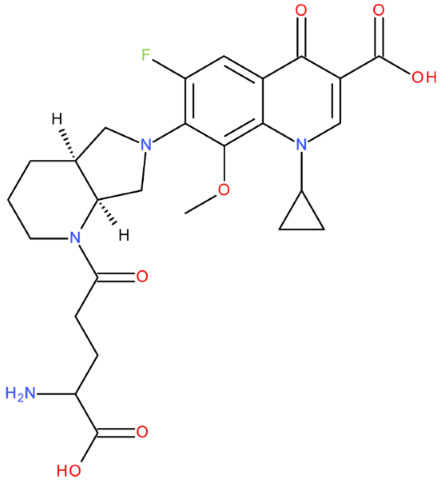	530.55	0.7
FQ-3-GLA-HA/ C	CF-3-GLA-HA/C1	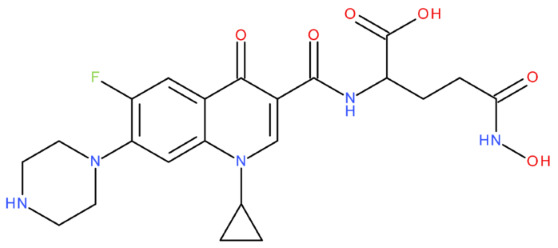	475.47	0.69
	NF-3-GLA-HA/C2	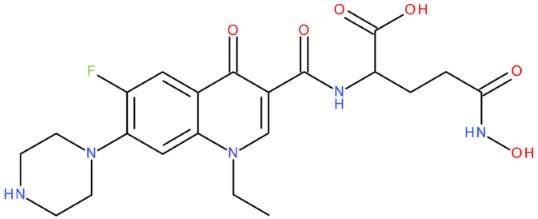	463.46	0.7
	PF-3-GLA-HA/C3	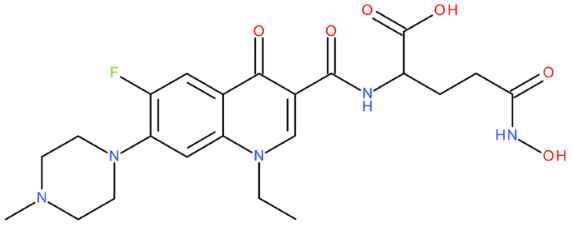	477.49	0.7
	OF-3-GLA-HA/C4	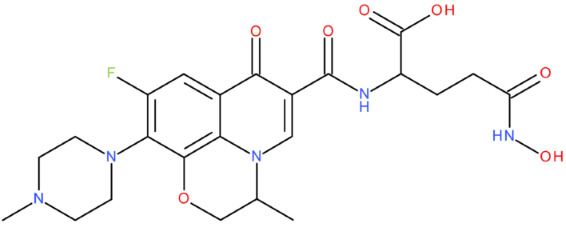	505.50	0.71
	LF-3-GLA-HA/C5	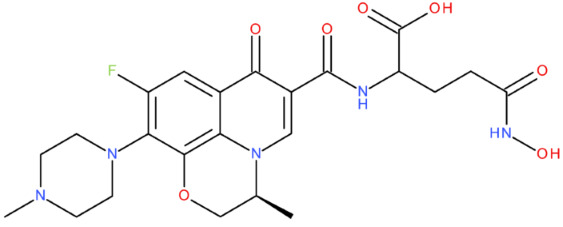	505.50	0.71
	MF-3-GLA-HA/C6	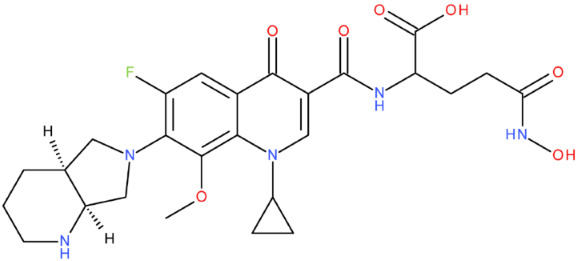	545.56	0.72
FQ-3-EA-GLA/D	CF-3-EA-GLA/D1	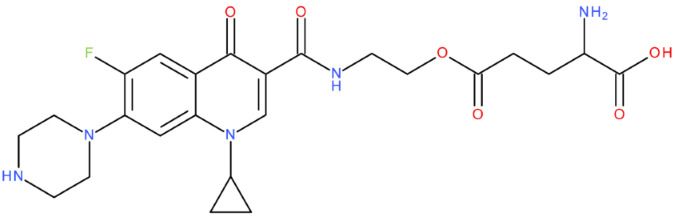	503.52	0.66
	NF-3-EA-GLA/D2	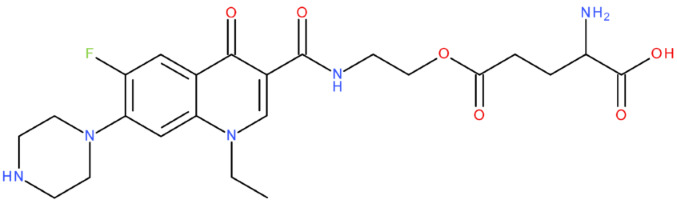	545.56	0.66
	PF-3-EA-GLA/D3	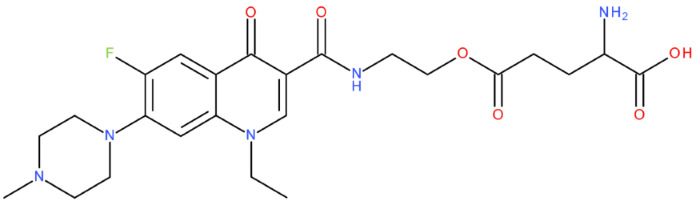	533.55	0.66
	OF-3-EA-GLA/D4	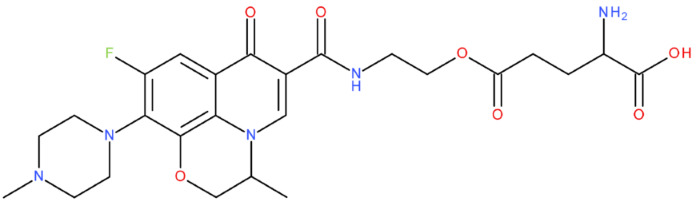	533.55	0.67
	LF-3-EA-GLA/D5	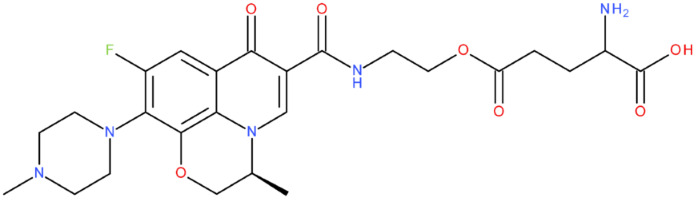	533.55	0.67
	MF-3-EA-GLA/D6	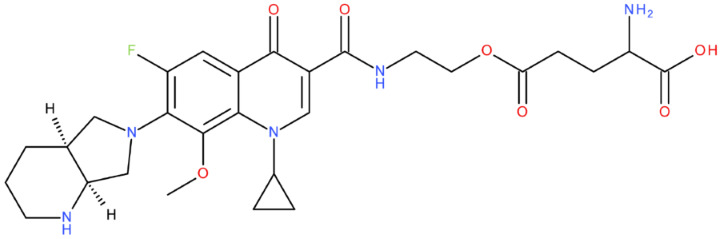	573.61	0.69
FQ-3-EDA-GLA/E	CF-3-EDA-GLA/ E1	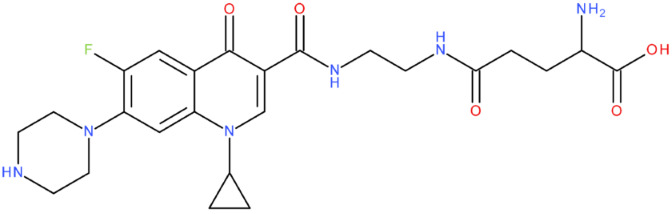	502.54	0.66
	NF-3-EDA-GLA/E2	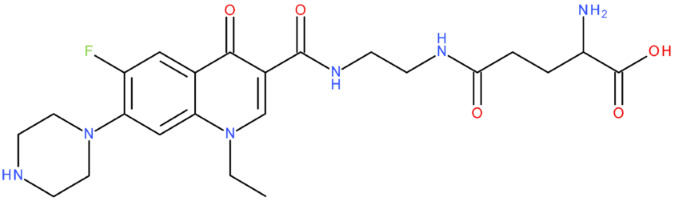	490.53	0.67
	PF-3-EDA-GLA/E3	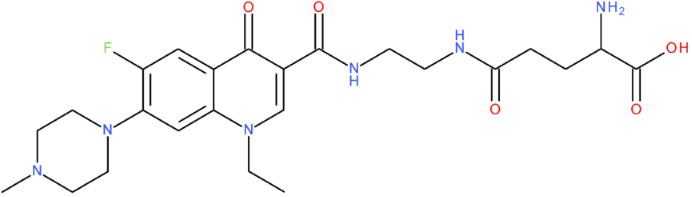	504.55	0.67
	OF-3-EDA-GLA/E4	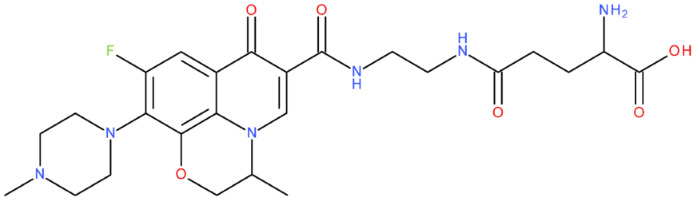	532.56	0.68
	LF-3-EDA-GLA/E5	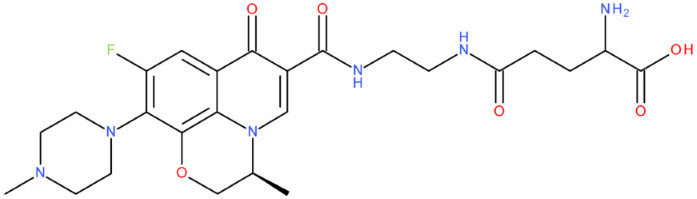	532.56	0.68
	MF-3-EDA-GLA/ E6	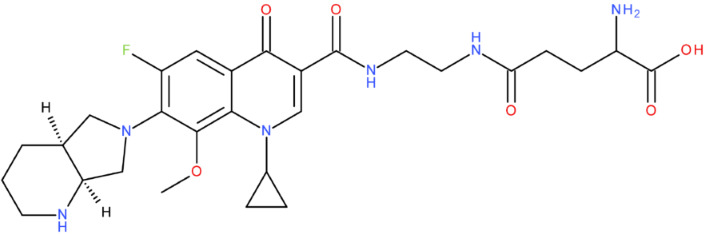	572.63	0.69

The Tanimoto coefficient was evaluated using the MACCS fingerprint.

**Table 3:** Structural and physicochemical properties (SwissADME).

Compound/ID	HA	HAA	Fraction Csp3	RB	H-Bond Acceptors	H-Bond Donors	MR	TPSA	McGowan Volume (mL/mol) *	Geometric Span *
CF-3-GLA/A1	33	10	0.45	9	8	4	124.05	140.97	317.75	9.530
NF-3-GLA/A2	32	10	0.43	9	8	4	121.36	140.97	314.52	9.536
PF-3-GLA/A3	33	10	0.45	9	8	3	126.26	132.18	328.61	10.300
OF-3-GLA/A4	35	10	0.48	8	9	3	130.64	141.41	337.71	10.235
LF-3-GLA/A5	35	10	0.48	8	9	3	130.64	141.41	337.71	10.235
MF-3-GLA/A6	38	10	0.54	10	9	4	142.85	150.20	369.12	10.571
CF-7-GLA/B1	33	10	0.45	8	8	3	124.05	146.17	317.75	10.603
NF-7-GLA/B2	32	10	0.43	8	8	3	121.36	146.17	314.52	10.482
MF-7-GLA/B6	38	10	0.54	9	9	3	142.85	155.40	369.12	9.795
CF-3-GLA-HA/C1	34	10	0.45	10	8	5	126.01	153.00	329.88	9.721
NF-3-GLA-HA/C2	33	10	0.43	10	8	5	132.32	153.00	326.65	9.803
PF-3-GLA-HA/C3	34	10	0.45	10	8	4	128.22	144.21	340.74	10.573
OF-3-GLA-HA/C4	36	10	0.48	9	9	4	132.60	153.44	349.84	10.509
LF-3-GLA-HA/C5	36	10	0.48	9	9	4	132.60	153.44	349.84	10.506
MF-3-GLA-HA/C6	39	10	0.54	11	9	5	144.81	162.23	381.25	10.857
CF-3-EA-GLA/D1	36	10	0.50	12	9	4	135.89	155.99	360.21	13.005
NF-3-EA-GLA/D2	35	10	0.48	12	9	4	133.20	155.99	356.98	12.888
PF-3-EA-GLA/D3	36	10	0.5	12	9	3	138.10	147.20	371.07	13.222
OF-3-EA-GLA/D4	38	10	0.52	11	10	3	142.48	156.43	380.17	13.515
LF-3-EA-GLA/D5	38	10	0.52	11	10	3	142.48	156.43	380.17	13.501
MF-3-EA-GLA/D6	41	10	0.57	13	10	4	154.69	165.22	411.58	14.256
CF-3-EDA-GLA/E1	36	10	0.50	12	8	5	137.61	158.79	364.32	12.994
NF-3-EDA-GLA/E2	35	10	0.48	12	8	5	134.91	158.79	361.09	12.872
PF-3-EDA-GLA/E3	36	10	0.50	12	8	4	139.81	150.00	375.18	13.200
OF-3-EDA-GLA/E4	38	10	0.52	11	9	4	144.19	159.23	384.28	13.479
LF-3-EDA-GLA/E5	38	10	0.52	11	9	4	144.19	159.23	384.28	13.4898
MF-3-EDA-GLA/E6	41	10	0.57	13	9	5	156.40	168.02	415.69	14.228
Etoposide	42	12	0.55	5	13	3	139.11	160.83	389.66	9.322

* predicted with FORECASTER platform.

**Table 4:** Lipophilicity–partition coefficients.

Compound/ID	* iLOGP	* XLOGP	* WLOGP	* MLOGP	* Silicos-IT LogP	* Consensus LogP	LogD at pI (logP score)	LogD at pH 7.4 (logD score)	HLB
CF-3-GLA/A1	1.67	−1.85	0.53	0.34	1.39	0.42	−2.04	−4.49	19.63
NF-3-GLA/A2	1.75	−1.80	0.28	0.12	1.34	0.34	−2.15	−4.59	20.09
PF-3-GLA/A3	2.30	−0.50	0.62	0.34	1.30	0.81	−1.96	−4.66	19.87
OF-3-GLA/A4	2.33	−1.16	0.55	0.06	1.00	0.56	−2.18	−5.12	20.68
LF-3-GLA/A5	2.14	−1.16	0.55	0.06	1.00	0.52	−2.18	−5.12	20.68
MF-3-GLA/A6	2.10	−0.16	1.32	0.68	1.78	1.14	−1.63	−4.17	19.14
CF-7-GLA/B1	2.33	−2.17	0.61	−1.88	0.87	−0.05	−1.81	−2.98	19.63
NF-7-GLA/B2	1.77	−2.12	0.36	−2.09	0.83	−0.25	−1.92	−3.07	20.09
MF-7-GLA/B6	2.64	−0.49	1.40	−1.54	1.27	0.65	−1.40	−2.63	19.14
CF-3-GLA-HA/C1	1.10	−2.58	−0.05	−0.02	0.62	−0.19	−2.62	−2.64	25.22
NF-3-GLA-HA/C2	2.19	−2.53	−0.30	−0.23	0.58	−0.06	−2.73	−2.75	25.68
PF-3-GLA-HA/C3	2.21	−1.23	0.04	−0.02	0.54	0.31	−2.55	−2.82	25.45
OF-3-GLA-HA/C4	2.84	−1.89	−0.03	−0.29	0.23	0.17	−2.79	−3.20	26.25
LF-3-GLA-HA/C5	2.39	−1.89	−0.03	−0.29	0.23	0.08	−2.79	−3.20	26.25
MF-3-GLA-HA/C6	2.09	−0.90	0.74	0.33	1.01	0.65	−2.21	−2.21	24.72
CF-3-EA-GLA/D1	2.70	−2.46	−0.05	−2.21	1.51	−0.10	−2.49	−3.52	25.07
NF-3-EA-GLA/D2	2.01	−2.41	−0.31	−2.42	1.47	−0.33	−2.60	−3.63	25.52
PF-3-EA-GLA/D3	2.11	−1.11	0.04	−2.21	1.44	0.05	−2.07	−2.23	25.28
OF-3-EA-GLA/D4	3.03	−1.77	−0.03	−2.50	1.12	−0.03	−2.30	−2.40	26.06
LF-3-EA-GLA/D5	3.31	−1.77	−0.03	−2.50	1.12	0.03	−2.30	−2.40	26.06
MF-3-EA-GLA/D6	3.03	−0.77	0.73	−1.90	1.91	0.60	−2.34	−3.68	24.56
CF-3-EDA-GLA/E1	2.51	−3.03	−0.48	−2.62	1.19	−0.49	−3.25	−4.23	29.26
NF-3-EDA-GLA/E2	1.86	−2.98	−0.73	−2.83	1.16	−0.71	−3.36	−4.34	29.71
PF-3-EDA-GLA/E3	2.30	−1.68	−0.39	−2.62	1.12	−0.26	−2.80	−2.96	29.48
OF-3-EDA-GLA/E4	2.92	−2.34	−0.46	−2.90	0.81	0.39	−3.03	−3.13	30.26
LF-3-EDA-GLA/E5	2.36	−2.34	−0.46	−2.90	0.81	−0.51	−3.03	−3.13	30.26
MF-3-EDA-GLA/E6	2.14	−1.35	0.31	−2.30	1.59	0.08	−3.04	−4.33	28.76
Etoposide	3.31	0.6	1.01	−0.14	0.95	1.15	1.16	1.16	14.79

* predicted with SwissADME. The unmarked ones were predicted using MarvinSketch. iLOGP: in-house physics-based method implemented by Daina A. et al. (2014) [74]. This method is based on the solvation-free energies in n-octanol and water, which were calculated according to the *generalised Born and solvent-accessible surface area* model. XLOGP3: an atomistic method (calculated with the XLOGP programme, v 3.2.2, CCBG, Shanghai Institute of Organic Chemistry). This method includes correction factors and an information library [74,75,76,77]. WLOGP: an atomistic method based on the fragmentation system by Wildman şi Crippen (1999) [78]. MLOGP: a topological method implemented by Moriguchi I. et al. (1992) [79], Moriguchi I. et al. (1994) [80], and Lipinski PA. et al. A. et al. (1997) [81]. It is based on a linear relationship with 13 molecular descriptors. SILICOS-IT: a hybrid method based on 27 fragments and 7 topological descriptors (calculated with FILTER-IT, v 1.0.2, SILICOS-IT). Consensus: represents the average of the values calculated by the five proposed methods [74].

**Table 5:** Permeability and interactions with P-gp (SwissADME).

Compound/ID	GI Absorption	BBB Permeant	* CNS MPO	P-gp Substrate	log Kp (cm/s)
CF-3-GLA/A1	High	No	2.94	Yes	−10.42
NF-3-GLA/A2	High	No	3.03	Yes	−10.31
PF-3-GLA/A3	High	No	3.52	Yes	−9.48
OF-3-GLA/A4	High	No	3.32	Yes	−10.12
LF-3-GLA/A5	High	No	3.32	Yes	−10.12
MF-3-GLA/A6	Low	No	2.28	Yes	−9.65
CF-7-GLA/B1	Low	No	2.63	Yes	−10.65
NF-7-GLA/B2	Low	No	2.71	No	−10.54
MF-7-GLA/B6	Low	No	2.34	Yes	−9.88
CF-3-GLA-HA/C1	Low	No	2.87	No	−11.03
NF-3-GLA-HA/C2	Low	No	2.52	No	−10.92
PF-3-GLA-HA/C3	Low	No	3.16	Yes	−10.09
OF-3-GLA-HA/C4	Low	No	3.00	Yes	−10.73
LF-3-GLA-HA/C5	Low	No	3.00	Yes	−10.73
MF-3-GLA-HA/C6	Low	No	2.14	No	−10.27
CF-3-EA-GLA/D1	Low	No	2.21	Yes	−11.12
NF-3-EA-GLA/D2	Low	No	2.27	Yes	−11.01
PF-3-EA-GLA/D3	Low	No	2.24	Yes	−10.17
OF-3-EA-GLA/D4	Low	No	2.24	Yes	−10.81
LF-3-EA-GLA/D5	Low	No	2.24	Yes	−10.81
MF-3-EA-GLA/D6	Low	No	2.11	Yes	−10.35
CF-3-EDA-GLA/E1	Low	No	2.30	No	−11.52
NF-3-EDA-GLA/E2	Low	No	2.37	Yes	−11.41
PF-3-EDA-GLA/E3	Low	No	2.34	Yes	−10.57
OF-3-EDA-GLA/E4	Low	No	2.34	Yes	−11.21
LF-3-EDA-GLA/E5	Low	No	2.34	Yes	−11.21
MF-3-EDA-GLA/E6	Low	No	2.16	No	−10.75
Etoposide	Low	No	3.25	Yes	−9.46

* CNS MPO score was assessed with MarvinSketch. Log Kp indicates skin permeation and was calculated according to Potts R. O. et al. (1992) [82].

**Table 6:** The number of broken rules, according to Lipinski, Ghose, Veber, Egan, and Muegge and the bioavailability score, the lead-likeness score, the synthetic accessibility score (SwissADME), and the drug-likeness score (Osiris Property Explorer).

Compound/ID	Lipinski	Ghose	Veber	Egan	Muegge	BD Score	Lead-Likeness	Drug-Likeness	SA
CF-3-GLA/A1	0	0	1	1	0	0.56	2	−7.32	3.65
NF-3-GLA/A2	0	0	1	1	0	0.56	2	−6.89	3.61
PF-3-GLA/A3	0	0	0	1	0	0.56	2	−3.95	3.72
OF-3-GLA/A4	1	2	1	1	0	0.56	2	−3.86	4.49
LF-3-GLA/A5	1	2	1	1	0	0.56	2	−3.86	4.49
MF-3-GLA/A6	2	2	1	1	1	0.11	2	−7.00	4.75
CF-7-GLA/B1	0	0	1	1	1	0.56	2	−13.14	3.57
NF-7-GLA/B2	0	0	1	1	1	0.56	2	−12.74	3.53
MF-7-GLA/B6	2	2	1	1	1	0.11	2	−15.22	4.70
CF-3-GLA-HA/C1	1	0	1	1	2	0.55	2	−7.42	3.79
NF-3-GLA-HA/C2	1	0	1	1	2	0.55	2	−6.99	3.76
PF-3-GLA-HA/C3	1	0	1	1	0	0.55	2	−4.01	3.87
OF-3-GLA-HA/C4	2	2	1	1	1	0.17	2	−3.93	4.59
LF-3-GLA-HA/C5	2	2	1	1	1	0.17	2	−3.93	4.59
MF-3-GLA-HA/C6	2	3	2	1	1	0.17	2	−7.09	4.86
CF-3-EA-GLA/D1	2	2	2	1	2	0.17	2	−16.42	4.05
NF-3-EA-GLA/D2	1	2	2	1	2	0.55	2	−16.14	4.00
PF-3-EA-GLA/D3	2	2	2	1	0	0.17	2	−13.22	4.12
OF-3-EA-GLA/D4	2	2	2	1	1	0.17	2	−13.03	4.84
LF-3-EA-GLA/D5	2	2	2	1	1	0.17	2	−13.03	4.84
MF-3-EA-GLA/D6	2	3	2	1	1	0.17	2	−16.13	5.12
CF-3-EDA-GLA/E1	2	3	2	1	2	0.17	2	−13.65	3.98
NF-3-EDA-GLA/E2	1	3	2	1	2	0.55	2	−0.67	3.94
PF-3-EDA-GLA/E3	2	2	2	1	1	0.17	2	−9.98	4.06
OF-3-EDA-GLA/E4	2	4	2	1	2	0.17	2	−9.88	4.83
LF-3-EDA-GLA/E5	2	4	2	1	2	0.17	2	−9.88	4.83
MF-3-EDA-GLA/E6	2	3	2	1	1	0.17	2	−12.88	5.09
Etoposide	2	3	1	1	2	0.17	1	−0.28	6.27

Lead-likeness criteria established according to Simon J. Teague et al. (1999) [83]: 250 ≤ MW ≤ 350; XlogP ≤ 3.5; RB ≤ 7.

**Table 7:** The bioactivity assessed using Molinspiration. The classification of the hybrids and etoposide according to the topoisomerase II inhibitory activity (Pa > Pi in each case) assessed with the PASS Online platform.

Compound/ID	GPCR Ligand	Ion Channel Modulator	Kinase Inhibitor	Nuclear Receptor Ligand	Protease Inhibitor	Enzyme Inhibitor	Topoisomerase II Inhibitory Activity (PASS Online)	
							Pa	Pi
CF-3-GLA/A1	0.23 *	−0.03	−0.03	−0.20	0.07	0.32 *	0.413	0.006
NF-3-GLA/A2	0.29 *	−0.06	0.03	−0.08	0.05	0.24 *	0.304	0.010
PF-3-GA/A3	0.29 *	−0.06	0.04	−0.12	0.03	0.22 *	0.296	0.011
OF-3-GLA/A4	0.3 *	−0.12	−0.03	−0.18	0.03	0.36 *	0.492	0.004
LF-3-GLA/A5	0.3 *	−0.12	−0.03	−0.18	0.03	0.36 *	0.492	0.004
MF-3-GLA/A6	0.26 *	−0.10	−0.14	−0.29	0.10	0.47 *	**0.517**	0.004
CF-7-GLA/B1	0.24 *	0.06	−0.12	−0.19	0.10	0.35 *	0.505	0.004
NF-7-GLA/B2	0.31 *	0.04	−0.06	−0.07	0.08	0.27 *	0.405	0.006
MF-7-GLA/B6	0.27 *	−0.06	−0.20	−0.28	0.07	0.49 *	**0.580**	0.004
CF-3-GA-HA/C1	0.25 *	−0.15	0.09	−0.28	0.42 *	0.54 **	0.333	0.009
NF-3-GLA-HA/C2	0.32 *	−0.18	0.15	−0.17	0.41 *	0.47 *	0.226	0.016
PF-3-GLA-HA/C3	0.32 *	−0.18	0.16	−0.20	0.37 *	0.44 *	0.218	0.017
OF-3-GLA-HA/C4	0.32 *	−0.25	0.08	−0.25	0.35 *	0.57 **	0.420	0.005
LF-3-GLA-HA/C5	0.32 *	−0.25	0.08	−0.25	0.35 *	0.57 **	0.420	0.005
MF-3-GLA-HA/C6	0.28 *	−0.25	−0.06	−0.38	0.40 *	0.63 **	0.448	0.005
CF-3-EA-GLA/D1	0.21 *	−0.02	0.07	−0.29	0.11	0.32 *	0.370	0.007
NF-3-EA-GLA/D2	0.27 *	−0.02	0.12	−0.18	0.09	0.25 *	0.279	0.012
PF-3-EA-GLA/D3	0.27 *	−0.05	0.13	−0.21	0.06	0.23 *	0.272	0.013
OF-3-EA-GLA/D4	0.28 *	−0.16	0.06	−0.28	0.06	0.35 *	0.455	0.005
LF-3-EA-GLA/D5	0.28 *	−0.16	0.06	−0.28	0.06	0.35 *	0.455	0.005
MF-3-EA-GLA/D6	0.21 *	−0.25	−0.14	−0.47	0.12	0.36 *	0.478	0.005
CF-3-EDA-GLA/E1	0.23 *	−0.03	0.01	−0.32	0.14	0.31 *	0.391	0.007
NF-3-EDA-GLA/E2	0.29 *	−0.03	0.07	−0.22	0.12	0.24 *	0.295	0.011
PF-3-EDA-GLA/E3	0.30 *	−0.04	0.09	−0.25	0.11	0.22 *	0.288	0.011
OF-3-EDA-GLA/E4	0.31 *	−0.16	0.02	−0.31	0.10	0.34 *	0.466	0.005
LF-3-EDA-GLA/E5	0.31 *	−0.16	0.02	−0.31	0.10	0.34 *	0.466	0.005
MF-3-EDA-GLA/E6	0.24 *	−0.25	−0.18	−0.50	0.16	0.35 *	0.492	0.004
Etoposide	0.18	−0.48	−0.38	−0.33	0.12	0.30 *	**0.945**	0.002

* Values equal to or higher than 0.2. ** Values equal to or higher than 0.5. The first three highest Pa values (including etoposide) are in bold.

**Table 8:** The cancer cell lines on which the hybrids exhibit cytotoxicity and the number of hybrids that have anti-cancer effects on each cell line (from the highest to the lowest), according to the CLC-Pred platform (when Pa > 0.4).

No.	Cell Line Name	Cell Line Code	The Number of Hybrids Active on the Cancer Cell Line (Out of All 27)
1.	Adult acute myeloid leukaemia	OCI-AML2	23
2.	Bladder carcinoma	UMUC3	23
3.	Promyeloblast leukaemia	HL-60	22
4.	Plasma cell myeloma	OPM-2	18
5.	Pancreas Adenocarcinoma	CAPAN-1	17
6.	Adult acute monocytic leukaemia	MONO-MAC-6	12
7.	Cervical squamous cell carcinoma	SiHa	11
8.	Gastrointestinal stromal tumour	GIST882	6
9.	Gastrointestinal stromal tumour	GIST48	6
10.	Gastric carcinoma	SNU-5	5
11.	Acute myeloblastic leukaemia	Kasumi-1	5
12.	Pancreatic adenocarcinoma	HuP-T3	5
13.	Papillary adenocarcinoma	NCI-H441	4
14.	Barrett adenocarcinoma	OE33	3
15.	Lung adenocarcinoma	SK-LU-1	3
16.	Pancreatic carcinoma	YAPC	3
17.	Prostate carcinoma	PC-3	3
18.	Colorectal adenocarcinoma	SW48	3
19.	Metastatic melanoma	SK-MEL-1	2
20.	Breast carcinoma	HCC1937	2
21.	Urinary bladder carcinoma	HT1197	1

**Table 9:** The frequency of most probable toxic/adverse effects predicted by the PASS Online platform (Pa > Pi) from the highest to the lowest.

No.	Toxic/Adverse Effect	The Number of Hybrids Which Have a Probability of Presenting the Toxic/Adverse Effect (Out of All 27)
1.	Allergic contact dermatitis	23
2.	Stomatitis	19
3.	Photoallergy dermatitis	10
4.	Myoclonus	9
5.	Necrosis	9
6.	Nail discoloration	9
7.	Asthma	9
8.	Bullous pemphigoid	8
9.	Urinary retention	8
10.	Delirium	7
11.	Bradycardia	7
12.	Hypotonia	4
13.	Hyperglycaemia	4
14.	Hypotonia	4
15.	Psychoses	3
16.	Ataxia	2
17.	Xerostomia	2
18.	Hypertension	2
19.	Haematuria	1
20.	Thrombophlebitis	1
21.	Torsades de pointes	1
22.	Diarrhoea	1
23.	Vascular toxicity	1

**Table 10:** The docking results of the hybrids to the human topoisomerase II beta-DNA complex (3QX3 PDB code) in the same binding site and interaction sites as etoposide (from highest to lowest Fitted Score) obtained with the FORECASTER platform.

No.	Compound Name	Internal Energy (kcal/mol)	Rank Score	Match Score	Fitted Score
1.	OF-3-EDA-GLA/E4	−130.7	−26.6	36.4	**−32.4**
2.	MF-7-GLA/B6	−83.2	−25.7	27.5	**−30.1**
3.	NF-3-EA-GLA/D2	−97.5	−24.5	31.6	**−29.6**
4.	NF-7-GLA/B2	−95.4	−24.6	29.8	**−29.4**
5.	CF-3-GLA-HA/C1	−122.1	−24.3	27.5	**−28.7**
6.	CF-3-GLA/A1	−101.7	−25.0	21.1	**−28.4**
7.	NF-3-GLA/A2	−102.5	−24.2	20.9	**−27.6**
8.	OF-3-GLA-HA/C4	−118.3	−22.5	29.6	−27.2
9.	NF-3-EDA-GLA/E2	−126.2	−23.8	20.8	−27.1
10.	LF-3-EDA-GLA/E5	−133.8	−24.0	18.5	−27.0
11.	LF-3-GLA/A5	−99.4	−23.9	18.6	−26.9
12.	MF-3-EDA-GLA/E6	−110.7	−20.7	29.0	−25.3
13.	OF-3-EA-GLA/D4	−106.2	−21.4	18.6	−24.4
14.	MF-3-GLA-HA/C6	−103.8	−20.5	23.1	−24.2
15.	PF-3-EDA-GLA/E3	−121.1	−21.2	18.2	−24.1
16.	PF-3-EA-GLA/D3	−97.0	−20.1	24.6	−24.0
17.	NF-3-GLA-HA/C2	−112.9	−20.5	20.0	−23.7
18.	CF-3-EA-GLA/D1	−104.4	−20.4	18.3	−23.3
19.	CF-3-EDA-GLA/E1	−125.1	−19.6	18.7	−22.6
20.	PF-3-GLA-HA/C3	−121.4	−19.5	17.6	−22.4
21.	LF-3-EA-GLA/D5	−97.9	−18.6	24.1	−22.4
22.	PF-3-GLA/A3	−93.9	−19.6	15.8	−22.1
23.	CF-7-GLA/B1	−91.0	−19.1	18.8	−22.1
24.	OF-3-GLA/A4	−99.0	−18.7	17.7	−21.6
25.	MF-3-GLA/A6	−84.0	−18.1	18.4	−21.1
26.	LF-3-GLA-HA/C5	−110.2	−17.5	17.4	−20.3
27.	MF-3-EA-GLA/D6	−90.1	−16.8	19.1	−19.9

The top 25% of the results are highlighted (in bold), indicating an increased affinity for the target.

**Table 11:** The results of the molecular docking study using AutoDock Vina and AutoDock, presented as the binding affinity of the compounds to the binding site, expressed as the variation in the Gibbs free energy (ΔG).

Compound	AutoDock Vina	AutoDock							
	ΔG	Top ΔG	Cluster with the Top Binding		Intermolecular Energy			Total Internal Energy	Torsional Free Energy
			Mean ΔG	N.C.	Total	vdW + HB + D	El		
CF-3-GLA/A1	−10.0	**−12.65**	**−11.39**	18	−15.64	−10.58	−5.05	−1.02	2.98
NF-3-GLA/A2	−9.4	−10.85	−9.99	4	−13.84	−8.60	−5.24	−1.05	2.98
PF-3-GLA/A3	−9.2	−9.94	−9.25	10	−12.92	−9.06	−3.86	−2.88	2.98
OF-3-GLA/A4	−9.8	−10.20	−9.53	4	−12.88	−7.54	−5.34	−2.19	2.68
LF-3-GLA/A5	**−10.2**	−10.90	−9.51	9	−13.59	−8.75	−4.84	−1.95	2.68
MF-3-GLA/A6	**−10.5**	−9.78	−9.03	7	−13.06	−10.58	−2.48	−1.80	3.28
CF-7-GLA/B1	−9.5	**−12.73**	**−10.73**	4	−15.71	−8.43	−7.28	−2.67	2.98
NF-7-GLA/B2	−8.3	**−11.97**	**−10.89**	8	−14.95	−7.41	−7.54	−2.59	2.98
MF-7-GLA/B6	**−10.3**	**−13.58**	**−12.83**	10	−16.86	−9.57	−7.29	−2.66	3.28
CF-3-GLA-HA/C1	−9.8	−10.28	−9.84	2	−13.26	−7.52	−4.43	−2.70	2.98
NF-3-GLA-HA/C2	**−10.7**	−9.61	−8.56	7	−12.6	−11.64	−0.96	−2.82	2.98
PF-3-GLA-HA/C3	−10.1	−9.20	−8.58	2	−12.18	−11.70	−0.48	−3.26	2.98
OF-3-GLA-HA/C4	**−10.6**	−10.75	−9.02	7	−13.43	−12.29	−1.14	−2.29	2.68
LF-3-GLA-HA/C5	−9.6	−9.15	−8.34	4	−11.84	−9.93	−1.91	−3.37	2.68
MF-3-GLA-HA/C6	−8.9	−7.68	−7.53	2	−10.96	−11.01	0.05	−3.11	3.28
CF-3-EA-GLA/D1	−9.3	−11.01	−8.14	2	−14.88	−6.96	−5.19	−3.14	3.88
NF-3-EA-GLA/D2	−10.0	−10.59	−9.24	4	−14.46	−7.65	−6.82	−2.33	3.88
PF-3-EA-GLA/D3	−10.0	−10.80	−10.27	2	−14.68	−8.41	−6.27	−3.45	3.88
OF-3-EA-GLA/D4	**−10.6**	**−12.08**	−10.46	13	−15.66	−8.86	−6.84	−3.21	3.58
LF-3-EA-GLA/D5	**−10.2**	**−11.52**	**−11.52**	1	−15.09	−7.69	−7.41	−2.6	3.58
MF-3-EA-GLA/D6	−9.7	−10.81	−10.22	3	−14.99	−8.97	−6.02	−3.36	4.18
CF-3-EDA-GLA/E1	**−10.2**	−10.90	−9.44	8	−14.48	−8.22	−6.26	−1.94	3.58
NF-3-EDA-GLA/E2	−9.6	−7.82	−7.82	1	−11.40	−11.51	0.11	−3.75	3.58
PF-3-EDA-GLA/E3	−9.8	−9.98	−8.98	2	−13.56	−7.52	−6.04	−3.00	3.58
OF-3-EDA-GLA/E4	**−10.4**	**−12.01**	**−11.61**	8	−15.29	−10.29	−5.00	−3.28	3.28
LF-3-EDA-GLA/E5	−10.0	−11.14	**−11.14**	1	−14.42	−9.06	−5.36	−2.16	3.28
MF-3-EDA-GLA/E6	−9.6	−9.84	−7.95	6	−13.72	−11.82	−1.90	−3.39	3.88

N.C. = number of conformations; vdW = van der Waals; HB = hydrogen bonding; D = desolvation; El = electrostatic. The top 25% of the results are highlighted (in bold), indicating an increased affinity for the target. The energies are presented as kcal/mol.

**Table 12:** The classification of the hybrids in each class and the ID attributed to each hybrid.

Hybrid Class/Hybrid Class ID				
FQ-3-GLA/ A	FQ-7-GLA/ B	FQ-3-GLA-HA/ C	FQ-3-EA-GLA/ D	FQ-3-EDA-GLA/ E
Hybrid/Hybrid ID				
CF-3-GLA/ A1	CF-7-GLA/ B1	CF-3-GLA-HA/ C1	CF-3-EA-GLA/ D1	CF-3-EDA-GLA/ E1
NF-3-GLA/ A2	NF-7-GLA/ B2	NF-3-GLA-HA/ C2	NF-3-EA-GLA/ D2	NF-3-EDA-GLA/ E2
PF-3-GLA/ A3	-	PF-3-GLA-HA/ C3	PF-3-EA-GLA/ D3	PF-3-EDA-GLA/ E3
OF-3-GLA/ A4	-	OF-3-GLA-HA/ C4	OF-3-EA-GLA/ D4	OF-3-EDA-GLA/ E4
LF-3-GLA/ A5	-	LF-3-GLA-HA/ C5	LF-3-EA-GLA/ D5	LF-3-EDA-GLA/ E5
MF-3-GLA/ A6	MF-7-GLA/ B6	MF-3-GLA-HA/ C6	MF-3-EA-GLA/ D6	MF-3-EDA-GLA/ E6

A corresponds to class FQ-3-GLA (hybrids A1-A6); B corresponds to class FQ-7-GLA (hybrids B1, B2, B6); C corresponds to class FQ-3-GLA-HA (hybrids C1-C6); D corresponds to class FQ-3-EA-GLA (hybrids D1-D6); E corresponds to class FQ-3-EDA-GLA (hybrids E1–E6).

## Data Availability

The original contributions presented in the study are included in the article/Supplementary Materials, further inquiries can be directed to the corresponding author.

## Supplementary Materials

The following supporting information can be downloaded at https://www.mdpi.com/article/10.3390/ph17121593/s1: Table S1—FQs as anti-cancer agents; Table S2—FQs hybrids with different compounds and their biological effects; Table S3—the structure–activity relationship of the FQ is detailed for each position of the quinolone nucleus; Table S4—the IUPAC name, SMILES string, InChI string and InChI key of the hybrids and etoposide; Table S5–water solubility; Figure S1—the characteristics of Lipinski, Ghose, Veber, Egan, and Muegge drug-likeness rules according to SwissADME; Table S6—prediction of pKa score, pI, molar polarizability, and the best conformation for each hybrid (MarvinSketch); Table S7—mechanisms of action and adverse/toxic effects, probability to be active (Pa) > probability to be inactive (Pi) (PASS Online platform); Table S8—anti-cancer effect: most probable cell lines for which compounds exhibit cytotoxicity, (Pa > 0.4) (CLC-Pred platform); Table S9—metabolic pathways (Toxtree); Table S10—compound metabolism assessed using SmartCyp; Table S11—acute toxicity in rodents when administered intraperitoneally, intravenously, orally, and subcutaneously: LD50 in mg/kg; Table S12—acute toxicity in rodents. Classification of chemicals by the OECD Project; Table S13—toxicity according to Cramer’s rule, Kroes TTC, and the Verhaar scheme (Toxtree programme); Table S14—carcinogenic (genotoxic and non-genotoxic) and mutagenic effects evaluated using Toxtree and Osiris Property Explorer; Table S15—irritant/corrosive effect on the skin and eyes, effect on the reproductive system, biodegradability, protein and DNA binding alerts, assessed using Toxtree and Osiris Property Explorer; Figure S2—the conformations of hybrids A1–A6 (from class FQ-3-GLA) in the binding site (a–f) and their interaction with the enzyme–DNA complex (a’–f’); Figure S3—the conformations of hybrids B1, B2, and B6 (from class FQ-7-GLA) in the binding site (a–c) and their interaction with the enzyme–DNA complex (a’–c’); Figure S4—the conformations of hybrids C1–C6 (from class FQ-3-GLA-HA) in the binding site (a–f) and their interaction with the enzyme–DNA complex (a’–f’); Figure S5—the conformations of hybrids D1–D6 (from class FQ-3-EA-GLA) in the binding site (a–f) and their interaction with the enzyme–DNA complex (a’–f’); Figure S6—the conformations of hybrids E1–E6 (from class FQ-3-EDA-GLA) in the binding site (a–f) and their interaction with the enzyme–DNA complex (a’–f’); Figure S7—synthesis proposal for hybrid OF-3-EDA-GLA; Figure S8—synthesis proposal for hybrids FQ-3-GLA-HA.

## Abbreviations

ACN	Acetonitrile
ADME	Absorption, distribution, metabolism, and excretion
HAA	Heavy aromatic atoms
HB	Hydrogen bonding
BBB	Blood–brain barrier
BD	Bioavailability
CLC-Pred	Cell line cytotoxicity predictor
CF	Ciprofloxacin
CNS MPO	Central Nervous System Multiparameter Optimization
D	Desolvation
DMF	Dimethylformamide
ECFP	Extended connectivity fingerprint
El	Electrostatic
FQs	Fluoroquinolones
GLA	Glutamic acid
GPCR	G-protein-coupled receptor
GUSAR	General unrestricted Structure–activity relationships
HA	Heavy atoms
HLB	Hydrophilic lipophilic balance
LD50	Lethal dose 50
LF	Levofloxacin
MACCS	Molecular access system
MF	Moxifloxacin
MR	Molar refractivity
MW	Molecular weight
N.C.	Number of conformations
NF	Norfloxacin
OECD	Organisation for Economic Cooperation and Development
OF	Ofloxacin
Pa	Probability to be active
PASS	Prediction of Activity Spectrafor Substances
PDB	Protein Data Bank
PF	Pefloxacin
P-gp	P-glycoprotein
Pi	Probability to be inactive
Py	Pyridine
RB	Rotatable bonds
RMSD	Root mean square deviation
SA	Synthetic accessibility
Tc	Tanimoto coefficient
TPSA	Topological polar surface area
TTC	Threshold of toxicological concern
vdW	van der Waals

## References

[1] Yadav V., Talwar P. (2019). Repositioning of Fluoroquinolones from Antibiotic to Anti-Cancer Agents: An Underestimated Truth. Biomed. Pharmacother..

[2] Aranha O., Grignon R., Fernandes N., McDonnell T., Wood D., Sarkar F. (2003). Suppression of Human Prostate Cancer Cell Growth by Ciprofloxacin Is Associated with Cell Cycle Arrest and Apoptosis. Int. J. Oncol..

[3] Aranha O., Wood D.P., Sarkar F.H. (2000). Ciprofloxacin Mediated Cell Growth Inhibition, S/G2-M Cell Cycle Arrest, and Apoptosis in a Human Transitional Cell Carcinoma of the Bladder Cell Line. Clin. Cancer Res..

[4] Perucca P., Savio M., Cazzalini O., Mocchi R., Maccario C., Sommatis S., Ferraro D., Pizzala R., Pretali L., Fasani E. (2014). Structure–Activity Relationship and Role of Oxygen in the Potential Antitumour Activity of Fluoroquinolones in Human Epithelial Cancer Cells. J. Photochem. Photobiol. B Biol..

[5] Morgan D.O. (1995). Principles of CDK Regulation. Nature.

[6] Peinado H., Portillo F., Cano A. (2004). Transcriptional Regulation of Cadherins during Development and Carcinogenesis. Int. J. Dev. Biol..

[7] Iwatsuki M., Mimori K., Yokobori T., Ishi H., Beppu T., Nakamori S., Baba H., Mori M. (2010). Epithelial-Mesenchymal Transition in Cancer Development and Its Clinical Significance. Cancer Sci..

[8] Gong J.-H., Liu X.-J., Shang B.-Y., Chen S.-Z., Zhen Y.-S. (2010). HERG K+ Channel Related Chemosensitivity to Sparfloxacin in Colon Cancer Cells. Oncol. Rep..

[9] Chen T.-C., Hsu Y.-L., Tsai Y.-C., Chang Y.-W., Kuo P.-L., Chen Y.-H. (2014). Gemifloxacin Inhibits Migration and Invasion and Induces Mesenchymal–Epithelial Transition in Human Breast Adenocarcinoma Cells. J. Mol. Med..

[10] AbuBaih R., Fawzy M., Nazmy M. (2023). The Prospective Potential of Fluoroquinolones as Anticancer Agents. J. Mod. Res..

[11] Shan G., Li Y., Zhang J., Li W., Szulwach K.E., Duan R., Faghihi M.A., Khalil A.M., Lu L., Paroo Z. (2008). A Small Molecule Enhances RNA Interference and Promotes MicroRNA Processing. Nat. Biotechnol..

[12] Valianatos G., Valcikova B., Growkova K., Verlande A., Mlcochova J., Radova L., Stetkova M., Vyhnakova M., Slaby O., Uldrijan S. (2017). A Small Molecule Drug Promoting MiRNA Processing Induces Alternative Splicing of MdmX Transcript and Rescues P53 Activity in Human Cancer Cells Overexpressing MdmX Protein. PLoS ONE.

[13] Sissi C., Palumbo M. (2003). The Quinolone Family: From Antibacterial to Anticancer Agents. CMCACA.

[14] Bourikas L.A., Kolios G., Valatas V., Notas G., Drygiannakis I., Pelagiadis I., Manousou P., Klironomos S., Mouzas I.A., Kouroumalis E. (2009). Ciprofloxacin Decreases Survival in HT-29 Cells via the Induction of TGF-Β1 Secretion and Enhances the Anti-Proliferative Effect of 5-Fluorouracil. Br. J. Pharmacol..

[15] Herold C., Ocker M., Ganslmayer M., Gerauer H., Hahn E.G., Schuppan D. (2002). Ciprofloxacin Induces Apoptosis and Inhibits Proliferation of Human Colorectal Carcinoma Cells. Br. J. Cancer.

[16] Sun J., Shi Z., Liu S., Kang Y., Hu G., Huangfu C., Deng J., Liu B. (2013). Trimethoxy-Benzaldehyde Levofloxacin Hydrazone Inducing the Growth Arrest and Apoptosis of Human Hepatocarcinoma Cells. Cancer Cell Int..

[17] Abdel-Aal M.A.A., Abdel-Aziz S.A., Shaykoon M.S.A., Abuo-Rahma G.E.A. (2019). Towards Anticancer Fluoroquinolones: A Review Article. Arch. Pharm. Chem. Life Sci..

[18] Herrero-Ruiz A., Martínez-García P.M., Terrón-Bautista J., Millán-Zambrano G., Lieberman J.A., Jimeno-González S., Cortés-Ledesma F. (2021). Topoisomerase IIα Represses Transcription by Enforcing Promoter-Proximal Pausing. Cell Rep..

[19] Matias-Barrios V.M., Dong X. (2023). The Implication of Topoisomerase II Inhibitors in Synthetic Lethality for Cancer Therapy. Pharmaceuticals.

[20] Swedan H.K., Kassab A.E., Gedawy E.M., Elmeligie S.E. (2023). Design, Synthesis, and Biological Evaluation of Novel Ciprofloxacin Derivatives as Potential Anticancer Agents Targeting Topoisomerase II Enzyme. J. Enzym. Inhib. Med. Chem..

[21] Swedan H.K., Kassab A.E., Gedawy E.M., Elmeligie S.E. (2023). Topoisomerase II Inhibitors Design: Early Studies and New Perspectives. Bioorg. Chem..

[22] Gao Y., Shang Q., Li W., Guo W., Stojadinovic A., Mannion C., Man Y., Chen T. (2020). Antibiotics for Cancer Treatment: A Double-Edged Sword. J. Cancer.

[23] Elanany M.A., Osman E.E.A., Gedawy E.M., Abou-Seri S.M. (2023). Design and Synthesis of Novel Cytotoxic Fluoroquinolone Analogs through Topoisomerase Inhibition, Cell Cycle Arrest, and Apoptosis. Sci. Rep..

[24] Żabka A., Winnicki K., Polit J.T., Maszewski J. (2015). The Effects of Anti-DNA Topoisomerase II Drugs, Etoposide and Ellipticine, Are Modified in Root Meristem Cells of Allium Cepa by MG132, an Inhibitor of 26S Proteasomes. Plant Physiol. Biochem..

[25] DrugBank Online Mitoxantrone. https://go.drugbank.com/drugs/DB01204.

[26] Christowitz C., Davis T., Isaacs A., Van Niekerk G., Hattingh S., Engelbrecht A.-M. (2019). Mechanisms of Doxorubicin-Induced Drug Resistance and Drug Resistant Tumour Growth in a Murine Breast Tumour Model. BMC Cancer.

[27] Thorn C.F., Oshiro C., Marsh S., Hernandez-Boussard T., McLeod H., Klein T.E., Altman R.B. (2011). Doxorubicin Pathways: Pharmacodynamics and Adverse Effects. Pharmacogenet. Genom..

[28] Khasraw M., Bell R., Dang C. (2012). Epirubicin: Is It like Doxorubicin in Breast Cancer? A Clinical Review. Breast.

[29] Shan B.-J., Shen X.-B., Jin W., Dong M.-H., Han X.-H., Lin L., Chen J., Huang D.-B., Qian J., Zhang J.-J. (2020). Standard-Dose Epirubicin Increases the Pathological Complete Response Rate in Neoadjuvant Chemotherapy for Breast Cancer: A Multicenter Retrospective Study. Gland Surg..

[30] Qashou E., Al-Hiari Y., Kasabri V., AlBashiti R., AlAlawi S., Telfah A., AlHadid A. (2022). Antiproliferative Activities of Lipophililic Fluoroquinolones- Based Scaffold Against a Panel of Solid and Liquid Cancer Cell Lines. Asian Pac. J. Cancer Prev..

[31] Salih M., Al-Hiari Y., Kasabri V., Darwish R., Abumansour H., Bourghli L., Al Alawi S., Albashiti R. (2022). Newly Substituted Anilino-Fluoroquinolones with Proliferation Inhibition Potential against a Panel of Cancer Cell Lines. Asian Pac. J. Cancer Prev..

[32] DrugBank Online Etoposide. https://go.drugbank.com/drugs/DB00773.

[33] DrugBank Online Teniposide. https://go.drugbank.com/drugs/DB00444.

[34] DrugBank Online Amsacrine. https://go.drugbank.com/drugs/DB00276.

[35] Hevener K., Verstak T.A., Lutat K.E., Riggsbee D.L., Mooney J.W. (2018). Recent Developments in Topoisomerase-Targeted Cancer Chemotherapy. Acta Pharm. Sin. B.

[36] Spallarossa A., Lusardi M., Caneva C., Profumo A., Rosano C., Ponassi M. (2021). Bicyclic Basic Merbarone Analogues as Antiproliferative Agents. Molecules.

[37] Sharma P.C., Goyal R., Sharma A., Sharma D., Saini N., Rajak H., Sharma S., Thakur V.K. (2020). Insights on Fluoroquinolones in Cancer Therapy: Chemistry and Recent Developments. Mater. Today Chem..

[38] Abdel-Aziz M., Park S.-E., Abuo-Rahma G.E.-D.A.A., Sayed M.A., Kwon Y. (2013). Novel N-4-Piperazinyl-Ciprofloxacin-Chalcone Hybrids: Synthesis, Physicochemical Properties, Anticancer and Topoisomerase I and II Inhibitory Activity. Eur. J. Med. Chem..

[39] Kloskowski T., Olkowska J., Nazlica A., Drewa T. (2010). The Influence of Ciprofloxacin on Hamster Ovarian Cancer Cell Line CHO AA8. Acta Pol. Pharm..

[40] Fan M., Chen S., Weng Y., Li X., Jiang Y., Wang X., Bie M., An L., Zhang M., Chen B. (2020). Ciprofloxacin Promotes Polarization of CD86+CD206- Macrophages to Suppress Liver Cancer. Oncol. Rep..

[41] Beberok A., Wrześniok D., Minecka A., Rok J., Delijewski M., Rzepka Z., Respondek M., Buszman E. (2018). Ciprofloxacin-Mediated Induction of S-Phase Cell Cycle Arrest and Apoptosis in COLO829 Melanoma Cells. Pharmacol. Rep..

[42] Kloskowski T., Gurtowska N., Olkowska J., Nowak J.M., Adamowicz J., Tworkiewicz J., Dębski R., Grzanka A., Drewa T. (2012). Ciprofloxacin Is a Potential Topoisomerase II Inhibitor for the Treatment of NSCLC. Int. J. Oncol..

[43] Beberok A., Wrześniok D., Rok J., Rzepka Z., Respondek M., Buszman E., Beberok A., Wrześniok D., Rok J., Rzepka Z. (2018). Ciprofloxacin Triggers the Apoptosis of Human Triple-Negative Breast Cancer MDA-MB-231 Cells via the P53/Bax/Bcl-2 Signaling Pathway. Int. J. Oncol..

[44] Zandi A., Zanjani T.M., Ziai S.A., Poul Y.K., Hoseini M.H.M. (2017). Evaluation of the Cytotoxic Effects of Ciprofloxacin on Human Glioblastoma A-172 Cell Line. Middle East J. Cancer.

[45] Yadav V., Varshney P., Sultana S., Yadav J., Saini N. (2015). Moxifloxacin and Ciprofloxacin Induces S-Phase Arrest and Augments Apoptotic Effects of Cisplatin in Human Pancreatic Cancer Cells via ERK Activation. BMC Cancer.

[46] Yadav V., Sultana S., Yadav J., Saini N. (2012). Gatifloxacin Induces S and G2-Phase Cell Cycle Arrest in Pancreatic Cancer Cells via P21/P27/P53. PLoS ONE.

[47] Yu M., Li R., Zhang J. (2016). Repositioning of Antibiotic Levofloxacin as a Mitochondrial Biogenesis Inhibitor to Target Breast Cancer. Biochem. Biophys. Res. Commun..

[48] Kloskowski T., Frąckowiak S., Adamowicz J., Szeliski K., Rasmus M., Drewa T., Pokrywczyńska M. (2022). Quinolones as a Potential Drug in Genitourinary Cancer Treatment—A Literature Review. Front. Oncol..

[49] Kloskowski T., Szeliski K., Fekner Z., Rasmus M., Dąbrowski P., Wolska A., Siedlecka N., Adamowicz J., Drewa T., Pokrywczyńska M. (2021). Ciprofloxacin and Levofloxacin as Potential Drugs in Genitourinary Cancer Treatment—The Effect of Dose–Response on 2D and 3D Cell Cultures. Int. J. Mol. Sci..

[50] Sousa E.J., Graça I., Baptista T., Vieira F.Q., Palmeira C., Henrique R., Jerónimo C. (2013). Enoxacin Inhibits Growth of Prostate Cancer Cells and Effectively Restores MicroRNA Processing. Epigenetics.

[51] Mukherjee P., Mandal E.R., Das S.K. (2005). Evaluation of Antiproliferative Activity of Enoxacin on a Human Breast Cancer Cell Line. Int. J. Hum. Genet..

[52] Mamdooh N., Kasabri V., Al-Hiari Y., Almasri I., Al-Alawi S., Bustanji Y. (2019). Evaluation of Selected Commercial Pharmacotherapeutic Drugs as Potential Pancreatic Lipase Inhibitors and Antiproliferative Compounds. Drug Dev. Res..

[53] ClinicalTrials.gov Study of Vosaroxin or Placebo in Combination with Cytarabine in Patients with First Relapsed or Refractory AML. https://clinicaltrials.gov/study/NCT01191801.

[54] Hernández-López H., Sánchez-Miranda G., Araujo-Huitrado J.G., Granados-López A.J., López J.A., Leyva-Ramos S., Chacón-García L. (2019). Synthesis of Hybrid Fluoroquinolone-Boron Complexes and Their Evaluation in Cervical Cancer Cell Lines. J. Chem..

[55] Jamieson G.C., Fox J.A., Poi M., Strickland S.A. (2016). Molecular and Pharmacologic Properties of the Anticancer Quinolone Derivative Vosaroxin: A New Therapeutic Agent for Acute Myeloid Leukemia. Drugs.

[56] Lancet J.E., Ravandi F., Ricklis R.M., Cripe L.D., Kantarjian H.M., Giles F.J., List A.F., Chen T., Allen R.S., Fox J.A. (2011). A Phase Ib Study of Vosaroxin, an Anticancer Quinolone Derivative, in Patients with Relapsed or Refractory Acute Leukemia. Leukemia.

[57] Mohammed H.H.H., Abd El-Hafeez A.A., Abbas S.H., Abdelhafez E.-S.M.N., Abuo-Rahma G.E.-D.A. (2016). New Antiproliferative 7-(4-(N-Substituted Carbamoylmethyl)Piperazin-1-Yl) Derivatives of Ciprofloxacin Induce Cell Cycle Arrest at G2/M Phase. Bioorg. Med. Chem..

[58] Chrzanowska A., Roszkowski P., Bielenica A., Olejarz W., Stępień K., Struga M. (2020). Anticancer and Antimicrobial Effects of Novel Ciprofloxacin Fatty Acids Conjugates. Eur. J. Med. Chem..

[59] Chen R., Zhang H., Ma T., Xue H., Miao Z., Chen L., Shi X. (2019). Moxifloxacin/Gatifloxacin-1,2,3-triazole-isatin Hybrids with Hydrogen-Bond Donor and Their In Vitro Anticancer Activity. J. Heterocycl. Chem..

[60] Jiang D., Zhang G. (2019). Ciprofloxacin/Gatifloxacin-1,2,3-triazole-isatin Hybrids and Their in Vitro Anticancer Activity. J. Heterocycl. Chem..

[61] Wang X., Jiang X., Sun S., Liu Y. (2018). Synthesis and Biological Evaluation of Novel Quinolone Derivatives Dual Targeting Histone Deacetylase and Tubulin Polymerization as Antiproliferative Agents. RSC Adv..

[62] Kumar A.R., Boddupally V.L., Rao P.S., Narsaiah B., Sriram D., Sowjanya P. (2015). Synthesis and Biological Evaluation of Novel N1-Decyl and C7- Sec Amine Substituted Fluoroquinolones as Antitubercular and Anticancer Agents. Indian J. Chem. Sect. B.

[63] Rajulu G.G., Bhojya Naik H.S., Viswanadhan A., Thiruvengadam J., Rajesh K., Ganesh S., Jagadheshan H., Kesavan P.K. (2014). New Hydroxamic Acid Derivatives of Fluoroquinolones: Synthesis and Evaluation of Antibacterial and Anticancer Properties. Chem. Pharm. Bull..

[64] Saeed B.B., Al-Iraqi M.A., Abachi F.T. (2012). Synthesis of New 1,2 Dithiol 3-Thione Fluoroquinolone Esters Possessing Anticancer Activity in-Vitro. IJVS.

[65] Azéma J., Guidetti B., Korolyov A., Kiss R., Roques C., Constant P., Daffé M., Malet-Martino M. (2011). Synthesis of Lipophilic Dimeric C-7/C-7-Linked Ciprofloxacin and C-6/C-6-Linked Levofloxacin Derivatives. Versatile in Vitro Biological Evaluations of Monomeric and Dimeric Fluoroquinolone Derivatives as Potential Antitumor, Antibacterial or Antimycobacterial Agents. Eur. J. Med. Chem..

[66] Patitungkho S., Adsule S., Dandawate P., Padhye S., Ahmad A., Sarkar F.H. (2011). Synthesis, Characterization and Anti-Tumor Activity of Moxifloxacin–Copper Complexes against Breast Cancer Cell Lines. Bioorg. Med. Chem. Lett..

[67] Foroumadi A., Emami S., Rajabalian S., Badinloo M., Mohammadhosseini N., Shafiee A. (2009). N-Substituted Piperazinyl Quinolones as Potential Cytotoxic Agents: Structure–Activity Relationships Study. Biomed. Pharmacother..

[68] Pardakhty A., Foroumadi A., Hashemi M., Rajabalian S., Heidari M.R. (2007). In Vitro Cytotoxicity and Phototoxicity of N-Piperazinyl Quinolone Derivatives with a 2-Thienyl Group. Toxicol. Vitro.

[69] Rajabalian S., Foroumadi A., Emami S. (2007). Functionalized N-(2-Oxyiminoethyl) Piperazinyl Quinolones as New Cytotoxic Agents. J. Pharm. Pharm. Sci..

[70] Samir M., Ramadan M., Hamed M., Osman M., Abou=Rahma G. (2021). Recent Strategies in Design of Antitumor and Antibacterial Fluoroquinolones. J. Adv. Biomed. Pharm. Sci..

[71] Thadepalli H., Salem F., Chuah S.K., Gollapudi S. (2005). Antitumor Activity of Trovafloxacin in an Animal Model. Vivo.

[72] Moldovan O.-L., Rusu A., Tanase C., Vari C.-E. (2021). Glutamate—A Multifaceted Molecule: Endogenous Neurotransmitter, Controversial Food Additive, Design Compound for Anti-Cancer Drugs. A Critical Appraisal. Food Chem. Toxicol..

[73] Moldovan O.-L., Sandulea A., Lungu I.-A., Gâz A., Rusu A. (2023). Identification of Some Glutamic Acid Derivatives with Biological Potential by Computational Methods. Molecules.

[74] Daina A., Michielin O., Zoete V. (2017). SwissADME: A Free Web Tool to Evaluate Pharmacokinetics, Drug-Likeness and Medicinal Chemistry Friendliness of Small Molecules. Sci. Rep..

[75] Arnott J.A., Planey S.L. (2012). The Influence of Lipophilicity in Drug Discovery and Design. Expert. Opin. Drug Discov..

[76] Mannhold R., Poda G.I., Ostermann C., Tetko I.V. (2009). Calculation of Molecular Lipophilicity: State-of-the-Art and Comparison of LogP Methods on More than 96,000 Compounds. J. Pharm. Sci..

[77] Cheng T., Zhao Y., Li X., Lin F., Xu Y., Zhang X., Li Y., Wang R., Lai L. (2007). Computation of Octanol−Water Partition Coefficients by Guiding an Additive Model with Knowledge. J. Chem. Inf. Model..

[78] Wildman S.A., Crippen G.M. (1999). Prediction of Physicochemical Parameters by Atomic Contributions. J. Chem. Inf. Comput. Sci..

[79] Moriguchi I., Hirono S., Liu Q., Nakagome I., Matsushita Y. (1992). Simple Method of Calculating Octanol/Water Partition Coefficient. Chem. Pharm. Bull..

[80] Moriguchi I., Hirono S., Nakagome I., Hirano H. (1994). Comparison of Reliability of Log P Values for Drugs Calculated by Several Methods. Chem. Pharm. Bull..

[81] Lipinski C.A., Lombardo F., Dominy B.W., Feeney P.J. (1997). Experimental and Computational Approaches to Estimate Solubility and Permeability in Drug Discovery and Development Settings. Adv. Drug Deliv. Rev..

[82] Ro P., Rh G. (1992). Predicting Skin Permeability. Pharm. Res..

[83] Teague S.J., Davis A.M., Leeson P.D., Oprea T. (1999). The Design of Leadlike Combinatorial Libraries. Angew. Chem. Int. Ed..

[84] Liu W., Liang Y., Si X. (2020). Hydroxamic Acid Hybrids as the Potential Anticancer Agents: An Overview. Eur. J. Med. Chem..

[85] Fathi-Karkan S., Arshad R., Rahdar A., Ramezani A., Behzadmehr R., Ghotekar S., Pandey S. (2023). Recent Advancements in the Targeted Delivery of Etoposide Nanomedicine for Cancer Therapy: A Comprehensive Review. Eur. J. Med. Chem..

[86] Aisner J., Lee E.J. (1991). Etoposide. Current and Future Status. Cancer.

[87] Zhang W., Gou P., Dupret J.-M., Chomienne C., Rodrigues-Lima F. (2021). Etoposide, an Anticancer Drug Involved in Therapy-Related Secondary Leukemia: Enzymes at Play. Transl. Oncol..

[88] Wu C.-C., Li T.-K., Farh L., Lin L.-Y., Lin T.-S., Yu Y.-J., Yen T.-J., Chiang C.-W., Chan N.-L. (2011). Structural Basis of Type II Topoisomerase Inhibition by the Anticancer Drug Etoposide. Science.

[89] Holliday J.D., Salim N., Whittle M., Willett P. (2003). Analysis and Display of the Size Dependence of Chemical Similarity Coefficients. J. Chem. Inf. Comput. Sci..

[90] Jasial S., Hu Y., Vogt M., Bajorath J. (2016). Activity-Relevant Similarity Values for Fingerprints and Implications for Similarity Searching. F1000Research.

[91] Zahoránszky-Kőhalmi G., Bologa C.G., Oprea T.I. (2016). Impact of Similarity Threshold on the Topology of Molecular Similarity Networks and Clustering Outcomes. J. Cheminform..

[92] Mellor C.L., Marchese Robinson R.L., Benigni R., Ebbrell D., Enoch S.J., Firman J.W., Madden J.C., Pawar G., Yang C., Cronin M.T.D. (2019). Molecular Fingerprint-Derived Similarity Measures for Toxicological Read-across: Recommendations for Optimal Use. Regul. Toxicol. Pharmacol..

[93] Miranda-Quintana R.A., Bajusz D., Rácz A., Héberger K. (2021). Differential Consistency Analysis: Which Similarity Measures Can Be Applied in Drug Discovery?. Mol. Inform..

[94] Bero S.A., Muda A.K., Choo Y.H., Muda N.A., Pratama S.F. (2017). Similarity Measure for Molecular Structure: A Brief Review. J. Phys. Conf. Ser..

[95] Chung N.C., Miasojedow B., Startek M., Gambin A. (2019). Jaccard/Tanimoto Similarity Test and Estimation Methods for Biological Presence-Absence Data. BMC Bioinform..

[96] Nikumbh A.B., Rathi M.V. (2017). Study of Molar Refraction and Polarizability Constant of Aqueous Solutions of KNO_3_ and KBrO_3_ at Different Temperatures. Int. J. Adv. Res..

[97] Prasanna S., Doerksen R.J. (2009). Topological Polar Surface Area: A Useful Descriptor in 2D-QSAR. Curr. Med. Chem..

[98] Zhao Y.H., Abraham M.H., Zissimos A.M. (2003). Determination of McGowan Volumes for Ions and Correlation with van Der Waals Volumes. J. Chem. Inf. Comput. Sci..

[99] Tsantili-Kakoulidou A., Demopoulos J.V. (2021). Drug-like Properties and Fraction Lipophilicity Index as a Combined Metric. ADMET DMPK.

[100] Saiful Islam M., Mitra S. (2023). Effect of Nano Graphene Oxide (NGO) Incorporation on the Lipophilicity of Hydrophobic Drugs. Hybrid Adv..

[101] Marvinsketch (Version 23.11). https://chemaxon.com/marvin.

[102] Wager T.T., Hou X., Verhoest P.R., Villalobos A. (2010). Moving beyond Rules: The Development of a Central Nervous System Multiparameter Optimization (CNS MPO) Approach To Enable Alignment of Druglike Properties. ACS Chem. Neurosci..

[103] Chauhan H.H., Chavan M.D., Choudhary R.R., Madkaikar H.M., Dalvi T.S., Shah N.J. (2022). Screening of Phytochemicals from Couroupita Guianensis as Drug Candidates against Lethal Diseases Using Insilico Analysis. Int. J. Appl. Chem. Biol. Sci..

[104] Rameshbabu S., Alehaideb Z., Alghamdi S.S., Suliman R.S., Almourfi F., Yacoob S.A.M., Venkataraman A., Messaoudi S., Matou-Nasri S. (2024). Identification of Anastatica hierochuntica L. Methanolic Leaves Extract-Derived Metabolites Exhibiting Xanthine Oxidase Inhibitory Activities: In Vitro and in Silico Approaches. Metabolites.

[105] Elbouzidi A., Taibi M., Laaraj S., Loukili E.H., Haddou M., Hachlafi N.E., Mrabti H.N., Baraich A., Bellaouchi R., Asehraou A. (2024). Chemical Profiling of Volatile Compounds of the Essential Oil of Grey-Leaved Rockrose (*Cistus albidus* L.) and Its Antioxidant, Anti-Inflammatory, Antibacterial, Antifungal, and Anticancer Activity in Vitro and in Silico. Front. Chem..

[106] SMARTCyp (Version 3.0). https://smartcyp.sund.ku.dk/mol_to_som.

[107] Olsen L., Montefiori M., Tran K.P., Jørgensen F.S. (2019). SMARTCyp 3.0: Enhanced Cytochrome P450 Site-of-Metabolism Prediction Server. Bioinformatics.

[108] Athar M., Sona A.N., Bekono B.D., Ntie-Kang F. (2019). Fundamental Physical and Chemical Concepts behind “Drug-Likeness” and “Natural Product-Likeness”. Phys. Sci. Rev..

[109] Doak B.C., Over B., Giordanetto F., Kihlberg J. (2014). Oral Druggable Space beyond the Rule of 5: Insights from Drugs and Clinical Candidates. Chem. Biol..

[110] Santos G.B., Ganesan A., Emery F.S. (2016). Oral Administration of Peptide-Based Drugs: Beyond Lipinski’s Rule. ChemMedChem.

[111] DeGoey D.A., Chen H.-J., Cox P.B., Wendt M.D. (2018). Beyond the Rule of 5: Lessons Learned from AbbVie’s Drugs and Compound Collection: Miniperspective. J. Med. Chem..

[112] Yadav G., Ganguly S., Murugesan S., Dev A. (2015). Synthesis, Anti-HIV, Antimicrobial Evaluation and Structure Activity Relationship Studies of Some Novel Benzimidazole Derivatives. AIA.

[113] Molecular Properties Prediction—Osiris Property Explorer. https://www.organic-chemistry.org/prog/peo/.

[114] Martin Y.C. (2005). A Bioavailability Score. J. Med. Chem..

[115] Price G., Patel D.A. (2024). Drug Bioavailability. StatPearls.

[116] Rajan R., Karthikeyan S., Desikan R. (2024). Synthesis, Structural Elucidation, In Silico and In Vitro Studies of New Class of Methylenedioxyphenyl-Based Amide Derivatives as Potential Myeloperoxidase Inhibitors for Cardiovascular Protection. ACS Omega.

[117] Polinsky A. (2008). Lead-Likeness and Drug-Likeness. The Practice of Medicinal Chemistry.

[118] Hann M.M., Oprea T.I. (2004). Pursuing the Leadlikeness Concept in Pharmaceutical Research. Curr. Opin. Chem. Biol..

[119] SwissADME. http://www.swissadme.ch/.

[120] Skoraczyński G., Kitlas M., Miasojedow B., Gambin A. (2023). Critical Assessment of Synthetic Accessibility Scores in Computer-Assisted Synthesis Planning. J. Cheminform..

[121] Ertl P., Schuffenhauer A. (2009). Estimation of Synthetic Accessibility Score of Drug-like Molecules Based on Molecular Complexity and Fragment Contributions. J. Cheminform..

[122] Molinspiration Calculation of Molecular Properties and Bioactivity Score (Version v2022.08). https://molinspiration.com/cgi/properties.

[123] Way2Drug—Main (Version 2.0). http://www.way2drug.com/PASSOnline/index.php.

[124] Cui C., Zhang Y., Wang L., Liu H., Cui G. (2010). Enhanced Anticancer Activity of Glutamate Prodrugs of All-Trans Retinoic Acid. J. Pharm. Pharmacol..

[125] Tokura Y. (1998). Quinolone Photoallergy: Photosensitivity Dermatitis Induced by Systemic Administration of Photohaptenic Drugs. J. Dermatol. Sci..

[126] Tokura Y. (2000). Immune Responses to Photohaptens: Implications for the Mechanisms of Photosensitivity to Exogenous Agents. J. Dermatol. Sci..

[127] Rusu A., Munteanu A.-C., Arbănași E.-M., Uivarosi V. (2023). Overview of Side-Effects of Antibacterial Fluoroquinolones: New Drugs versus Old Drugs, a Step Forward in the Safety Profile?. Pharmaceutics.

[128] OECD Test Guidelines for Chemicals—OECD. https://www.oecd.org/chemicalsafety/testing/oecdguidelinesforthetestingofchemicals.html.

[129] Croner-i Harmonisation of Classification of Corrosives for Supply and Transport. https://app.croneri.co.uk/feature-articles/harmonisation-classification-corrosives-supply-and-transport?product=139.

[130] Barratt M.D., Dixit M.B., Jones P.A. (1996). The Use of in Vitro Cytotoxicity Measurements in QSAR Methods for the Prediction of the Skin Corrosivity Potential of Acids. Toxicol. Vitro.

[131] Scott L., Eskes C., Hoffmann S., Adriaens E., Alepée N., Bufo M., Clothier R., Facchini D., Faller C., Guest R. (2010). A Proposed Eye Irritation Testing Strategy to Reduce and Replace in Vivo Studies Using Bottom-Up and Top-Down Approaches. Toxicol. Vitro.

[132] Prinsen M.K., Hendriksen C.F.M., Krul C.A.M., Woutersen R.A. (2017). The Isolated Chicken Eye Test to Replace the Draize Test in Rabbits. Regul. Toxicol. Pharmacol..

[133] Corbeil C.R., Englebienne P., Moitessier N. (2007). Docking Ligands into Flexible and Solvated Macromolecules. 1. Development and Validation of FITTED 1.0. J. Chem. Inf. Model..

[134] Corbeil C.R., Englebienne P., Yannopoulos C.G., Chan L., Das S.K., Bilimoria D., L’heureux L., Moitessier N. (2008). Docking Ligands into Flexible and Solvated Macromolecules. 2. Development and Application of Fitted 1.5 to the Virtual Screening of Potential HCV Polymerase Inhibitors. J. Chem. Inf. Model..

[135] Corbeil C.R., Moitessier N. (2009). Docking Ligands into Flexible and Solvated Macromolecules. 3. Impact of Input Ligand Conformation, Protein Flexibility, and Water Molecules on the Accuracy of Docking Programs. J. Chem. Inf. Model..

[136] Molecular Forecaster Resources, (Version 6453). https://molecularforecaster.com/resources/.

[137] Wan S., Bhati A.P., Zasada S.J., Coveney P.V. (2020). Rapid, Accurate, Precise and Reproducible Ligand–Protein Binding Free Energy Prediction. Interface Focus.

[138] 21.4: Chemistry of Acid Halides. https://chem.libretexts.org/Bookshelves/Organic_Chemistry/Organic_Chemistry_(Morsch_et_al.)/21%3A_Carboxylic_Acid_Derivatives-_Nucleophilic_Acyl_Substitution_Reactions/21.04%3A_Chemistry_of_Acid_Halides.

[139] Sharma P.C., Jain S. (2008). Synthesis and in-Vitro Antibacterial Activity of Some Novel N-nicotinoyl-1-ethyl-6-fluoro-1,4-dihydro-7-piperazin-1-yl-4- oxoquinoline-3-carboxylates. Acta Pol. Pharm. Drug Res..

[140] Systèmes D. BIOVIA Draw for Academics (Version 21.1). https://discover.3ds.com/biovia-draw-academic.

[141] Delaney J.S. (2004). ESOL: Estimating Aqueous Solubility Directly from Molecular Structure. J. Chem. Inf. Comput. Sci..

[142] Ali J., Camilleri P., Brown M.B., Hutt A.J., Kirton S.B. (2012). Revisiting the General Solubility Equation: In Silico Prediction of Aqueous Solubility Incorporating the Effect of Topographical Polar Surface Area. J. Chem. Inf. Model..

[143] Egan W.J., Merz K.M., Baldwin J.J. (2000). Prediction of Drug Absorption Using Multivariate Statistics. J. Med. Chem..

[144] Verber D.F., Johnson S.R., Cheng H.-Y., Smith B.R., Ward K.W., Kopple K.D. (2002). Molecular Properties That Influence the Oral Bioavailability of Drug Candidates. J. Med. Chem..

[145] Ghose A.K., Viswanadhan V.N., Wendoloski J.J. (1999). A Knowledge-Based Approach in Designing Combinatorial or Medicinal Chemistry Libraries for Drug Discovery. 1. A Qualitative and Quantitative Characterization of Known Drug Databases. J. Comb. Chem..

[146] Muegge I., Heald S.L., Brittelli D. (2001). Simple Selection Criteria for Drug-like Chemical Matter. J. Med. Chem..

[147] AquaSol: Predict Aqueous Solublity of Small Molecules Using UG-RNN Ensembles (Version 2.0). http://cdb.ics.uci.edu/cgibin/tools/AquaSolWeb.py.

[148] Lagunin A.A., Dubovskaja V.I., Rudik A.V., Pogodin P.V., Druzhilovskiy D.S., Gloriozova T.A., Filimonov D.A., Sastry N.G., Poroikov V.V. (2018). CLC-Pred: A Freely Available Web-Service for in Silico Prediction of Human Cell Line Cytotoxicity for Drug-like Compounds. PLoS ONE.

[149] Gusar—Create QSAR/QSPR Models on the Basis of the Appropriate Training Sets. http://www.way2drug.com/gusar/acutoxpredict.html.

[150] Jeliazkova N., Martinov M., Tcheremenskaia O.J.K., Networks M., Rydberg P., Avramova S., Kochev N., Jeliazkov V., Iliev L. Toxtree—Toxtree-Toxic Hazard Estimation by Decision Tree Approach. Version 3.1.0.1851. https://toxtree.sourceforge.net/.

[151] Cramer G.M., Ford R.A., Hall R.L. (1976). Estimation of Toxic Hazard—A Decision Tree Approach. Food Cosmet. Toxicol..

[152] Kroes R., Renwick A.G., Cheeseman M., Kleiner J., Mangelsdorf I., Piersma A., Schilter B., Schlatter J., Van Schothorst F., Vos J.G. (2004). Structure-Based Thresholds of Toxicological Concern (TTC): Guidance for Application to Substances Present at Low Levels in the Diet. Food Chem. Toxicol..

[153] Verhaar H.J.M., van Leeuwen C.J., Hermens J.L.M. (1992). Classifying Environmental Pollutants. Chemosphere.

[154] Verhaar H.J.M., Solbé J., Speksnijder J., van Leeuwen C.J., Hermens J.L.M. (2000). Classifying Environmental Pollutants: Part 3. External Validation of the Classification System. Chemosphere.

[155] Benigni R., Bossa C. (2011). Mechanisms of Chemical Carcinogenicity and Mutagenicity: A Review with Implications for Predictive Toxicology. Chem. Rev..

[156] Benigni R., Bossa C., Netzeva T., Rodomonte A., Tsakovska I. (2007). Mechanistic QSAR of Aromatic Amines: New Models for Discriminating between Homocyclic Mutagens and Nonmutagens, and Validation of Models for Carcinogens. Environ. Mol. Mutagen..

[157] Benigni R., Bossa C., Tcheremenskaia O. (2013). Nongenotoxic Carcinogenicity of Chemicals: Mechanisms of Action and Early Recognition through a New Set of Structural Alerts. Chem. Rev..

[158] Gerner I., Schlegel K., Walker J., Hulzebos E. (2004). Use of Physicochemical Property Limits to Develop Rules for Identifying Chemical Substances with No Skin Irritation or Corrosion Potential. QSAR Comb. Sci..

[159] Hulzebos E., Walker J.D., Gerner I., Schlegel K. (2005). Use of Structural Alerts to Develop Rules for Identifying Chemical Substances with Skin Irritation or Skin Corrosion Potential. QSAR Comb. Sci..

[160] Walker J.D., Gerner I., Hulzebos E., Schlegel K. (2005). The Skin Irritation Corrosion Rules Estimation Tool (SICRET). QSAR Comb. Sci..

[161] Gerner I., Liebsch M., Spielmann H. (2005). Assessment of the Eye Irritating Properties of Chemicals by Applying Alternatives to the Draize Rabbit Eye Test: The Use of QSARs and In Vitro Tests for the Classification of Eye Irritation. Altern. Lab. Anim..

[162] Enoch S.J., Ellison C.M., Schultz T.W., Cronin M.T.D. (2011). A Review of the Electrophilic Reaction Chemistry Involved in Covalent Protein Binding Relevant to Toxicity. Crit. Rev. Toxicol..

[163] Enoch S.J., Cronin M.T.D. (2010). A Review of the Electrophilic Reaction Chemistry Involved in Covalent DNA Binding. Crit. Rev. Toxicol..

[164] Rydberg P., Gloriam D.E., Zaretzki J., Breneman C., Olsen L. (2010). SMARTCyp: A 2D Method for Prediction of Cytochrome P450-Mediated Drug Metabolism. ACS Med. Chem. Lett..

[165] Moitessier N., Pottel J., Therrien E., Englebienne P., Liu Z., Tomberg A., Corbeil C.R. (2016). Medicinal Chemistry Projects Requiring Imaginative Structure-Based Drug Design Methods. Acc. Chem. Res..

[166] Trott O., Olson A.J. (2010). AutoDock Vina: Improving the Speed and Accuracy of Docking with a New Scoring Function, Efficient Optimization, and Multithreading. J. Comput. Chem..

[167] Morris G.M., Huey R., Lindstrom W., Sanner M.F., Belew R.K., Goodsell D.S., Olson A.J. (2009). AutoDock4 and AutoDockTools4: Automated Docking with Selective Receptor Flexibility. J. Comput. Chem..

[168] Stoica C.I., Marc G., Pîrnău A., Vlase L., Araniciu C., Oniga S., Palage M., Oniga O. (2016). Thiazolyl-oxadiazole Derivatives Targeting Lanosterol 14α-demethylase As Potential Antifungal Agents: Design, Synthesis and Molecular Docking Studies. Farmacia.

[169] Systèmes D. Free Download: BIOVIA Discovery Studio Visualizer (Version 21.1.0.20298). https://discover.3ds.com/discovery-studio-visualizer-download.

[170] Pettersen E.F., Goddard T.D., Huang C.C., Couch G.S., Greenblatt D.M., Meng E.C., Ferrin T.E. (2004). UCSF Chimera—A Visualization System for Exploratory Research and Analysis. J. Comput. Chem..

