# Supplementary material for: In Silico Evaluation of Some Computer-Designed Fluoroquinolone–Glutamic Acid Hybrids as Potential Topoisomerase II Inhibitors with Anti-Cancer Effect
Source: Pharmaceuticals (Basel). 2024 Nov 26;17(12):1593. doi: 10.3390/ph17121593 (PMC11679884; doi:10.3390/ph17121593)
Supplement: Supplementary file 1 [file pharmaceuticals-17-01593-s001.zip › pharmaceuticals-3300868-supplementary.pdf]

SUPPLEMENTARY MATERIAL

Table S1. FQs as anti-cancer agents

No.	Fluoroquinolone	Cancer cell type	Mechanism of action	Ref.
1.	Ciprofloxacin	Human bladder cancer cell lines (HTB9)	-Induction of cell cycle arrest at the S/G2-M checkpoint<br>-Decrease in cyclin B and E levels and cdk2 dephosphorylation<br>-Increase of Bax levels, with changes in Bax/Bcl-2 balance, conducting to apoptosis	[1]
		Human colorectal cancer cell lines (HT-29, SW-403)	Induction of mitochondrial DNA selective damage, with depletion of intracellular ATP reserves. The consequence is the cell cycle arrest at the S/G2-M checkpoint, with decreased levels of cyclin B and E, dephosphorylation of cdk2, and increased levels of the pro-apoptotic Bax (through the activation of pro-caspase 9)	[2]
		Ovarian cancer cell lines (CHO AA8)(hamsters)	Increase in the number of apoptotic cells, depending (proportional) on ciprofloxacin time of action and concentration.	[3]
		Human hepatocellular carcinoma cell lines (HepG2, Huh7)	Increased production of IL-1 β, TNF-α, and polarisation of $CD86^{+}CD206^{-}$ macrophages, with the inhibition of $CD86^{-}CD206^{+}$ macrophage polarisation. The consequences are the promotion of apoptosis, inhibiting tumour cell proliferation, and reducing metastasis and phosphoinositide 3-kinase/AKT signalling pathway in the liver cancer cell line.	[4]
		Melanoma cell lines (COLO829)	Stagnation of the S phase of the cell cycle.	[5]
		Human non-small cell lung cancer cell lines (A549)	Stagnation of cells at G2/M checkpoint.	[6]
		Human triple-negative breast cancer cell lines (MDA-MB-231)	-Cell cycle arrest in the S phase<br>-Increased p53 expression, with apoptosis induction through the p53/Bax/Bcl-2 signaling pathway	[7]
		Human glioblastoma A-172 cell line	Increased levels of Bax (apoptosis marker) and decreased concentration of Bcl-2 (anti-apoptotic marker). Increased levels of malondialdehyde in cancer cells (oxidative stress marker).	[8]
		Human pancreatic cancer cell lines (MIA PaCa-2 and Panc-1 cells)	The mechanism involves cell cycle arrest in the S phase, with decreased levels of p27, p21, CDK2, cyclin A, and cyclin E. Activation of caspases 8, 9, and 3 also occurs, triggering the mitochondrial apoptotic pathway. There is also a decrease in the anti-apoptotic protein Bcl-xL and an increase in the pro-apoptotic protein Bak.	[9]
2.	Gatifloxacin	Pancreatic cancer cell lines (MIA PaCa-2 and Panc-1)	Stopping the cell cycle in the G2 phase by inactivating the cdc2-cyclin B1 complex; this occurs by phosphorylating cdc2 at Tyr15, through p53 activation.	[10]
3.	Moxifloxacin	Human	The mechanism involves cell cycle arrest in the S phase, with	[9]

No.	Fluoroquinolone	Cancer cell type	Mechanism of action	Ref.
		pancreatic cancer cell lines (MIA PaCa-2 and Panc-1 cells)	decreased levels of p27, p21, CDK2, cyclin A, and cyclin E. Activation of caspases 8, 9, and 3 also occurs, triggering the mitochondrial apoptotic pathway. There is also a decrease in the anti-apoptotic protein Bcl-cL and an increase in the pro-apoptotic protein Bak.	
4.	Levofloxacin<br>Enoxacin<br>Ofloxacin<br>Fleroxacin	Several human bladder cancer cell lines	-	[11]
5.	Levofloxacin	Human bladder cancer cell lines (T24)	-Cell cycle inhibition in the S phase.<br>-Increase the Bax level and decrease the Bcl-2 level.	[12]
6.	Gemifloxacin	Human colon cancer cell lines (SW480 and SW620)	Anti-proliferative activity.	[13]

Table S2. FQs hybrids with different compounds and their biological effects

No.	FQ derivative	Cancer cell type	Mechanism of action	Ref.
1.	7-(4-(carbamoylmethyl N-substituted)) piperazine 1-yl derivatives of ciprofloxacin	Human non-small cell lung cancer cell lines (A549)	Decreased expression of cyclin B1 and cdc2 proteins, with importance for cell cycle modulation (G2/M phase)	[14]
2.	Ciprofloxacin conjugated with fatty acids	Metastatic prostate cancer cell lines (PC3)	Reduction of cancer cells' IL-6 secretion, with suppression of precancerous inflammatory state, tumour development and progression.	[15]
3.	Moxifloxacin/gatifloxacin -1,2,3-triazole-isatin hybrids	Human lung, liver and breast carcinoma cell lines (A549, HepG2, MCF-7)	-	[16]
4.	Ciprofloxacin/gatifloxaci n-1,2,3-triazole-isatin hybrids	Human lung carcinoma (A549), liver and central nervous system cancer cell lines (HepG2 and SF-268)	-	[17]
5.	Vosaroxin	Phase III clinical trial – adult patients with relapsed or refractory advanced leukaemia, with no expectation of response to standard therapy	Selective damage of DNA by intercalation in its structure and inhibition of type II Topoisomerase, leading to apoptosis	[18,19]
6.	Hybrids of FQs with the boron atom	Cervical cancer cell lines (SiHa and Caski)	Inhibition of cancer cells proliferation.	[20]
7.	Conjugates of levofloxacin with histone deacetylase inhibitors	Human lung adenocarcinoma cells (A549); Liver cancer cells (Hepg2);	Inhibition of histone deacetylases and tubulin polymerisation.	[21]

No.	FQ derivative	Cancer cell type	Mechanism of action	Ref.
		Breast cancer cell lines (MCF-7);<br>Prostate cancer cell lines (PC-3);<br>Human cervical cancer cell lines (HeLa).		
8.	Analogues of N1-decyl and C7-sec amine FQ	Human cervical cancer cell lines (HeLa);<br>Breast carcinoma cell lines (MDA-MB-231);<br>Human pancreatic carcinoma cell lines (MIA PaCa);<br>Human neuroblastoma cell lines (IMR32).	Anti-proliferative effect.	[22]
9.	FQ derivatives with a hydroxamic acid residue replacing the carboxyl group from position no. 3 on the quinolone nucleus	Human lung adenocarcinoma cell lines (A549);<br>Human cervical adenocarcinoma cell lines (HeLa);<br>Human colon adenocarcinoma cell lines (HCT-116);<br>Human pancreatic carcinoma cell lines (PANC1).	Cell growth inhibition.	[23]
10.	N-[4-piperazinyl]-ciprofloxacin-chalcone hybrid type	Leukemic cell lines.	Inhibition of cancer cells proliferation.	[24]
11.	Esters formed by the condensation of (p-hydroxyphenyl)-1,2-dithiol-3-thione with FQ	Renal cancer cell lines (type 786.0 and UO-31);<br>CCRF-CEM leukemic cell lines;<br>Melanoma cancer cell lines (LOX. IMVI)	It is considered that the presence of the -SH group in the molecule leads to H_2S generation, which, along with the DNA inhibition effect, produces increased tumour regression.	[25]
12.	C7-C7 ciprofloxacin dimers (7-(4-(alkanoyl and oxoethyl alkanoate) piperazine bond type) or C6-C6 levofloxacin dimers (C-6-(N alkylcarboxamide) bond type)	Human glioblastoma cell lines (U373-MG);<br>Human colon cancer cell lines (LoVo);<br>Human breast carcinoma cell lines (MCF-7)	Inhibarea dezvoltării liniei celulare canceroase prin efectul antiproliferativ.<br>Inhibition of cancer cell line development through anti-proliferative effect.	[26]
13.	Moxifloxacin-copper complexes	Human cancer cell lines:<br>- T47D (hormone-dependent)<br>- MDA-MB-231 and BT-20 (hormone-independent)	Anti-proliferative activity against breast cancer cell lines, without any toxicity against non-tumorigenic breast epithelial cell lines.	[27]
14.	N-piperazinylquinolone derivatives	Human melanoma cell lines (SKMEL-3);<br>Human breast cancer cell	Cytotoxic activity.	[28]

No.	FQ derivative	Cancer cell type	Mechanism of action	Ref.
		lines (MCF-7);<br>Human epidermal carcinoma cell lines (A431);<br>Human bladder carcinoma cell lines (EJ);<br>Human colon carcinoma cell lines (SW480);<br>Human cervical carcinoma cell lines (KB).		
15.	N-[2 substituted-2-(2-thienyl)ethyl]piperazinyl quinolone derivatives	Human oral epithelial carcinoma cell lines (KB);<br>Human squamous carcinoma cell lines (A431)<br>(Both cell types are modified human keratinocytes)	It is considered that the local application of FQs, such as ciprofloxacin, ofloxacin or levofloxacin, could induce the expression of some metalloproteinases in the non-debrided corneal epithelium, leading to the deposition and degradation of the extracellular matrix.	[29]
16.	N-(2-oxyimino ethyl)piperazinyl quinolone compounds	Renal cancer cell lines (ACHN);<br>Breast cancer cell lines (MCF-7);<br>Glioma cell lines (A172);<br>Melanoma cell lines (SKMEL-3);<br>Lung cancer cell lines (A549);<br>Ovarian cancer cell lines (A2780-CP)<br>Oral cancer cell lines (KB).	Possible interaction with DNA-topoisomerase II complex, with DNA alteration.	[30]

Table S3. The structure-activity relationship of FQ is detailed for each position of the quinolone nucleus

Position on the quinolone ring	Structure-activity relationship in FQs
Position 1	The nitrogen atom characterises position 1, which is essential for anti-cancer activity. The ethyl radical is a substituent found in this position, but replacing it with the cyclopropyl radical leads to enhanced potency and an improved anti-proliferative effect due to increased interaction with type II topoisomerase. Also, the thiazolyl, phenyl or methoxy radicals bring improvements to the molecule if they are found in this position [31-33].
Position 2	The activity against type II topoisomerase can be increased by introducing a phenyl radical or aromatic heterocycles in the C-2 position through a methylene group. If an aromatic phenyl-type radical with several hydroxyl groups is added in this position, for example, a 2,6-dihydroxybenzyl, the cytotoxic activity can be enhanced [33,34]. Also, linking an izothiazole ring to the quinoline nucleus via the 2nd and 3rd positions enhances FQ's anti-cancer activity and potency [31,33].

Position on the quinolone ring	Structure-activity relationship in FQs
Position 3	According to scientific literature, the carboxyl group from the C-3 position is essential for the antibacterial activity of FQs. Both the carboxyl group from the C-3 position and the ketone group at the C-4 position can form hydrogen bonds with the target enzymes, and together, they can chelate metals.<br>Although considered essential, some studies have concluded that by replacing these groups with hydrogen atoms, the compound can exhibit toxicity to eukaryotic topoisomerase II. However, attention must be paid to the functional groups in the C-2 and C-3 positions and the cycle's planarity [50]. Thus, removing the carboxyl group from the C-3 position and introducing a phenyl group or an aromatic heterocycle at the C-2 position increases the ability to inhibit topoisomerase II [34]. Considering that the carboxyl group in the C-3 position is not mandatory for antitumor activity, its replacement with an oxadiazole or thiadiazine type heterocycle was studied, leading to increased antitumor activity [35].<br>Also, replacing the carboxyl group of ciprofloxacin with a hydroxamic acid group increases the ability to inhibit the proliferation of colon and lung adenocarcinoma cell lines [35].
Position 4	The ketone carbonyl group at this position is essential for the FQ to treat bacterial infections effectively, but it is also thought to be beneficial for anti-cancer activity. However, it appears that replacing it with alkoxy or amino functional groups can enhance this effect [31-33].
Position 5	The C-5 position is significant for a FQ drug, as it influences the steric configuration, the planarity of the compound, the cell permeability and its affinity for the target [32,33]. Among the structural changes at this position that can improve the anti-cancer effect of FQs is the introduction of an amino group, aryl radicals or aromatic heterocycles [31,33].
Position 6	Adding a halogen atom, especially fluorine, to the C-6 position facilitates the entry of FQ into the bacterial cell and confers anti-tumour effects on the molecule. Moreover, difluorinated derivatives of FQs with fluorine atoms in the C-6 and C-8 positions are valuable anti-cancer agents by inhibiting eukaryotic topoisomerase. At the same time, the derivative with a methoxy group at C-6 can also have a cytotoxic effect [31,33].
Position 7	This position is considered to have the most significant degree of adaptability to structural modifications. Changes at this level can influence the compound's potency, spectrum, safety, physicochemical, and pharmacokinetic properties, such as bioavailability [23,26,28,32,33]. The C-7 position significantly impacts FQs' activity, being considered to have direct interaction with topoisomerases from the enzyme-DNA complex [31].<br>For example, it is believed that new FQ analogues can be developed by structural changes at the C-7 position of ciprofloxacin, diminishing the zwitterionic effect and influencing the hydrophilic nature, with improved anti-proliferative activity of ciprofloxacin [36]. According to the scientific literature, the increase in the lipophilicity of FQ-type compounds is desired, and the most suitable substituents for this nucleus position would be cyclic radicals or aryl-type radicals [35]. The increase in the number of aromatic and condensed rings contributes to enhancing the cytotoxic effect of FQs [31-33]. An example is the 4-hydroxyphenyl radical, which is important for quinolone potency against mammalian topoisomerase II [33,34]. It seems that introducing bulky groups, such as diphenyldimethylphosphine, in this position increases the anti-cancer activity and minimises the genotoxic effect [35].<br>In addition, certain halogenated FQs containing an aniline residue at C-7 have shown remarkable activity against some cancer cell lines [37]. The cyclic amine residue in the C-7 position would be responsible for decreasing the toxicity and improving the solubility of the

Position on the quinolone ring	Structure-activity relationship in FQs
	compounds [32]. According to some studies, the optimal substituents are the heterocycles with 5 or 6 nitrogen atoms [23]. Also, introducing an unsaturated aminoazabicyclo group in the C-7 position confers antitumor activity to the compound [34].<br>The methyl substituent of the piperazine ring at the C-7 position influences the potency against mammalian enzymes. The stereochemistry also influences the opposite sense since, for example, derivatives of the 3,5-dimethylpiperazinyl type can stimulate enzyme-mediated DNA cleavage only in the trans configuration [26].<br>Such changes are also targeted in the case of FQs' antibacterial action development since an increase in the volume of the C-7 substituent leads to an enhanced antibacterial potency against gram-positive bacteria. Introducing a cyclic aliphatic substituent at the C-7 position is mandatory for antibacterial activity [26]. Substitution of the quinolone at the C-7 position with piperazinyl, piperidinyl, and pyrolidinyl moieties broadens the spectrum of antibacterial activities. In addition, introducing the fluorine atom at the C-6 of the quinolone ring and cyclic diamino piperazine in the C-7 position led to the FQ derivatives with activity against Gram-positive, Gram-negative, and anaerobic bacteria [35].
Position 8	The C-8 position allows several alternatives to improve the antitumor effect. An important aspect that must be considered is that the substituents in the C-8 position, such as those in the C-5 position, affect the steric configuration and planarity of the compound, modulating the affinity and the binding of the molecule to the biological target. At the same time, it is considered that they would be responsible for the phototoxicity produced by FQs [31-33]. Thus, a nitrogen atom in the C-8 position, which can accept protons, confers anti-cancer properties to the structure [37].<br>At the same time, similar to position 6, the introduction of halogen atoms at C-8, especially the fluorine atom, leads to the potentiation of the cytotoxic effect, with the alteration of topoisomerase II of eukaryotic cells [31-34]. As previously mentioned, two halogen atoms in C-6 and C-8 positions seriously contribute to the cytotoxic effect of FQs by stabilising the complex with human topoisomerase II. In difluorinated structures of this type, the change of the ethyl substituent from N-1 to cyclopropyl is critical for the anti-proliferative effect [34].<br>In addition, cyclopropyl and methoxy groups are considered ideal substituents at the C-8 position of FQ, potentiating the cytotoxic activity. Also, introducing a ring linked to the quinoline nucleus through C-7 and C-8 positions would produce the same effect [31,32].

Table S4. IUPAC name, SMILES string, InChI string and InChI key of the hybrids and etoposide

Compound	IUPAC name	SMILES string	InChI string	Inchi key
CF-3-GLA/A1	2-[(1-cyclopropyl-6-fluoro-4-oxo-7-piperazin-1-yl-quinoline-3-carbonyl)amino]pentanedioic acid	OC(=O)CCC(NC(=O)C1=CN(C2CC2)c3cc(N4CCNCC4)c(F)cc3C1=O)C(=O)O	InChI=1S/C22H25FN4O6/c23-15-9-13-17(10-18(15)26-7-5-24-6-8-26)27(12-1-2-12)11-14(20(13)30)21(31)25-16(22(32)33)3-4-19(28)29/h9-12,16,24H,1-8H2,(H,25,31)(H,28,29)(H,32,33)	XQJVBAZAKZYBKZ-UHFFFAOYSA-N
NF-3-GLA/A2	2-[(1-ethyl-6-fluoro-4-oxo-7-piperazin-1-yl-quinoline-3-	CCN1C=C(C(=O)NC(CCC(=O)O)C(	InChI=1S/C21H25FN4O6/c1-2-25-11-13(20(30)24-15(21(31)32)3-4-	ISKDHAWRKASJCO-

Compound	IUPAC name	SMILES string	InChI string	Inchi key
	carbonyl)amino]pentanedioic acid	=O)O)C(=O)c2cc(F)c(cc12)N3CCNCC3	18(27)28)19(29)12-9-14(22)17(10-16(12)25)26-7-5-23-6-8-26/h9-11,15,23H,2-8H2,1H3,(H,24,30)(H,27,28)(H,31,32)	UHFFFAOYSA-N
PF-3-GLA/ A3	2-[[1-ethyl-6-fluoro-7-(4-methylpiperazin-1-yl)-4-oxo-quinoline-3-carbonyl]amino]pentanedioic acid	CCN1C=C(C(=O)NC(CCC(=O)O)C(=O)O)C(=O)c2cc(F)c(cc12)N3CCN(C)CC3	InChI=1S/C22H27FN4O6/c1-3-26-12-14(21(31)24-16(22(32)33)4-5-19(28)29)20(30)13-10-15(23)18(11-17(13)26)27-8-6-25(2)7-9-27/h10-12,16H,3-9H2,1-2H3,(H,24,31)(H,28,29)(H,32,33)	JUOQHXGLMAPTLI-UHFFFAOYSA-N
OF-3-GLA/ A4	2-[[7-fluoro-2-methyl-6-(4-methylpiperazin-1-yl)-10-oxo-4-oxa-1-azatricyclo[7.3.1.05,13]trideca-5(13),6,8,11-tetraene-11-carbonyl]amino]pentanedioic acid	CC1COc2c(N3CCN(C)CC3)c(F)cc4C(=O)C(=CN1c24)C(=O)NC(CCC(=O)O)C(=O)O	InChI=1S/C23H27FN4O7/c1-12-11-35-21-18-13(9-15(24)19(21)27-7-5-26(2)6-8-27)20(31)14(10-28(12)18)22(32)25-16(23(33)34)3-4-17(29)30/h9-10,12,16H,3-8,11H2,1-2H3,(H,25,32)(H,29,30)(H,33,34)	JMSBAKPPKQAKDR-UHFFFAOYSA-N
LF-3-GLA/ A5	2-[[(2S)-7-fluoro-2-methyl-6-(4-methylpiperazin-1-yl)-10-oxo-4-oxa-1-azatricyclo[7.3.1.05,13]trideca-5(13),6,8,11-tetraene-11-carbonyl]amino]pentanedioic acid	C[C@H]1COc2c(N3CCN(C)CC3)c(F)cc4C(=O)C(=CN1c24)C(=O)NC(CCC(=O)O)C(=O)O	InChI=1S/C23H27FN4O7/c1-12-11-35-21-18-13(9-15(24)19(21)27-7-5-26(2)6-8-27)20(31)14(10-28(12)18)22(32)25-16(23(33)34)3-4-17(29)30/h9-10,12,16H,3-8,11H2,1-2H3,(H,25,32)(H,29,30)(H,33,34)/t12-,16?/m0/s1	JMSBAKPPKQAKDR-HKALDPMFSA-N
MF-3-GLA/ A6	2-[[7-[(4aS,7aS)-1,2,3,4,4a,5,7,7a-octahydropyrrolo[3,4-b]pyridin-6-yl]-1-cyclopropyl-6-fluoro-8-methoxy-4-oxo-quinoline-3-carbonyl]amino]pentanedioic acid	COc1c(N2C[C@@H]3CCCN[C@@H]3C2)c(F)cc4C(=O)C(=CN(C5CC5)c14)C(=O)NC(CCC(=O)O)C(=O)O	InChI=1S/C26H31FN4O7/c1-38-24-21-15(9-17(27)22(24)30-10-13-3-2-8-28-19(13)12-30)23(34)16(11-31(21)14-4-5-14)25(35)29-18(26(36)37)6-7-20(32)33/h9,11,13-14,18-19,28H,2-8,10,12H2,1H3,(H,29,35)(H,32,33)(H,36,37)/t13-,18?,19+/m0/s1	FFGBSFIDLYSAER-LPHKOSDUSA-N
CF-7-GLA/ B1	7-[4-(4-amino-4-carboxy-butanoyl)piperazin-1-yl]-1-cyclopropyl-6-fluoro-4-oxo-quinoline-3-carboxylic acid	NC(CCC(=O)N1CCN(CC1)c2cc3N(C=C(C(=O)O)C(=O)c3cc2F)C4CC4)C(=O)O	InChI=1S/C22H25FN4O6/c23-15-9-13-17(27(12-1-2-12)11-14(20(13)29)21(30)31)10-18(15)25-5-7-26(8-6-25)19(28)4-3-16(24)22(32)33/h9-12,16H,1-8,24H2,(H,30,31)(H,32,33)	YSADGWJFHIANTE-UHFFFAOYSA-N
NF-7-GLA/ B2	7-[4-(4-amino-4-carboxy-butanoyl)piperazin-1-yl]-1-ethyl-6-fluoro-4-oxo-quinoline-3-carboxylic acid	CCN1C=C(C(=O)O)C(=O)c2cc(F)c(cc12)N3CCN(CC3)C(=O)CCC(N)C(=O)O	InChI=1S/C21H25FN4O6/c1-2-24-11-13(20(29)30)19(28)12-9-14(22)17(10-16(12)24)25-5-7-26(8-6-25)18(27)4-3-15(23)21(31)32/h9-11,15H,2-8,23H2,1H3,(H,29,30)(H,31,32)	DIGVQXNKYIDFMO-UHFFFAOYSA-N
MF-7-GLA/ B6	7-[(4aS,7aS)-1-(4-amino-4-carboxy-butanoyl)-3,4,4a,5,7,7a-hexahydro-2H-pyrrolo[3,4-b]pyridin-6-yl]-1-cyclopropyl-6-fluoro-8-methoxy-4-oxo-quinoline-3-	COc1c(N2C[C@@H]3CCCN([C@@H]3C2)C(=O)CCC(N)C(=O)O)c(F)cc4C(=O)C(=CN(C5CC5)c14)C(=O)O	InChI=1S/C26H31FN4O7/c1-38-24-21-15(23(33)16(25(34)35)11-31(21)14-4-5-14)9-17(27)22(24)29-10-13-3-2-8-30(19(13)12-29)20(32)7-6-18(28)26(36)37/h9,11,13-14,18-19H,2-8,10,12,28H2,1H3,(H,34,35)(H,36,37)/t	KIIDBPHVPDXDHV-LPHKOSDUSA-N

Compound	IUPAC name	SMILES string	InChI string	Inchi key
	carboxylic acid		13-,18?,19+/m0/s1	
CF-3-GLA-HA/ C1	2-[(1-cyclopropyl-6-fluoro-4-oxo-7-piperazin-1-yl-quinoline-3-carbonyl)amino]-5-(hydroxyamino)-5-oxo-pentanoic acid	ONC(=O)CCC(NC(=O)C1=CN(C2CC2)c3cc(N4CCNCC4)c(F)cc3C1=O)C(=O)O	InChI=1S/C22H26FN5O6/c23-15-9-13-17(10-18(15)27-7-5-24-6-8-27)28(12-1-2-12)11-14(20(13)30)21(31)25-16(22(32)33)3-4-19(29)26-34/h9-12,16,24,34H,1-8H2,(H,25,31)(H,26,29)(H,32,33)	XPBAYILYLBYLSR-UHFFFAOYSA-N
NF-3-GLA-HA/ C2	2-[(1-ethyl-6-fluoro-4-oxo-7-piperazin-1-yl-quinoline-3-carbonyl)amino]-5-(hydroxyamino)-5-oxo-pentanoic acid	CCN1C=C(C(=O)NC(CCC(=O)NO)C(=O)O)C(=O)c2cc(F)c(cc12)N3CCNCC3	InChI=1S/C21H26FN5O6/c1-2-26-11-13(20(30)24-15(21(31)32)3-4-18(28)25-33)19(29)12-9-14(22)17(10-16(12)26)27-7-5-23-6-8-27/h9-11,15,23,33H,2-8H2,1H3,(H,24,30)(H,25,28)(H,31,32)	UPANOZLKYDFDRA-UHFFFAOYSA-N
PF-3-GLA-HA/ C3	2-[[1-ethyl-6-fluoro-7-(4-methylpiperazin-1-yl)-4-oxo-quinoline-3-carbonyl]amino]-5-(hydroxyamino)-5-oxo-pentanoic acid	CCN1C=C(C(=O)NC(CCC(=O)NO)C(=O)O)C(=O)c2cc(F)c(cc12)N3CCN(C)CC3	InChI=1S/C22H28FN5O6/c1-3-27-12-14(21(31)24-16(22(32)33)4-5-19(29)25-34)20(30)13-10-15(23)18(11-17(13)27)28-8-6-26(2)7-9-28/h10-12,16,34H,3-9H2,1-2H3,(H,24,31)(H,25,29)(H,32,33)	YGXPNHCFQFLCQY-UHFFFAOYSA-N
OF-3-GLA-HA/ C4	2-[[7-fluoro-2-methyl-6-(4-methylpiperazin-1-yl)-10-oxo-4-oxa-1-azatricyclo[7.3.1.05,13]trideca-5(13),6,8,11-tetraene-11-carbonyl]amino]-5-(hydroxyamino)-5-oxo-pentanoic acid	CC1COc2c(N3CCN(C)CC3)c(F)cc4C(=O)C(=CN1c24)C(=O)NC(CCC(=O)NO)C(=O)O	InChI=1S/C23H28FN5O7/c1-12-11-36-21-18-13(9-15(24)19(21)28-7-5-27(2)6-8-28)20(31)14(10-29(12)18)22(32)25-16(23(33)34)3-4-17(30)26-35/h9-10,12,16,35H,3-8,11H2,1-2H3,(H,25,32)(H,26,30)(H,33,34)	RQQDHDOXXVLGRY-UHFFFAOYSA-N
LF-3-GLA-HA/ C5	2-[[(2S)-7-fluoro-2-methyl-6-(4-methylpiperazin-1-yl)-10-oxo-4-oxa-1-azatricyclo[7.3.1.05,13]trideca-5(13),6,8,11-tetraene-11-carbonyl]amino]-5-(hydroxyamino)-5-oxo-pentanoic acid	C[C@H]1COc2c(N3CCN(C)CC3)c(F)cc4C(=O)C(=CN1c24)C(=O)NC(CCC(=O)NO)C(=O)O	InChI=1S/C23H28FN5O7/c1-12-11-36-21-18-13(9-15(24)19(21)28-7-5-27(2)6-8-28)20(31)14(10-29(12)18)22(32)25-16(23(33)34)3-4-17(30)26-35/h9-10,12,16,35H,3-8,11H2,1-2H3,(H,25,32)(H,26,30)(H,33,34)/t12-,16?/m0/s1	RQQDHDOXXVLGRY-HKALDPMFSA-N
MF-3-GLA-HA/ C6	2-[[7-[(4aS,7aS)-1,2,3,4,4a,5,7,7a-octahydropyrrolo[3,4-b]pyridin-6-yl]-1-cyclopropyl-6-fluoro-8-methoxy-4-oxo-quinoline-3-carbonyl]amino]-5-(hydroxyamino)-5-oxo-pentanoic acid	COc1c(N2C[C@@H]3CCCN[C@@H]3C2)c(F)cc4C(=O)C(=CN(C5CC5)c14)C(=O)NC(CCC(=O)NO)C(=O)O	InChI=1S/C26H32FN5O7/c1-39-24-21-15(9-17(27)22(24)31-10-13-3-2-8-28-19(13)12-31)23(34)16(11-32(21)14-4-5-14)25(35)29-18(26(36)37)6-7-20(33)30-38/h9,11,13-14,18-19,28,38H,2-8,10,12H2,1H3,(H,29,35)(H,30,33)(H,36,37)/t13-,18?,19+/m0/s1	UCYQGRDDGBHSMO-LPHKOSDUSA-N
CF-3-EA-GLA/ D1	2-amino-5-[2-[(1-cyclopropyl-6-fluoro-4-oxo-7-piperazin-1-yl-quinoline-3-carbonyl)amino]ethoxy]-5-oxo-pentanoic acid	NC(CCC(=O)OCCNC(=O)C1=CN(C2CC2)c3cc(N4CCNCC4)c(F)cc3C1=O)C(=O)O	InChI=1S/C24H30FN5O6/c25-17-11-15-19(12-20(17)29-8-5-27-6-9-29)30(14-1-2-14)13-16(22(15)32)23(33)28-7-10-36-21(31)4-3-18(26)24(34)35/h11-14,18,27H,1-10,26H2,(H,28,33)(H,34,35)	HOWCMCSTAACKRP-UHFFFAOYSA-N
NF-3-EA-	2-amino-5-[2-[(1-ethyl-6-	CCN1C=C(C(=O)	InChI=1S/C23H30FN5O6/c1-2-28-13-	IPMFUAW

Compound	IUPAC name	SMILES string	InChI string	Inchi key
GLA/ D2	fluoro-4-oxo-7-piperazin-1-yl-quinoline-3-carbonyl)amino]ethoxy]-5-oxo-pentanoic acid	NCCOC(=O)CCC(N)C(=O)O)C(=O)c2cc(F)c(cc12)N3CCNCC3	15(22(32)27-7-10-35-20(30)4-3-17(25)23(33)34)21(31)14-11-16(24)19(12-18(14)28)29-8-5-26-6-9-29/h11-13,17,26H,2-10,25H2,1H3,(H,27,32)(H,33,34)	URUKIRQ-UHFFFAOYSA-N
PF-3-EA-GLA/ D3	2-amino-5-[2-[[1-ethyl-6-fluoro-7-(4-methylpiperazin-1-yl)-4-oxo-quinoline-3-carbonyl]amino]ethoxy]-5-oxo-pentanoic acid	CCN1C=C(C(=O)NCCOC(=O)CCC(N)C(=O)O)C(=O)c2cc(F)c(cc12)N3CCN(C)CC3	InChI=1S/C24H32FN5O6/c1-3-29-14-16(23(33)27-6-11-36-21(31)5-4-18(26)24(34)35)22(32)15-12-17(25)20(13-19(15)29)30-9-7-28(2)8-10-30/h12-14,18H,3-11,26H2,1-2H3,(H,27,33)(H,34,35)	FGUBFTWBOXSVRL-UHFFFAOYSA-N
OF-3-EA-GLA/ D4	2-amino-5-[2-[[7-fluoro-2-methyl-6-(4-methylpiperazin-1-yl)-10-oxo-4-oxa-1-azatricyclo[7.3.1.05,13]trideca-5(13),6,8,11-tetraene-11-carbonyl]amino]ethoxy]-5-oxo-pentanoic acid	CC1COc2c(N3CCN(C)CC3)c(F)cc4C(=O)C(=CN1c24)C(=O)NCCOC(=O)CCC(N)C(=O)O	InChI=1S/C25H32FN5O7/c1-14-13-38-23-20-15(11-17(26)21(23)30-8-6-29(2)7-9-30)22(33)16(12-31(14)20)24(34)28-5-10-37-19(32)4-3-18(27)25(35)36/h11-12,14,18H,3-10,13,27H2,1-2H3,(H,28,34)(H,35,36)	UNEYJNQRIUTDTH-UHFFFAOYSA-N
LF-3-EA-GLA/ D5	2-amino-5-[2-[[(2S)-7-fluoro-2-methyl-6-(4-methylpiperazin-1-yl)-10-oxo-4-oxa-1-azatricyclo[7.3.1.05,13]trideca-5(13),6,8,11-tetraene-11-carbonyl]amino]ethoxy]-5-oxo-pentanoic acid	C[C@H]1COc2c(N3CCN(C)CC3)c(F)cc4C(=O)C(=CN1c24)C(=O)NCCOC(=O)CCC(N)C(=O)O	InChI=1S/C25H32FN5O7/c1-14-13-38-23-20-15(11-17(26)21(23)30-8-6-29(2)7-9-30)22(33)16(12-31(14)20)24(34)28-5-10-37-19(32)4-3-18(27)25(35)36/h11-12,14,18H,3-10,13,27H2,1-2H3,(H,28,34)(H,35,36)/t14-,18?/m0/s1	UNEYJNQRIUTDTH-PIVQAISJSA-N
MF-3-EA-GLA/ D6	5-[2-[[7-[(4aS,7aS)-1,2,3,4,4a,5,7,7a-octahydropyrrolo[3,4-b]pyridin-6-yl]-1-cyclopropyl-6-fluoro-8-methoxy-4-oxo-quinoline-3-carbonyl]amino]ethoxy]-2-amino-5-oxo-pentanoic acid	COc1c(N2C[C@@H]3CCCN[C@@H]3C2)c(F)cc4C(=O)C(=CN(C5CC5)c14)C(=O)NCCOC(=O)CCC(N)C(=O)O	InChI=1S/C28H36FN5O7/c1-40-26-23-17(11-19(29)24(26)33-12-15-3-2-8-31-21(15)14-33)25(36)18(13-34(23)16-4-5-16)27(37)32-9-10-41-22(35)7-6-20(30)28(38)39/h11,13,15-16,20-21,31H,2-10,12,14,30H2,1H3,(H,32,37)(H,38,39)/t15-,20?,21+/m0/s1	WZLOPZIACNNLET-GMTNLFQVSA-N
CF-3-EDA-GLA/ E1	2-amino-5-[2-[(1-cyclopropyl-6-fluoro-4-oxo-7-piperazin-1-yl-quinoline-3-carbonyl)amino]ethylamino]-5-oxo-pentanoic acid	NC(CCC(=O)NCCNC(=O)C1=CN(C2CC2)c3cc(N4CCNCC4)c(F)cc3C1=O)C(=O)O	InChI=1S/C24H31FN6O5/c25-17-11-15-19(12-20(17)30-9-7-27-8-10-30)31(14-1-2-14)13-16(22(15)33)23(34)29-6-5-28-21(32)4-3-18(26)24(35)36/h11-14,18,27H,1-10,26H2,(H,28,32)(H,29,34)(H,35,36)	MUPJFYHYBLQFNK-UHFFFAOYSA-N
NF-3-EDA-GLA/E2	2-amino-5-[2-[(1-ethyl-6-fluoro-4-oxo-7-piperazin-1-yl-quinoline-3-carbonyl)amino]ethylamino]-5-oxo-pentanoic acid	CCN1C=C(C(=O)NCCNC(=O)CCC(N)C(=O)O)C(=O)c2cc(F)c(cc12)N3CCNCC3	InChI=1S/C23H31FN6O5/c1-2-29-13-15(22(33)28-6-5-27-20(31)4-3-17(25)23(34)35)21(32)14-11-16(24)19(12-18(14)29)30-9-7-26-8-10-30/h11-13,17,26H,2-10,25H2,1H3,(H,27,31)(H,28,33)(H,34,35)	UGGMCAGJFDCBJV-UHFFFAOYSA-N
PF-3-EDA-GLA/ E3	2-amino-5-[2-[[1-ethyl-6-fluoro-7-(4-methylpiperazin-1-yl)-4-oxo-quinoline-3-	CCN1C=C(C(=O)NCCNC(=O)CCC(N)C(=O)O)C(=O)	InChI=1S/C24H33FN6O5/c1-3-30-14-16(23(34)28-7-6-27-21(32)5-4-18(26)24(35)36)22(33)15-12-	XGNJLYXLTNBPJF-UHFFFAO

Compound	IUPAC name	SMILES string	InChI string	Inchi key
	carbonyl]amino]ethylamino]-5-oxo-pentanoic acid	c2cc(F)c(cc12)N3CCN(C)CC3	17(25)20(13-19(15)30)31-10-8-29(2)9-11-31/h12-14,18H,3-11,26H2,1-2H3,(H,27,32)(H,28,34)(H,35,36)	YSA-N
OF-3-EDA-GLA/ E4	2-amino-5-[2-[[7-fluoro-2-methyl-6-(4-methylpiperazin-1-yl)-10-oxo-4-oxa-1-azatricyclo[7.3.1.05,13]trideca-5(13),6,8,11-tetraene-11-carbonyl]amino]ethylamino]-5-oxo-pentanoic acid	CC1COc2c(N3CCN(C)CC3)c(F)cc4C(=O)C(=CN1c24)C(=O)NCCNC(=O)CCC(N)C(=O)O	InChI=1S/C25H33FN6O6/c1-14-13-38-23-20-15(11-17(26)21(23)31-9-7-30(2)8-10-31)22(34)16(12-32(14)20)24(35)29-6-5-28-19(33)4-3-18(27)25(36)37/h11-12,14,18H,3-10,13,27H2,1-2H3,(H,28,33)(H,29,35)(H,36,37)	ORAPQEPCGJMYLU-UHFFFAOYSA-N
LF-3-EDA-GLA/ E5	2-amino-5-[2-[[(2S)-7-fluoro-2-methyl-6-(4-methylpiperazin-1-yl)-10-oxo-4-oxa-1-azatricyclo[7.3.1.05,13]trideca-5(13),6,8,11-tetraene-11-carbonyl]amino]ethylamino]-5-oxo-pentanoic acid	C[C@H]1COc2c(N3CCN(C)CC3)c(F)cc4C(=O)C(=CN1c24)C(=O)NCCNC(=O)CCC(N)C(=O)O	InChI=1S/C25H33FN6O6/c1-14-13-38-23-20-15(11-17(26)21(23)31-9-7-30(2)8-10-31)22(34)16(12-32(14)20)24(35)29-6-5-28-19(33)4-3-18(27)25(36)37/h11-12,14,18H,3-10,13,27H2,1-2H3,(H,28,33)(H,29,35)(H,36,37)/t14-,18?/m0/s1	ORAPQEPCGJMYLU-PIVQAISJSA-N
MF-3-EDA-GLA/ E6	5-[2-[[7-[(4aS,7aS)-1,2,3,4,4a,5,7,7a-octahydropyrrolo[3,4-b]pyridin-6-yl]-1-cyclopropyl-6-fluoro-8-methoxy-4-oxo-quinoline-3-carbonyl]amino]ethylamino]-2-amino-5-oxo-pentanoic acid	COc1c(N2C[C@@H]3CCCN[C@@H]3C2)c(F)cc4C(=O)C(=CN(C5CC5)c14)C(=O)NCCNC(=O)CCC(N)C(=O)O	InChI=1S/C28H37FN6O6/c1-41-26-23-17(11-19(29)24(26)34-12-15-3-2-8-31-21(15)14-34)25(37)18(13-35(23)16-4-5-16)27(38)33-10-9-32-22(36)7-6-20(30)28(39)40/h11,13,15-16,20-21,31H,2-10,12,14,30H2,1H3,(H,32,36)(H,33,38)(H,39,40)/t15-,20?,21+/m0/s1	JRVLOYCVLHYMIZ-GMTNLFQVSA-N
Etoposide	(5*S*,5*aR*,8*aR*,9*R*)-5-[[(2*R*,4*aR*,6*R*,7*R*,8*R*,8*aS*)-7,8-dihydroxy-2-methyl-4,4*a*,6,7,8,8*a*-hexahydropyrano[3,2-d][1,3]dioxin-6-yl]oxy]-9-(4-hydroxy-3,5-dimethoxyphenyl)-5*a*,6,8*a*,9-tetrahydro-5*H*-[2]benzofuro[6,5-f][1,3]benzodioxol-8-one	C[C@@H]1OC[C@@H]2[C@@H](O1)[C@@H]([C@H]([C@@H](O2)O[C@H]3[C@H]4COC(=O)[C@@H]4[C@@H](C5=CC6=C(C=C35)OCO6)C7=CC(=C(C(=C7)OC)O)OC)O)O	InChI=1S/C29H32O13/c1-11-36-9-20-27(40-11)24(31)25(32)29(41-20)42-26-14-7-17-16(38-10-39-17)6-13(14)21(22-15(26)8-37-28(22)33)12-4-18(34-2)23(30)19(5-12)35-3/h4-7,11,15,20-22,24-27,29-32H,8-10H2,1-3H3/t11-,15+,20-,21-,22+,24-,25-,26-,27-,29+/m1/s1	VJJPUSNTGOMMGY-MRVIYFEKSA-N

Water solubility.

SwissADME uses three methods to predict water solubility. The first one is implemented after Delaney JS. (2004) [38] (ESOL), and the second one is taken from Ali J. et al. (2012) [39], both being topological methods. Even if they use a different general solubility equation, both exclude the melting point and show a linear correlation between the predicted and the experimental values (R^2=0,69, respectively 0,81). Another method was developed by SILICOS-IT (fragmentary method calculated using FILTER-IT 1.0.2 program). In this care, the correlation coefficient is corrected by the molecular weight and (R^2=0.75). All three methods calculate the decimal logarithm of molar water solubility (logS), but the results can be expressed in mol/l şi mg/ml. In each case, the solubility class follows the

same logS scale: Insoluble (I) <-10< Poorly soluble (P) <-6< Moderately soluble (M) <-4< Soluble (S) <-2< Very soluble (V) <0< Highly soluble (H) [40].

Table 5. Water solubility

Compound	logS Aqua Sol	ESOL Log S	ESOL Solub. (mg/ml)	ESOL Solub. (mol/l)	ESOL Class	Ali Log S	Ali Sol (mg/ml)	Ali Sol (mol/l)	Ali Class	Silicos-IT LogSw	Silicos-IT Sol. (mg/ml)	Silicos-IT Sol. (mol/l)	Silicos-IT class
CF-3-GLA/ A1	-3.16	-1.16	31.9	0.0693	V	-0.59	118	0.256	V	-3.63	0.108	0.000235	S
NF-3-GLA/ A2	-3.16	-1.12	33.7	0.0752	V	-0.64	102	0.227	V	-3.83	0.0657	0.000147	S
PF-3-GLA/ A3	-3.13	-2.02	4.39	0.00949	S	-1.81	7.19	0.0156	V	-3.49	0.149	0.000322	S
OF-3-GLA/ A4	-3.07	-1.83	7.19	0.0147	V	-1.32	23.6	0.0482	V	-3.12	0.374	0.000762	S
LF-3-GLA/ A5	-3.07	-1.83	7.19	0.0147	V	-1.32	23.6	0.0482	V	-3.12	0.374	0.000762	S
MF-3-GLA/ A6	-3.32	2.56	1.45	0.00273	S	-2.54	1.53	0.00289	S	-0.45	0.0476	0.0000897	M
CF-7-GLA/ B1	-3.27	-1.02	43.6	0.0946	V	-0.37	197	0.428	V	-2.5	1.45	0.00314	S
NF-7-GLA/ B2	-3.07	-0.99	46.1	0.103	V	-0.42	170	0.379	V	-2.71	0.878	0.00196	S
MF-7-GLA/ B6	-3.26	-2.42	2.01	0.00379	S	-2.31	2.62	0.00494	S	-2.92	0.635	0.0012	S
CF-3-GLA-HA/ C1	-3.29	-0.72	90.6	0.19	V	-0.09	389	0.819	V	-3.67	0.103	0.000216	S
NF-3-GLA-HA/ C2	-3.33	-0.68	96.0	0.207	V	-0.14	337	0.726	V	-3.87	0.0624	0.000135	S
PF-3-GLA-HA/ C3	-3.30	-1.58	12.5	0.0261	V	-1.30	23.8	0.0497	V	-3.53	0.142	0.000296	S
OF-3-GLA-HA/ C4	-3.25	-1.39	20.4	0.00403	V	-0.81	77.9	0.154	V	-3.15	0.355	0.000703	S
LF-3-GLA-HA/ C5	-3.25	-1.39	20.4	0.00403	V	-0.81	77.9	0.154	V	-3.15	0.355	0.000703	S
MF-3-GLA-HA/ C6	-3.45	-2.12	4.15	0.0076	S	-2.02	5.16	0.00946	S	-4.08	0.0453	0.000083	M
CF-3-EA-GLA/ D1	-3.28	-0.83	75.2	0.149	V	-0.27	268	0.532	V	-4.35	0.0225	0.0000448	M
NF-3-EA-GLA/ D2	-3.20	-0.79	80.0	0.163	V	-0.33	232	0.472	V	-4.55	0.0137	0.0000279	M
PF-3-EA-GLA/ D3	-3.18	-1.69	10.4	0.0205	V	-1.49	16.3	0.0323	V	-4.21	0.0311	0.0000615	M
OF-3-EA-GLA/ D4	-3.13	-1.50	16.8	0.0315	V	-1.0	53.4	0.1	V	-3.83	0.0782	0.000147	S
LF-3-EA-GLA/ D5	-3.13	-1.50	16.8	0.0315	V	-1.0	53.4	0.1	V	-3.83	0.0782	0.000147	S
MF-3-EA-GLA/ D6	-3.34	-2.23	3.35	0.00584	S	-2.22	3.44	0.006	S	-4.76	0.00997	0.0000174	M
CF-3-EDA-GLA/ E1	-3.34	-0.46	174	0.346	V	0.26	911	1.81	H	-4.66	0.011	0.0000218	M
NF-3-EDA-GLA/ E2	-3.23	-0.42	185	0.377	V	0.21	789	1.61	H	-4.87	0.00666	0.0000136	M
PF-3-EDA-GLA/ E3	-3.21	-1.32	24.0	0.0475	V	-0.96	55.6	0.11	V	-4.52	0.0151	0.00003	M
OF-3-EDA-GLA/ E4	-3.16	-1.14	38.9	0.073	V	-0.47	182	0.341	V	-4.15	0.038	0.0000713	M

Compound	logS Aqua Sol	ESOL Log S	ESOL Solub. (mg/ml)	ESOL Solub. (mol/l)	ESOL Class	Ali Log S	Ali Sol (mg/ml)	Ali Sol (mol/l)	Ali Class	Silicos-IT LogSw	Silicos-IT Sol. (mg/ml)	Silicos-IT Sol. (mol/l)	Silicos-IT class
LF-3-EDA-GLA/ E5	-3.16	-1.14	38.9	0.073	V	-0.47	182	0.341	V	-4.15	0.038	0.0000713	M
MF-3-EDA-GLA/ E6	-3.36	-1.86	7.86	0.0137	V	-1.68	12	0.021	V	-5.07	0.00485	0.00000847	M
Etoposide	-3.26	-3.75	0.105	0.000178	S	-3.55	0.165	0.000281	S	-3.18	0.385	0.000655	S

Compound/ID	pKa score	pI	Molar polarizability	*Conformations Emin (kcal/mol)
CF-3-GLA/A1	8.69	4.56	43.06	207.64
NF-3-GLA/A2	8.69	4.57	41.94	81.36
PF-3-GLA/A3	7.30	4.57	43.81	86.64
OF-3-GLA/A4	7.01	4.47	45.66	98.16
LF-3-GLA/A5	7.01	4.47	45.66	101.23
MF-3-GLA/A6	9.43	4.46	50.38	243.03

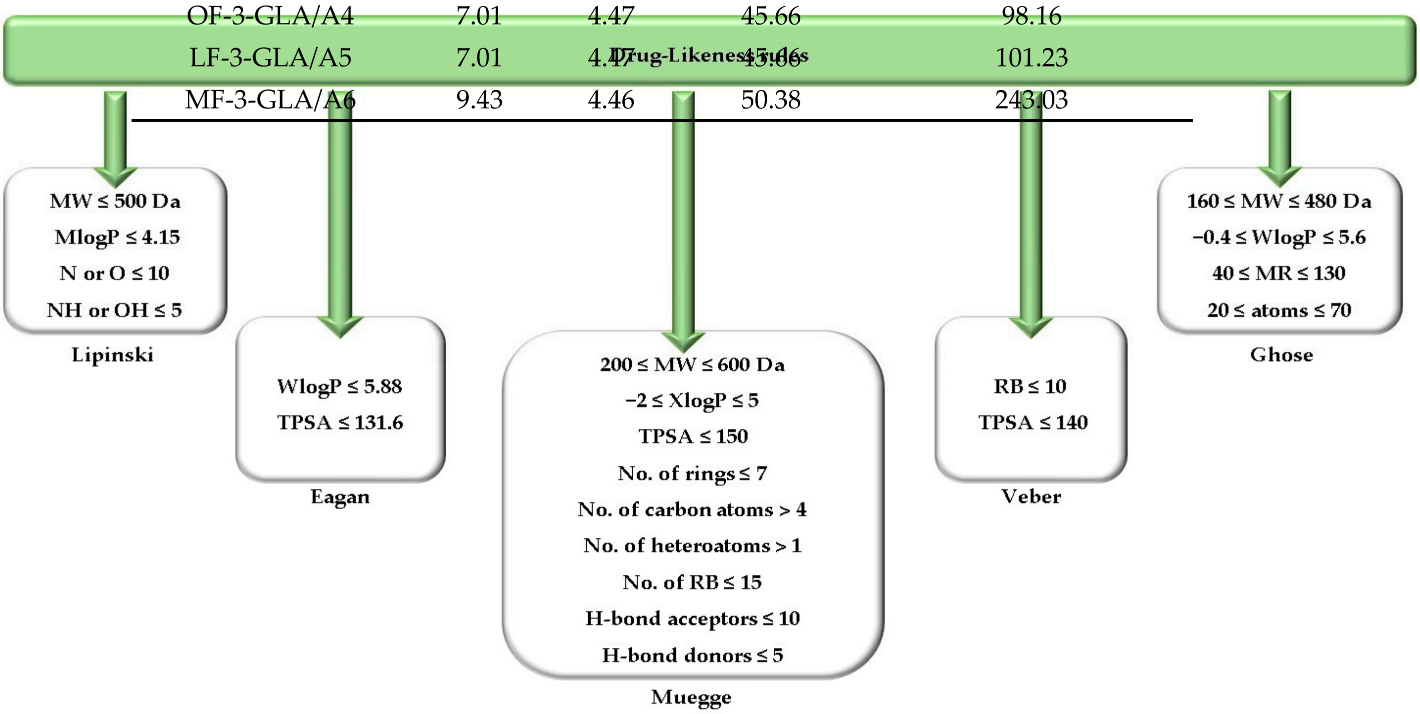

Figure S1. The characteristics of Lipinski, Ghose, Veber, Egan, and Muegge drug-likeness rules according to SwissAdme

Table S6. Prediction of pKa score, pI, molar polarizability, and the best conformation for each hybrid (MarvinSketch)

CF-7-GLA/B1	9.31	4.62	43.06	212.88
NF-7-GLA/B2	9.31	4.62	41.94	90.36
MF-7-GLA/B6	9.31	4.64	50.38	257.27
CF-3-GLA-HA/C1	8.61	6.34	44.51	208.15
NF-3-GLA-HA/C2	9.48	6.35	43.39	83.10
PF-3-GLA-HA/C3	7.3	5.71	45.26	88.50
OF-3-GLA-HA/C4	7.01	5.50	47.11	102.05
LF-3-GLA-HA/C5	7.01	5.50	47.11	104.83
MF-3-GLA-HA/C6	9.72	6.49	51.82	257.07
CF-3-EA-GLA/D1	9.57	9.07	48.54	221.47
NF-3-EA-GLA/D2	9.57	9.07	47.42	87.38
PF-3-EA-GLA/D3	9.51	8.41	49.29	93.89
OF-3-EA-GLA/D4	9.51	8.26	51.14	106.88
LF-3-EA-GLA/D5	9.51	8.26	51.14	116.86
MF-3-EA-GLA/D6	9.77	9.34	55.86	273.82
CF-3-EDA-GLA/E1	9.40	8.95	48.95	212.85
NF-3-EDA-GLA/E2	9.40	8.95	47.84	103.12
PF-3-EDA-GLA/E3	9.32	8.31	49.71	98.46
OF-3-EDA-GLA/E4	9.31	8.16	51.56	109.23
LF-3-EDA-GLA/E5	9.31	8.16	51.56	110.30
MF-3-EDA-GLA/E6	9.68	9.37	56.28	287.04
Etoposide	-3.69	-	55.39	146.21

*Assessed using Dreiding Forcefield

Table S7. Mechanisms of action and adverse/toxic effects - Probability to be active (Pa) > Probability to be inactive (Pi) (Pass Online platform).

**Compound/ID**	**Mechanism of action**			**Toxic effects**		
	**Pa**	**Pi**	**Activity**	**Pa**	**Pi**	**Activity**
CF-3-GLA/A1	0.631	0.008	DNA synthesis inhibitor	0.917	0.001	Bullous pemphigoid
	0.561	0.002	Antibacterial. ophthalmic	0.916	0.008	Stomatitis
	0.566	0.016	Antiinfective	0.907	0.003	Allergic contact dermatitis
	0.576	0.140	Gluconate 2-dehydrogenase (acceptor) inhibitor	0.834	0.005	Myoclonus
	0.466	0.052	Erythropoiesis stimulant	0.842	0.014	Necrosis

Compound/ID	Mechanism of action			Toxic effects		
	Pa	Pi	Activity	Pa	Pi	Activity
	0.413	0.006	*Topoisomerase II inhibitor			
NF-3-GLA/A2	0.721	0.012	Proteasome ATPase inhibitor	0.931	0.003	Allergic contact dermatitis
	0.610	0.012	Antiinfective	0.878	0.004	Nail discoloration
	0.595	0.010	DNA synthesis inhibitor	0.881	0.012	Stomatitis
	0.551	0.027	Methylamine-glutamate N-methyltransferase inhibitor	0.863	0.002	Photoallergy dermatitis
	0.519	0.002	Antibacterial. ophthalmic	0.835	0.005	Myoclonus
	0.304	0.010	*Topoisomerase II inhibitor			
PF-3-GLA/A3	0.696	0.015	Proteasome ATPase inhibitor	0.929	0.002	Nail discoloration
	0.578	0.011	DNA synthesis inhibitor	0.915	0.003	Allergic contact dermatitis
	0.539	0.019	Antiinfective	0.884	0.011	Stomatitis
	0.531	0.030	Methylamine-glutamate N-methyltransferase inhibitor	0.861	0.004	Myoclonus
	0.454	0.002	Antibacterial. ophthalmic	0.850	0.003	Photoallergy dermatitis
	0.296	0.011	*Topoisomerase II inhibitor			
OF-3-GLA/A4	0.750	0.027	Nootropic	0.946	0.004	Stomatitis
	0.656	0.008	DNA synthesis inhibitor	0.933	0.003	Allergic contact dermatitis
	0.574	0.002	Antibacterial. ophthalmic	0.920	0.004	Delirium
	0.492	0.004	*Topoisomerase II inhibitor	0.916	0.006	Asthma
	0.496	0.015	P-glycoprotein substrate	0.913	0.005	Urinary retention
LF-3-GLA/A5	0.750	0.027	Nootropic	0.946	0.004	Stomatitis
	0.656	0.008	DNA synthesis inhibitor	0.933	0.003	Allergic contact dermatitis
	0.574	0.002	Antibacterial. ophthalmic	0.920	0.004	Delirium
	0.492	0.004	*Topoisomerase II inhibitor	0.916	0.006	Asthma
	0.496	0.015	P-glycoprotein substrate	0.913	0.005	Urinary retention
MF-3-GLA/A6	0.651	0.008	DNA synthesis inhibitor	0.943	0.005	Stomatitis
	0.613	0.003	RELA expression inhibitor	0.941	0.004	Hypotonia
	0.635	0.032	Nicotinic alpha4beta4 receptor agonist	0.925	0.005	Asthma
	0.517	0.004	*Topoisomerase II inhibitor	0.895	0.004	Bradycardic
	0.511	0.015	Antibacterial	0.882	0.006	Urinary retention

Compound/ID	Mechanism of action			Toxic effects		
	Pa	Pi	Activity	Pa	Pi	Activity
CF-7-GLA/B1	0.689	0.001	Antibacterial. ophthalmic	0.947	0.001	Bullous pemphigoid
	0.679	0.030	Glutamate-5-semialdehyde dehydrogenase inhibitor	0.923	0.003	Allergic contact dermatitis
	0.630	0.011	Antiinfective	0.902	0.002	Photoallergy dermatitis
	0.619	0.009	DNA synthesis inhibitor	0.877	0.005	Delirium
	0.618	0.012	Aspartyltransferase inhibitor	0.858	0.014	Stomatitis
	0.505	0.004	*Topoisomerase II inhibitor	0.896	0.010	Stomatitis
NF-7-GLA/B2	0.753	0.010	Muramoyltetrapeptide carboxypeptidase inhibitor	0.948	0.002	Nail discoloration
	0.687	0.016	Proteasome ATPase inhibitor	0.941	0.002	Allergic contact dermatitis
	0.674	0.009	Antiinfective	0.921	0.001	Photoallergy dermatitis
	0.679	0.017	Dimethylargininase inhibitor	0.839	0.003	Bullous pemphigoid
	0.648	0.001	Antibacterial. ophthalmic	0.828	0.007	Delirium
	0.405	0.006	*Topoisomerase II inhibitor			
MF-7-GLA/B6	0.686	0.045	Nootropic	0.858	0.009	Asthma
	0.608	0.008	Antibacterial	0.801	0.012	Hypotonia
	0.580	0.004	*Topoisomerase II inhibitor	0.786	0.013	Urinary retention
	0.577	0.011	DNA synthesis inhibitor	0.782	0.012	Bradycardic
	0.576	0.045	Glutamate-5-semialdehyde dehydrogenase inhibitor	0.896	0.010	Stomatitis
CF-3-GLA-HA/C1	0.475	0.020	DNA synthesis inhibitor	0.832	0.003	Bullous pemphigoid
	0.460	0.058	Antiarthritic	0.832	0.004	Allergic contact dermatitis
	0.382	0.003	Antibacterial. ophthalmic	0.624	0.010	Photoallergy dermatitis
	0.344	0.010	Antineoplastic (sarcoma)	0.604	0.033	Hematuria
	0.333	0.009	*Topoisomerase II inhibitor	0.607	0.040	Necrosis
NF-3-GLA-HA/C2	0.677	0.018	Proteasome ATPase inhibitor	0.875	0.004	Allergic contact dermatitis
	0.507	0.040	Muramoyltetrapeptide carboxypeptidase inhibitor	0.719	0.027	Nail discoloration
	0.489	0.051	Antiarthritic	0.669	0.005	Photoallergy dermatitis
	0.435	0.024	DNA synthesis inhibitor	0.550	0.016	Bullous pemphigoid

Compound/ID	Mechanism of action			Toxic effects		
	Pa	Pi	Activity	Pa	Pi	Activity
	0.352	0.003	Antibacterial. ophthalmic	0.580	0.047	Necrosis
	0.226	0.016	*Topoisomerase II inhibitor			
PF-3-GLA-HA/ C3	0.651	0.022	Proteasome ATPase inhibitor	0.845	0.004	Allergic contact dermatitis
	0.475	0.047	Muramoyltetrapeptide carboxypeptidase inhibitor	0.833	0.007	Nail discoloration
	0.411	0.026	DNA synthesis inhibitor	0.653	0.007	Photoallergy dermatitis
	0.442	0.064	Antiarthritic	0.517	0.066	Necrosis
	0.323	0.014	Antineoplastic (sarcoma)	0.488	0.084	Urinary retention
	0.218	0.017	*Topoisomerase II inhibitor	0.845	0.004	Allergic contact dermatitis
OF-3-GLA-HA/ C4	0.494	0.019	DNA synthesis inhibitor	0.880	0.004	Allergic contact dermatitis
	0.470	0.013	Antineoplastic (multiple myeloma)	0.773	0.015	Urinary retention
	0.459	0.022	P-glycoprotein substrate	0.735	0.025	Stomatitis
	0.420	0.005	*Topoisomerase II inhibitor	0.729	0.022	Hyperglycemic
	0.430	0.018	Prostate cancer treatment	0.729	0.023	Necrosis
LF-3-GLA-HA/ C5	0.494	0.019	DNA synthesis inhibitor	0.880	0.004	Allergic contact dermatitis
	0.470	0.013	Antineoplastic (multiple myeloma)	0.773	0.015	Urinary retention
	0.459	0.022	P-glycoprotein substrate	0.735	0.025	Stomatitis
	0.420	0.005	*Topoisomerase II inhibitor	0.729	0.022	Hyperglycemic
	0.430	0.018	Prostate cancer treatment	0.729	0.023	Necrosis
MF-3-GLA-HA/ C6	0.572	0.050	Nicotinic alpha4beta4 receptor agonist	0.736	0.020	Urinary retention
	0.489	0.012	RELA expression inhibitor	0.704	0.028	Stomatitis
	0.491	0.019	DNA synthesis inhibitor	0.675	0.024	Thrombophlebitis
	0.469	0.019	Antibacterial	0.638	0.030	Asthma
	0.448	0.005	*Topoisomerase II inhibitor	0.561	0.020	Torsades de pointes
CF-3-EA-GLA/ D1	0.586	0.011	DNA synthesis inhibitor	0.903	0.003	Allergic contact dermatitis
	0.515	0.027	Aspartyltransferase inhibitor	0.882	0.009	Necrosis
	0.481	0.025	Antiinfective	0.863	0.002	Bullous pemphigoid
	0.503	0.060	Glutamate-5-semialdehyde dehydrogenase inhibitor	0.825	0.008	Bradycardic
	0.420	0.026	Antibacterial	0.810	0.008	Myoclonus

Compound/ID	Mechanism of action			Toxic effects		
	Pa	Pi	Activity	Pa	Pi	Activity
	0.370	0.007	*Topoisomerase II inhibitor			
NF-3-EA-GLA/ D2	0.661	0.020	Proteasome ATPase inhibitor	0.929	0.003	Allergic contact dermatitis
	0.636	0.021	Muramoyltetrapeptide carboxypeptidase inhibitor	0.876	0.010	Necrosis
	0.560	0.013	DNA synthesis inhibitor	0.810	0.003	Photoallergy dermatitis
	0.533	0.019	Antiinfective	0.811	0.008	Myoclonus
	0.512	0.028	Aspartyltransferase inhibitor	0.781	0.028	Diarrhea
	0.279	0.012	*Topoisomerase II inhibitor			
PF-3-EA-GLA/ D3	0.635	0.026	Proteasome ATPase inhibitor	0.912	0.003	Allergic contact dermatitis
	0.608	0.024	Muramoyltetrapeptide carboxypeptidase inhibitor	0.857	0.012	Necrosis
	0.545	0.014	DNA synthesis inhibitor	0.840	0.005	Myoclonus
	0.507	0.029	Aspartyltransferase inhibitor	0.805	0.010	Nail discoloration
	0.458	0.029	Antiinfective	0.800	0.009	Delirium
	0.272	0.013	*Topoisomerase II inhibitor			
OF-3-EA-GLA/ D4	0.775	0.022	Nootropic	0.930	0.003	Allergic contact dermatitis
	0.596	0.010	DNA synthesis inhibitor	0.921	0.004	Bradycardic
	0.496	0.032	Aspartyltransferase inhibitor	0.915	0.005	Ataxia
	0.481	0.018	Antibacterial	0.916	0.007	Xerostomia
	0.455	0.005	*Topoisomerase II inhibitor	0.910	0.004	Delirium
LF-3-EA-GLA/ D5	0.775	0.022	Nootropic	0.930	0.003	Allergic contact dermatitis
	0.596	0.010	DNA synthesis inhibitor	0.921	0.004	Bradycardic
	0.496	0.032	Aspartyltransferase inhibitor	0.915	0.005	Ataxia
	0.481	0.018	Antibacterial	0.916	0.007	Xerostomia
	0.455	0.005	*Topoisomerase II inhibitor	0.910	0.004	Delirium
MF-3-EA-GLA/ D6	0.600	0.010	DNA synthesis inhibitor	0.967	0.003	Hypotonia
	0.521	0.015	Antibacterial	0.933	0.005	Hypertensive
	0.555	0.056	Nicotinic alpha4beta4 receptor agonist	0.931	0.004	Bradycardic
	0.478	0.005	*Topoisomerase II inhibitor	0.908	0.009	Stomatitis

Compound/ID	Mechanism of action			Toxic effects		
	Pa	Pi	Activity	Pa	Pi	Activity
	0.471	0.024	Antimycobacterial	0.886	0.007	Asthma
CF-3-EDA-GLA/ E1	0.597	0.041	Glutamate-5-semialdehyde dehydrogenase inhibitor	0.909	0.001	Bullous pemphigoid
	0.541	0.002	Antibacterial. ophthalmic	0.844	0.004	Allergic contact dermatitis
	0.544	0.014	DNA synthesis inhibitor	0.807	0.004	Psychoses
	0.524	0.025	Aspartyltransferase inhibitor	0.768	0.021	Stomatitis
	0.473	0.026	Antiinfective	0.748	0.014	Myoclonus
	0.391	0.007	*Topoisomerase II inhibitor	0.901	0.005	Hypotonia
NF-3-EDA-GLA/ E2	0.695	0.015	Proteasome ATPase inhibitor	0.885	0.004	Allergic contact dermatitis
	0.672	0.017	Muramoyltetrapeptide carboxypeptidase inhibitor	0.765	0.004	Photoallergy dermatitis
	0.524	0.020	Antiinfective	0.769	0.016	Nail discoloration
	0.519	0.016	DNA synthesis inhibitor	0.749	0.014	Myoclonus
	0.500	0.002	Antibacterial. ophthalmic	0.738	0.004	Bullous pemphigoid
	0.295	0.011	*Topoisomerase II inhibitor			
PF-3-EDA-GLA/ E3	0.671	0.019	Proteasome ATPase inhibitor	0.856	0.004	Allergic contact dermatitis
	0.644	0.020	Muramoyltetrapeptide carboxypeptidase inhibitor	0.789	0.009	Myoclonus
	0.516	0.027	Aspartyltransferase inhibitor	0.747	0.004	Photoallergy dermatitis
	0.503	0.018	DNA synthesis inhibitor	0.661	0.034	Stomatitis
	0.436	0.002	Antibacterial. ophthalmic	0.864	0.004	Nail discoloration
	0.288	0.011	*Topoisomerase II inhibitor			
OF-3-EDA-GLA/ E4	0.736	0.031	Nootropic	0.898	0.010	Stomatitis
	0.555	0.002	Antibacterial. ophthalmic	0.890	0.004	Allergic contact dermatitis
	0.558	0.013	DNA synthesis inhibitor	0.872	0.003	Psychoses
	0.501	0.016	Antibacterial	0.868	0.004	Hyperglycemic
	0.501	0.031	Aspartyltransferase inhibitor	0.836	0.010	Asthma
	0.466	0.005	*Topoisomerase II inhibitor	0.898	0.010	Stomatitis
LF-3-EDA-GLA/ E5	0.736	0.031	Nootropic	0.890	0.004	Allergic contact dermatitis
	0.555	0.002	Antibacterial. ophthalmic	0.872	0.003	Psychoses
	0.558	0.013	DNA synthesis inhibitor	0.868	0.004	Hyperglycemic

Compound/ID	Mechanism of action			Toxic effects		
	Pa	Pi	Activity	Pa	Pi	Activity
	0.501	0.016	Antibacterial	0.836	0.010	Asthma
	0.501	0.031	Aspartyltransferase inhibitor	0.898	0.010	Stomatitis
	0.466	0.005	*Topoisomerase II inhibitor	0.864	0.004	Nail discoloration
MF-3-EDA-GLA/ E6	0.555	0.013	DNA synthesis inhibitor	0.888	0.011	Stomatitis
	0.541	0.013	Antibacterial	0.853	0.009	Asthma
	0.572	0.050	Nicotinic alpha4beta4 receptor agonist	0.812	0.009	Bradycardic
	0.563	0.047	Glutamate-5-semialdehyde dehydrogenase inhibitor	0.800	0.016	Hypertensive
	0.492	0.004	*Topoisomerase II inhibitor	0.992	0.002	Vascular toxicity
Etoposide	0.986	0.001	Cytostatic	0.992	0.001	Genotoxic
	0.956	0.004	Antineoplastic	0.955	0.002	Spermicide
	0.945	0.002	*Topoisomerase II inhibitor	0.957	0.004	Dyspnea
	0.925	0.002	DNA intercalator	0.951	0.004	Inflammation
	0.909	0.002	Anticarcinogenic	0.929	0.002	Carcinogenic, group 2A

*in the table are listed the first five positions for each compound regarding the most likely mechanisms of action by which they manifest their biological activity and the additional score for topoisomerase II inhibitory activity in case of the ones for which it was not included in the list of five. Also, the first five most likely toxic effects according to the platform are presented.

Table S8. Anti-cancer effect: most probable cell lines for which compounds exhibit cytotoxicity (Pa>0.4) (CLC-Pred platform)

Compound/ID	Pa	Pi	Cell line	Cell-line full name	Tissue	Tumour type	IAP*
CF-3-GLA/A1	0.575	0.027	HL-60	Promyeloblast leukaemia	Haematopoietic and lymphoid tissue	Leukaemia	0.881
	0.491	0.004	SiHa	Cervical squamous cell carcinoma	Cervix	Carcinoma	0.859
	0.454	0.102	OCI-AML2	Adult acute myeloid leukaemia	Blood	Leukaemia	0.803
	0.424	0.065	NCI-H441	Papillary adenocarcinoma	Lung	Adenocarcinoma	0.813
	0.421	0.079	MONO-MAC-6	Adult acute monocytic leukaemia	Blood	Leukaemia	0.807
	0.413	0.004	CAPAN-1	Pancreas Adenocarcinoma	Pancreas	Adenocarcinoma	0.889
	0.407	0.056	UMUC3	Bladder Carcinoma	Urinary tract	Carcinoma	0.810
NF-3-GLA/A2	0.551	0.031	HL-60	Promyeloblast leukaemia	Haematopoietic and lymphoid tissue	Leukaemia	0.881
	0.443	0.124	GIST882	Gastrointestinal stromal tumor	Intestine	Carcinoma	0.824
	0.437	0.121	OCI-AML2	Adult acute myeloid leukaemia	Blood	Leukaemia	0.803
	0.430	0.005	SiHa	Cervical squamous cell carcinoma	Cervix	Carcinoma	0.859
	0.428	0.118	GIST48	Gastrointestinal stromal tumor	Intestine	Carcinoma	0.805
	0.415	0.048	UMUC3	Bladder Carcinoma	Urinary tract	Carcinoma	0.810
	0.413	0.004	CAPAN-1	Pancreas Adenocarcinoma	Pancreas	Adenocarcinoma	0.889
	0.404	0.042	OE33	Barrett adenocarcinoma	Esophagus	Adenocarcinoma	0.805
PF-3-GLA/A3	0.578	0.027	HL-60	Promyeloblast leukaemia	Haematopoietic and lymphoid tissue	Leukaemia	0.881
	0.515	0.045	GIST48	Gastrointestinal stromal tumor	Intestine	Carcinoma	0.805
	0.514	0.065	GIST882	Gastrointestinal stromal tumor	Intestine	Carcinoma	0.824
	0.453	0.103	OCI-AML2	Adult acute myeloid leukaemia	Blood	Leukaemia	0.803
	0.431	0.004	CAPAN-1	Pancreas Adenocarcinoma	Pancreas	Adenocarcinoma	0.889
	0.427	0.006	SiHa	Cervical squamous cell carcinoma	Cervix	Carcinoma	0.859
	0.408	0.055	UMUC3	Bladder Carcinoma	Urinary tract	Carcinoma	0.810
OF-3-GLA/A4	0.750	0.007	HL-60	Promyeloblast	Haematopoietic	Leukaemia	0.881

Compound/ID	Pa	Pi	Cell line	Cell-line full name	Tissue	Tumour type	IAP*
				leukaemia	and lymphoid tissue		
	0.496	0.061	OCI-AML2	Adult acute myeloid leukaemia	Blood	Leukaemia	0.803
	0.480	0.003	CAPAN-1	Pancreas Adenocarcinoma	Pancreas	Adenocarcinoma	0.889
	0.456	0.102	SK-LU-1	Adenocarcinoma	Lung	Carcinoma	0.810
	0.434	0.057	NCI-H441	Papillary adenocarcinoma	Lung	Adenocarcinoma	0.813
	0.419	0.045	UMUC3	Bladder Carcinoma	Urinary tract	Carcinoma	0.810
	0.410	0.055	SNU-5	Gastric Carcinoma	Stomach	Carcinoma	0.828
	0.407	0.067	SK-MEL-1	Metastatic melanoma	Skin	Melanoma	0.808
	0.406	0.091	OPM-2	Plasma cell myeloma	Bone Marrow	Myeloma	0.823
	0.402	0.081	YAPC	Pancreatic carcinoma	Pancreas	Carcinoma	0.817
	0.401	0.100	HCC1937	Breast Carcinoma	Breast	Carcinoma	0.806
LF-3-GLA/A5	0.750	0.007	HL-60	Promyeloblast leukaemia	Haematopoietic and lymphoid tissue	Leukaemia	0.881
	0.496	0.061	OCI-AML2	Adult acute myeloid leukaemia	Blood	Leukaemia	0.803
	0.480	0.003	CAPAN-1	Pancreas Adenocarcinoma	Pancreas	Adenocarcinoma	0.889
	0.456	0.102	SK-LU-1	Adenocarcinoma	Lung	Carcinoma	0.810
	0.434	0.057	NCI-H441	Papillary adenocarcinoma	Lung	Adenocarcinoma	0.813
	0.419	0.045	UMUC3	Bladder Carcinoma	Urinary tract	Carcinoma	0.810
	0.410	0.055	SNU-5	Gastric Carcinoma	Stomach	Carcinoma	0.828
	0.407	0.067	SK-MEL-1	Metastatic melanoma	Skin	Melanoma	0.808
	0.406	0.091	OPM-2	Plasma cell myeloma	Bone Marrow	Myeloma	0.823
	0.402	0.081	YAPC	Pancreatic carcinoma	Pancreas	Carcinoma	0.817
	0.401	0.100	HCC1937	Breast Carcinoma	Breast	Carcinoma	0.806
MF-3-GLA/A6	0.440	0.058	OPM-2	Plasma cell myeloma	Bone Marrow	Myeloma	0.823
	0.433	0.124	OCI-AML2	Adult acute myeloid leukaemia	Blood	Leukaemia	0.803
	0.409	0.078	NCI-H441	Papillary adenocarcinoma	Lung	Adenocarcinoma	0.813
	0.404	0.034	HT1197	Carcinoma	Urinary bladder	Carcinoma	0.815
	0.403	0.079	YAPC	Pancreatic carcinoma	Pancreas	Carcinoma	0.817
	0.403	0.113	MONO-MAC-6	Adult acute monocytic	Blood	Leukaemia	0.807

Compound/ID	Pa	Pi	Cell line	Cell-line full name	Tissue	Tumour type	IAP*
				leukaemia			
	0.401	0.170	SK-LU-1	Adenocarcinoma	Lung	Carcinoma	0.810
CF-7-GLA/B1	0.605	0.023	HL-60	Promyeloblast leukaemia	Haematopoietic and lymphoid tissue	Leukaemia	0.881
	0.566	0.002	SiHa	Cervical squamous cell carcinoma	Cervix	Carcinoma	0.859
	0.480	0.075	OCI-AML2	Adult acute myeloid leukaemia	Blood	Leukaemia	0.803
	0.447	0.052	OPM-2	Plasma cell myeloma	Bone Marrow	Myeloma	0.823
	0.433	0.061	MONO-MAC-6	Adult acute monocytic leukaemia	Blood	Leukaemia	0.807
	0.402	0.004	CAPAN-1	Pancreas Adenocarcinoma	Pancreas	Adenocarcinoma	0.889
NF-7-GLA/B2	0.582	0.026	HL-60	Promyeloblast leukaemia	Haematopoietic and lymphoid tissue	Leukaemia	0.881
	0.530	0.003	SiHa	Cervical squamous cell carcinoma	Cervix	Carcinoma	0.859
	0.467	0.088	OCI-AML2	Adult acute myeloid leukaemia	Blood	Leukaemia	0.803
	0.416	0.081	OPM-2	Plasma cell myeloma	Bone Marrow	Myeloma	0.823
	0.413	0.095	MONO-MAC-6	Adult acute monocytic leukaemia	Blood	Leukaemia	0.807
	0.406	0.058	UMUC3	Bladder Carcinoma	Urinary tract	Carcinoma	0.810
	0.402	0.004	CAPAN-1	Pancreas Adenocarcinoma	Pancreas	Adenocarcinoma	0.889
MF-7-GLA/B6	0.481	0.004	SiHa	Cervical squamous cell carcinoma	Cervix	Carcinoma	0.859
	0.473	0.033	OPM-2	Plasma cell myeloma	Bone Marrow	Myeloma	0.823
	0.448	0.109	OCI-AML2	Adult acute myeloid leukaemia	Blood	Leukaemia	0.803
	0.415	0.091	MONO-MAC-6	Adult acute monocytic leukaemia	Blood	Leukaemia	0.807
CF-3-GLA-HA/C1	0.540	0.030	OCI-AML2	Adult acute myeloid leukaemia	Blood	Leukaemia	0.803
	0.462	0.004	SiHa	Cervical squamous cell carcinoma	Cervix	Carcinoma	0.859
	0.461	0.028	MONO-MAC-6	Adult acute monocytic leukaemia	Blood	Leukaemia	0.807
	0.454	0.019	Kasumi-1	Acute myeloblastic leukaemia	Blood	Leukaemia	0.816

Compound/ID	Pa	Pi	Cell line	Cell-line full name	Tissue	Tumour type	IAP*
	0.447	0.053	HL-60	Promyeloblast leukaemia	Haematopoietic and lymphoid tissue	Leukaemia	0.881
	0.440	0.009	HuP-T3	Pancreatic adenocarcinoma	Pancreas	Adenocarcinoma	0.868
	0.436	0.062	OPM-2	Plasma cell myeloma	Bone Marrow	Myeloma	0.823
	0.414	0.049	UMUC3	Bladder Carcinoma	Urinary tract	Carcinoma	0.810
	0.405	0.004	CAPAN-1	Pancreas Adenocarcinoma	Pancreas	Adenocarcinoma	0.889
NF-3-GLA-HA/C2	0.532	0.035	OCI-AML2	Adult acute myeloid leukaemia	Blood	Leukaemia	0.803
	0.457	0.017	Kasumi-1	Acute myeloblastic leukaemia	Blood	Leukaemia	0.816
	0.447	0.043	MONO-MAC-6	Adult acute monocytic leukaemia	Blood	Leukaemia	0.807
	0.428	0.011	HuP-T3	Pancreatic adenocarcinoma	Pancreas	Adenocarcinoma	0.868
	0.421	0.031	OE33	Barrett adenocarcinoma	Esophagus	Adenocarcinoma	0.805
	0.421	0.061	HL-60	Promyeloblast leukaemia	Haematopoietic and lymphoid tissue	Leukaemia	0.881
	0.420	0.043	UMUC3	Bladder Carcinoma	Urinary tract	Carcinoma	0.810
	0.405	0.004	CAPAN-1	Pancreas Adenocarcinoma	Pancreas	Adenocarcinoma	0.889
	0.404	0.094	OPM-2	Plasma cell myeloma	Bone Marrow	Myeloma	0.823
PF-3-GLA-HA/C3	0.537	0.032	OCI-AML2	Adult acute myeloid leukaemia	Blood	Leukaemia	0.803
	0.471	0.013	Kasumi-1	Acute myeloblastic leukaemia	Blood	Leukaemia	0.816
	0.464	0.081	GIST48	Gastrointestinal stromal tumor	Intestine	Carcinoma	0.805
	0.463	0.105	GIST882	Gastrointestinal stromal tumor	Intestine	Carcinoma	0.824
	0.458	0.051	HL-60	Promyeloblast leukaemia	Haematopoietic and lymphoid tissue	Leukaemia	0.881
	0.450	0.049	OPM-2	Plasma cell myeloma	Bone Marrow	Myeloma	0.823
	0.447	0.043	MONO-MAC-6	Adult acute monocytic leukaemia	Blood	Leukaemia	0.807
	0.422	0.004	CAPAN-1	Pancreas Adenocarcinoma	Pancreas	Adenocarcinoma	0.889
	0.420	0.014	HuP-T3	Pancreatic adenocarcinoma	Pancreas	Adenocarcinoma	0.868

Compound/ID	Pa	Pi	Cell line	Cell-line full name	Tissue	Tumour type	IAP*
	0.414	0.049	UMUC3	Bladder Carcinoma	Urinary tract	Carcinoma	0.810
	0.407	0.040	OE33	Barrett adenocarcinoma	Esophagus	Adenocarcinoma	0.805
	0.405	0.059	SNU-5	Gastric Carcinoma	Stomach	Carcinoma	0.828
	0.401	0.069	PC-3	Prostate carcinoma	Prostate	Carcinoma	0.883
OF-3-GLA-HA/C4	0.666	0.015	HL-60	Promyeloblast leukaemia	Haematopoietic and lymphoid tissue	Leukaemia	0.881
	0.555	0.023	OCI-AML2	Adult acute myeloid leukaemia	Blood	Leukaemia	0.803
	0.529	0.031	PC-3	Prostate carcinoma	Prostate	Carcinoma	0.883
	0.469	0.003	CAPAN-1	Pancreas Adenocarcinoma	Pancreas	Adenocarcinoma	0.889
	0.464	0.039	OPM-2	Plasma cell myeloma	Bone Marrow	Myeloma	0.823
	0.433	0.061	MONO-MAC-6	Adult acute monocytic leukaemia	Blood	Leukaemia	0.807
	0.423	0.041	UMUC3	Bladder Carcinoma	Urinary tract	Carcinoma	0.810
	0.415	0.047	Kasumi-1	Acute myeloblastic leukaemia	Blood	Leukaemia	0.816
	0.415	0.051	SNU-5	Gastric Carcinoma	Stomach	Carcinoma	0.828
	0.414	0.077	SW48	Colorectal Adenocarcinoma	Colon	Adenocarcinoma	0.806
	0.403	0.021	HuP-T3	Pancreatic adenocarcinoma	Pancreas	Adenocarcinoma	0.868
LF-3-GLA-HA/C5	0.666	0.015	HL-60	Promyeloblast leukaemia	Haematopoietic and lymphoid tissue	Leukaemia	0.881
	0.555	0.023	OCI-AML2	Adult acute myeloid leukaemia	Blood	Leukaemia	0.803
	0.529	0.031	PC-3	Prostate carcinoma	Prostate	Carcinoma	0.883
	0.469	0.003	CAPAN-1	Pancreas Adenocarcinoma	Pancreas	Adenocarcinoma	0.889
	0.464	0.039	OPM-2	Plasma cell myeloma	Bone Marrow	Myeloma	0.823
	0.433	0.061	MONO-MAC-6	Adult acute monocytic leukaemia	Blood	Leukaemia	0.807
	0.423	0.041	UMUC3	Bladder Carcinoma	Urinary tract	Carcinoma	0.810
	0.415	0.047	Kasumi-1	Acute myeloblastic leukaemia	Blood	Leukaemia	0.816
	0.415	0.051	SNU-5	Gastric Carcinoma	Stomach	Carcinoma	0.828
	0.414	0.077	SW48	Colorectal Adenocarcinoma	Colon	Adenocarcinoma	0.806
MF-3-GLA-HA/C6	0.521	0.042	OCI-AML2	Adult acute myeloid leukaemia	Blood	Leukaemia	0.803
	0.478	0.030	OPM-2	Plasma cell myeloma	Bone Marrow	Myeloma	0.823

Compound/ID	Pa	Pi	Cell line	Cell-line full name	Tissue	Tumour type	IAP*
	0.444	0.046	MONO-MAC-6	Adult acute monocytic leukaemia	Blood	Leukaemia	0.807
	0.425	0.012	HuP-T3	Pancreatic adenocarcinoma	Pancreas	Adenocarcinoma	0.868
	0.417	0.074	SW48	Colorectal Adenocarcinoma	Colon	Adenocarcinoma	0.806
CF-3-EA-GLA/D1	0.468	0.048	HL-60	Promyeloblast leukaemia	Haematopoietic and lymphoid tissue	Leukaemia	0.881
	0.454	0.005	SiHa	Cervical squamous cell carcinoma	Cervix	Carcinoma	0.859
	0.413	0.050	UMUC3	Bladder Carcinoma	Urinary tract	Carcinoma	0.810
NF-3-EA-GLA/D2	0.440	0.055	HL-60	Promyeloblast leukaemia	Haematopoietic and lymphoid tissue	Leukaemia	0.881
	0.420	0.044	UMUC3	Bladder Carcinoma	Urinary tract	Carcinoma	0.810
PF-3-EA-GLA/D3	0.477	0.046	HL-60	Promyeloblast leukaemia	Haematopoietic and lymphoid tissue	Leukaemia	0.881
	0.472	0.074	GIST48	Gastrointestinal stromal tumor	Intestine	Carcinoma	0.805
	0.464	0.104	GIST882	Gastrointestinal stromal tumor	Intestine	Carcinoma	0.824
	0.414	0.049	UMUC3	Bladder Carcinoma	Urinary tract	Carcinoma	0.810
OF-3-EA-GLA/D4	0.678	0.014	HL-60	Promyeloblast leukaemia	Haematopoietic and lymphoid tissue	Leukaemia	0.881
	0.456	0.099	OCI-AML2	Adult acute myeloid leukaemia	Blood	Leukaemia	0.803
	0.446	0.003	CAPAN-1	Pancreas Adenocarcinoma	Pancreas	Adenocarcinoma	0.889
	0.423	0.042	UMUC3	Bladder Carcinoma	Urinary tract	Carcinoma	0.810
	0.406	0.091	OPM-2	Plasma cell myeloma	Bone Marrow	Myeloma	0.823
LF-3-EA-GLA/D5	0.678	0.014	HL-60	Promyeloblast leukaemia	Haematopoietic and lymphoid tissue	Leukaemia	0.881
	0.456	0.099	OCI-AML2	Adult acute myeloid leukaemia	Blood	Leukaemia	0.803
	0.446	0.003	CAPAN-1	Pancreas Adenocarcinoma	Pancreas	Adenocarcinoma	0.889
	0.423	0.042	UMUC3	Bladder Carcinoma	Urinary tract	Carcinoma	0.810
	0.406	0.091	OPM-2	Plasma cell myeloma	Bone Marrow	Myeloma	0.823
MF-3-EA-GLA/D6	0.438	0.060	OPM-2	Plasma cell myeloma	Bone Marrow	Myeloma	0.823
	0.401	0.064	UMUC3	Bladder Carcinoma	Urinary tract	Carcinoma	0.810
CF-3-EDA-GLA/E1	0.489	0.044	HL-60	Promyeloblast	Haematopoietic	Leukaemia	0.881

Compound/ID	Pa	Pi	Cell line	Cell-line full name	Tissue	Tumour type	IAP*
				leukaemia	and lymphoid tissue		
	0.467	0.004	SiHa	Cervical squamous cell carcinoma	Cervix	Carcinoma	0.859
	0.447	0.109	OCI-AML2	Adult acute myeloid leukaemia	Blood	Leukaemia	0.803
	0.422	0.042	UMUC3	Bladder Carcinoma	Urinary tract	Carcinoma	0.810
	0.410	0.100	MONO-MAC-6	Adult acute monocytic leukaemia	Blood	Leukaemia	0.807
NF-3-EDA-GLA/E2	0.469	0.100	GIST882	Gastrointestinal stromal tumor	Intestine	Carcinoma	0.824
	0.460	0.050	HL-60	Promyeloblast leukaemia	Haematopoietic and lymphoid tissue	Leukaemia	0.881
	0.440	0.104	GIST48	Gastrointestinal stromal tumor	Intestine	Carcinoma	0.805
	0.431	0.127	OCI-AML2	Adult acute myeloid leukaemia	Blood	Leukaemia	0.803
	0.428	0.037	UMUC3	Bladder Carcinoma	Urinary tract	Carcinoma	0.810
	0.404	0.008	SiHa	Cervical squamous cell carcinoma	Cervix	Carcinoma	0.859
PF-3-EDA-GLA/E3	0.527	0.057	GIST882	Gastrointestinal stromal tumor	Intestine	Carcinoma	0.824
	0.516	0.044	GIST48	Gastrointestinal stromal tumor	Intestine	Carcinoma	0.805
	0.496	0.042	HL-60	Promyeloblast leukaemia	Haematopoietic and lymphoid tissue	Leukaemia	0.881
	0.447	0.110	OCI-AML2	Adult acute myeloid leukaemia	Blood	Leukaemia	0.803
	0.422	0.042	UMUC3	Bladder Carcinoma	Urinary tract	Carcinoma	0.810
	0.410	0.004	CAPAN-1	Pancreas Adenocarcinoma	Pancreas	Adenocarcinoma	0.889
	0.402	0.009	SiHa	Cervical squamous cell carcinoma	Cervix	Carcinoma	0.859
OF-3-EDA-GLA/E4	0.689	0.013	HL-60	Promyeloblast leukaemia	Haematopoietic and lymphoid tissue	Leukaemia	0.881
	0.489	0.068	OCI-AML2	Adult acute myeloid leukaemia	Blood	Leukaemia	0.803
	0.459	0.003	CAPAN-1	Pancreas Adenocarcinoma	Pancreas	Adenocarcinoma	0.889
	0.430	0.036	UMUC3	Bladder Carcinoma	Urinary tract	Carcinoma	0.810
	0.422	0.076	OPM-2	Plasma cell myeloma	Bone Marrow	Myeloma	0.823
LF-3-EDA-GLA/E5	0.689	0.013	HL-60	Promyeloblast leukaemia	Haematopoietic and lymphoid tissue	Leukaemia	0.881

Compound/ID	Pa	Pi	Cell line	Cell-line full name	Tissue	Tumour type	IAP*
	0.489	0.068	OCI-AML2	Adult acute myeloid leukaemia	Blood	Leukaemia	0.803
	0.459	0.003	CAPAN-1	Pancreas Adenocarcinoma	Pancreas	Adenocarcinoma	0.889
	0.430	0.036	UMUC3	Bladder Carcinoma	Urinary tract	Carcinoma	0.810
	0.422	0.076	OPM-2	Plasma cell myeloma	Bone Marrow	Myeloma	0.823
MF-3-EDA-GLA/E6	0.447	0.052	OPM-2	Plasma cell myeloma	Bone Marrow	Myeloma	0.823
	0.428	0.130	OCI-AML2	Adult acute myeloid leukaemia	Blood	Leukaemia	0.803
	0.408	0.056	UMUC3	Bladder Carcinoma	Urinary tract	Carcinoma	0.810
Etoposide**	0.949	0.000	GLC4	Lung small cell carcinoma	Lung	Carcinoma	1.000
	0.948	0.005	A549	Lung carcinoma	Lung	Carcinoma	0.856
	0.937	0.002	SK-LU-1	Adenocarcinoma	Lung	Carcinoma	0.810
	0.898	0.001	ZR-75-1	Breast carcinoma	Breast	Carcinoma	0.965
	0.874	0.001	NCI-H647	Adenosquamous lung carcinoma	Lung	Carcinoma	0.889
	0.861	0.005	HeLa	Cervical adenocarcinoma	Cervix	Adenocarcinoma	0.885
	0.837	0.001	K562/Adr	Blast phase chronic myelogenous leukaemia, BCR-ABL1 positive	Haematopoietic and lymphoid tissue	Leukaemia	0.972
	0.820	0.003	SW48	Colorectal Adenocarcinoma	Colon	Adenocarcinoma	0.806
	0.817	0.012	MCF7	Breast carcinoma	Breast	Carcinoma	0.836
	0.798	0.003	NCI-H520	Squamous Cell Carcinoma	Lung	Carcinoma	0.837

*Invariant Accuracy of Prediction (equal to AUC value)

**The table presents only the first ten results for etoposide as there were predicted 99 cell lines with Pa > 0.4

Table S9. Metabolic pathways (Toxtree)

Compound/ID	Primary sites of metabolism	Secondary sites of metabolism	Tertiary sites of metabolism	Quaternary sites of metabolism
CF-3-GLA/A1	N-dealkylation	N-dealkylation	Amine hydroxylation	Aromatic hydroxylation
NF-3-GLA/A2	N-dealkylation	N-dealkylation	Amine hydroxylation	Aromatic hydroxylation
PF-3-GLA/A3	N-dealkylation	N-dealkylation	N-dealkylation	N-oxidation
OF-3-GLA/A4	N-dealkylation	N-dealkylation	N-dealkylation	N-oxidation
LF-3-GLA/A5	N-dealkylation	N-dealkylation	N-dealkylation	N-oxidation
MF-3-GLA/A6	N-dealkylation	N-dealkylation	N-dealkylation	No metabolites predicted
CF-7-GLA/ B1	N-dealkylation	N-dealkylation	Amine hydroxylation	Aromatic hydroxylation

Compound/ID	Primary sites of metabolism	Secondary sites of metabolism	Tertiary sites of metabolism	Quaternary sites of metabolism
NF-7-GLA/B2	N-dealkylation	N-dealkylation	Amine hydroxylation	Aromatic hydroxylation
MF-7-GLA/B6	N-dealkylation	N-dealkylation	-	Amine hydroxylation
CF-3-GLA-HA/C1	N-dealkylation	N-dealkylation	Amine hydroxylation	Aromatic hydroxylation
NF-3-GLA-HA/C2	N-dealkylation	N-dealkylation	Amine hydroxylation	Aromatic hydroxylation
PF-3-GLA-HA/C3	N-dealkylation	N-dealkylation	N-dealkylation	N-oxidation
OF-3-GLA-HA/C4	N-dealkylation	N-dealkylation	N-dealkylation	N-oxidation
LF-3-GLA-HA/C5	N-dealkylation	N-dealkylation	N-dealkylation	N-oxidation
MF-3-GLA-HA/C6	N-dealkylation	N-dealkylation	N-dealkylation	-
CF-3-EA-GLA/D1	N-dealkylation	N-dealkylation	-	Amine hydroxylation
NF-3-EA-GLA/D2	N-dealkylation	N-dealkylation	-	Amine hydroxylation
PF-3-EA-GLA/D3	N-dealkylation	N-dealkylation	-	N-dealkylation
OF-3-EA-GLA/D4	N-dealkylation	N-dealkylation	-	N-dealkylation
LF-3-EA-GLA/D5	N-dealkylation	N-dealkylation	-	N-dealkylation
MF-3-EA-GLA/D6	N-dealkylation	N-dealkylation	-	N-dealkylation
CF-3-EDA-GLA/E1	N-dealkylation	N-dealkylation	-	Amine hydroxylation
NF-3-EDA-GLA/E2	N-dealkylation	N-dealkylation	-	Amine hydroxylation
PF-3-EDA-GLA/E3	N-dealkylation	N-dealkylation	-	N-dealkylation
OF-3-EDA-GLA/E4	N-dealkylation	N-dealkylation	-	N-dealkylation
LF-3-EDA-GLA/E5	N-dealkylation	N-dealkylation	-	N-dealkylation
MF-3-EDA-GLA/E6	N-dealkylation	N-dealkylation	-	N-dealkylation
Etoposide	Dioxolane demethylation	Dioxolane demethylation	Dioxolane demethylation	Aliphatic hydroxylation/ O-dealkylation

Table S10. Compound metabolism assessed using SmartCyp

Compound/ID	3A4		2D6		2C9	
	The most reactive atom	Score	The most reactive atom	Score	The most reactive atom	Score
CF-3-GLA/A1	C20	32.9	C4	78.8	C20	51.7
NF-3-GLA/A2	C28	32.9	C11	78.8	C28	51.7
PF-3-GLA/A3	C31	30.6	C11	78.8	C31	38.6
OF-3-GLA/A4	C11	30.6	C29	78.8	C11	38.6
LF-3-GLA/A5	C11	30.6	C29	78.8	C11	38.6
MF-3-GLA/A6	C10	31.7	C32	78.8	C10	39.7
CF-7-GLA/B1	C2	33.7	C28	83.4	C2	52.5
NF-7-GLA/B2	C28	33.7	C2	82.8	C28	52.5
MF-7-GLA/B6	C18	33.7	C6	67.0	C18	52.5
CF-3-GLA-HA/C1	C21	32.8	C14	90.1	C21	51.7
NF-3-GLA-HA/C2	C29	32.8	C2	89.5	C29	51.7
PF-3-GLA-HA/C3	C32	30.6	C32	85.5	C32	38.6
OF-3-GLA-HA/C4	C11	30.6	C11	85.5	C11	38.6
LF-3-GLA-HA/C5	C11	30.6	C11	85.5	C11	38.6
MF-3-GLA-HA/C6	C10	31.7	C10	86.6	C10	39.7
CF-3-EA-GLA/D1	C23	32.7	N25	53.0	C23	51.7
NF-3-EA-GLA/D2	C31	32.7	N33	53	C31	51.7
PF-3-EA-GLA/D3	C34	30.6	C34	38.6	C34	38.6
OF-3-EA-GLA/D4	C11	30.6	C11	38.6	C11	38.6
LF-3-EA-GLA/D5	C11	30.6	C11	38.6	C11	38.6
MF-3-EA-GLA/D6	C10	31.7	C10	39.7	C10	39.7
CF-3-EDA-GLA/E1	C23	32.7	N25	53.0	C23	51.7
NF-3-EDA-GLA/E2	C30	32.7	C16	78.7	C30	51.7
PF-3-EDA-GLA/E3	C34	30.6	C34	38.6	C34	38.6
OF-3-EDA-GLA/E4	C11	30.6	C11	38.6	C11	38.6
LF-3-EDA-GLA/E5	C11	30.6	C11	38.6	C11	38.6
MF-3-EDA-GLA/E6	C10	31.7	C10	39.7	C10	39.7
Etoposide	C28	40.3	C2	54.7	C2	53.9

The structures were introduced in the platform using the SMILES string

Table S11. Acute toxicity in rodents when administered intraperitoneally, intravenously, orally and subcutaneously: LD50 in mg/kg

Compound/ID	Rat IP LD50 (mg/kg)	Rat IV LD50 (mg/kg)	Rat Oral LD50 (mg/kg)	Rat SC LD50 (mg/kg)
CF-3-GLA/A1	755.600 out of AD	407.800 in AD	1624.000 in AD	1601.000 in AD
NF-3-GLA/A2	659.000 out of AD	540.700 in AD	1454.000 in AD	1754.000 in AD
PF-3-GLA/A3	591.700 in AD	459.200 in AD	1396.000 in AD	2194.000 out of AD
OF-3-GLA/A4	486.300 in AD	342.400 in AD	966.100 in AD	1684.000 out of AD
LF-3-GLA/A5	486.300 in AD	342.400 in AD	966.100 in AD	1684.000 out of AD
MF-3-GLA/A6	80.240 out of AD	104.000 in AD	354.300 out of AD	815.000 in AD

Compound/ID	Rat IP LD50 (mg/kg)	Rat IV LD50 (mg/kg)	Rat Oral LD50 (mg/kg)	Rat SC LD50 (mg/kg)
CF-7-GLA/B1	1069.000 out of AD	555.300 in AD	1750.000 in AD	2150.000 in AD
NF-7-GLA/B2	839.100 in AD	668.400 in AD	1816.000 in AD	2293.000 in AD
MF-7-GLA/B6	486.900 out of AD	173.200 in AD	403.300 out of AD	1333.000 in AD
CF-3-GLA-HA/C1	528.000 out of AD	208.800 in AD	1461.000 in AD	969.800 in AD
NF-3-GLA-HA/C2	646.300 out of AD	340.700 in AD	1455.000 in AD	1171.000 in AD
PF-3-GLA-HA/C3	551.600 in AD	263.500 in AD	842.400 in AD	1006.000 in AD
OF-3-GLA-HA/C4	310.700 in AD	118.100 in AD	495.200 in AD	918.600 in AD
LF-3-GLA-HA/C5	310.700 in AD	118.100 in AD	495.200 in AD	918.600 in AD
MF-3-GLA-HA/C6	175.100 out of AD	87.750 in AD	338.100 out of AD	126.600 out of AD
CF-3-EA-GLA/D1	456.200 in AD	394.100 in AD	1442.000 in AD	2074.000 in AD
NF-3-EA-GLA/D2	534.600 in AD	581.100 in AD	1985.000 in AD	3896.000 out of AD
PF-3-EA-GLA/D3	389.900 in AD	441.200 in AD	1020.000 in AD	4290.000 out of AD
OF-3-EA-GLA/D4	329.700 in AD	221.800 in AD	708.200 in AD	2059.000 in AD
LF-3-EA-GLA/D5	329.700 in AD	221.800 in AD	708.200 in AD	2059.000 in AD
MF-3-EA-GLA/D6	651.100 out of AD	113.700 in AD	1137.000 in AD	381.400 in AD
CF-3-EDA-GLA/E1	804.900 out of AD	307.300 in AD	1392.000 in AD	1120.000 in AD
NF-3-EDA-GLA/E2	579.800 out of AD	373.100 in AD	1301.000 in AD	1157.000 in AD
PF-3-EDA-GLA/E3	453.400 in AD	352.900 in AD	937.900 in AD	1126.000 in AD
OF-3-EDA-GLA/E4	365.100 in AD	216.000 in AD	758.700 in AD	1476.000 in AD
LF-3-EDA-GLA/E5	365.100 in AD	216.000 in AD	758.700 in AD	1476.000 in AD
MF-3-EDA-GLA/E6	446.900 out of AD	108.900 in AD	1140.000 in AD	201.700 in AD
Etoposide	42.980 in AD	130.900 in AD	813.700 in AD	303.200 in AD

*IP - Intraperitoneal route of administration; IV – Intravenous; SC – Subcutaneous; in AD - compound falls in applicability domain of models; out of AD - compound is out of applicability domain of models

Table S12. Acute toxicity in rodents. Classification of Chemicals by OECD Project

Compound/ID	Rat IP LD50 Classification	Rat IV LD50 Classification	Rat Oral LD50 Classification	Rat SC LD50 Classification
CF-3-GLA/A1	Class 5 out of AD	Class 5 in AD	Class 4 in AD	Class 5 in AD
NF-3-GLA/A2	Class 5 out of AD	Class 5 in AD	Class 4 in AD	Class 5 in AD
PF-3-GLA/A3	Class 5 in AD	Class 5 in AD	Class 4 in AD	Class 5 out of AD
OF-3-GLA/A4	Class 4 in AD	Class 5 in AD	Class 4 in AD	Class 5 out of AD
LF-3-GLA/A5	Class 4 in AD	Class 5 in AD	Class 4 in AD	Class 5 out of AD
MF-3-GLA/A6	Class 4 out of AD	Class 4 in AD	Class 4 out of AD	Class 4 in AD
CF-7-GLA/B1	Class 5 out of AD	Class 5 in AD	Class 4 in AD	Class 5 in AD
NF-7-GLA/B2	Class 5 in AD	Class 5 in AD	Class 4 in AD	Class 5 in AD
MF-7-GLA/B6	Class 4 out of AD	Class 4 in AD	Class 4 out of AD	Class 5 in AD
CF-3-GLA-HA/C1	Class 5 out of AD	Class 4 in AD	Class 4 in AD	Class 4 in AD
NF-3-GLA-HA/C2	Class 5 out of AD	Class 5 in AD	Class 4 in AD	Class 5 in AD
PF-3-GLA-HA/C3	Class 5 in AD	Class 4 in AD	Class 4 in AD	Class 5 in AD
OF-3-GLA-HA/C4	Class 4 in AD	Class 4 in AD	Class 4 in AD	Class 4 in AD

Compound/ID	Rat IP LD50 Classification	Rat IV LD50 Classification	Rat Oral LD50 Classification	Rat SC LD50 Classification
LF-3-GLA-HA/C5	Class 4 in AD	Class 4 in AD	Class 4 in AD	Class 4 in AD
MF-3-GLA-HA/C6	Class 4 out of AD	Class 4 in AD	Class 4 out of AD	Class 3 out of AD
CF-3-EA-GLA/D1	Class 4 in AD	Class 5 in AD	Class 4 in AD	Class 5 in AD
NF-3-EA-GLA/D2	Class 5 in AD	Class 5 in AD	Class 4 in AD	Non-Toxic out of AD
PF-3-EA-GLA/D3	Class 4 in AD	Class 5 in AD	Class 4 in AD	Non-Toxic out of AD
OF-3-EA-GLA/D4	Class 4 in AD	Class 4 in AD	Class 4 in AD	Class 5 in AD
LF-3-EA-GLA/D5	Class 4 in AD	Class 4 in AD	Class 4 in AD	Class 5 in AD
MF-3-EA-GLA/D6	Class 5 out of AD	Class 4 in AD	Class 4 in AD	Class 4 in AD
CF-3-EDA-GLA/E1	Class 5 out of AD	Class 5 in AD	Class 4 in AD	Class 5 in AD
NF-3-EDA-GLA/E2	Class 5 out of AD	Class 5 in AD	Class 4 in AD	Class 5 in AD
PF-3-EDA-GLA/E3	Class 4 in AD	Class 5 in AD	Class 4 in AD	Class 5 in AD
OF-3-EDA-GLA/E4	Class 4 in AD	Class 4 in AD	Class 4 in AD	Class 5 in AD
LF-3-EDA-GLA/E5	Class 4 in AD	Class 4 in AD	Class 4 in AD	Class 5 in AD
MF-3-EDA-GLA/E6	Class 4 out of AD	Class 4 in AD	Class 4 in AD	Class 4 in AD
Etoposide	Class 3 in AD	Class 4 in AD	Class 4 in AD	Class 4 in AD

Table S13. Toxicity according to Cramer rules, Kroes TTC and Varhaar scheme (Toxtree program)

Compound/ID	Cramer rules	Kroess TTC		Verhaar scheme
	Toxic hazard	Estimate	Explanation	
CF-3-GLA/A1	High Class III	Substance would not be expected to be a safety concern	Verify structural alerts for potential genotoxic carcinogenicity	Class 5 (Not possible to classify according to these rules)
NF-3-GLA/A2	High Class III	Substance would not be expected to be a safety concern	Verify structural alerts for potential genotoxic carcinogenicity	Class 5 (Not possible to classify according to these rules)
PF-3-GLA/A3	High Class III	Substance would not be expected to be a safety concern	Verify structural alerts for potential genotoxic carcinogenicity	Class 5 (Not possible to classify according to these rules)
OF-3-GLA/A4	High Class III	Substance would not be expected to be a safety concern	Verify structural alerts for potential genotoxic carcinogenicity	Class 5 (Not possible to classify according to these rules)
LF-3-GLA/ A5	High Class III	Substance would not be expected to be a safety concern	Verify structural alerts for potential genotoxic carcinogenicity	Class 5 (Not possible to classify according to these rules)
MF-3-GLA/A6	High Class III	Substance would not be expected to be a safety concern	Verify structural alerts for potential genotoxic carcinogenicity	Class 5 (Not possible to classify according to these rules)
CF-7-GLA/B1	High Class III	Substance would not be expected to be a safety concern	Verify structural alerts for potential genotoxic carcinogenicity	Class 3 (unspecific reactivity)
NF-7-GLA/B2	High Class III	Substance would not be expected to be a safety concern	Verify structural alerts for potential genotoxic carcinogenicity	Class 5 (Not possible to classify according to these rules)

Compound/ID	Cramer rules	Kroess TTC		Verhaar scheme
	Toxic hazard	Estimate	Explanation	
MF-7-GLA/B6	High Class III	Substance would not be expected to be a safety concern	Verify structural alerts for potential genotoxic carcinogenicity	Class 3 (unspecific reactivity)
CF-3-GLA-HA/ C1	High Class III	Substance would not be expected to be a safety concern	Verify structural alerts for potential genotoxic carcinogenicity	Class 5 (Not possible to classify according to these rules)
NF-3-GLA-HA/ C2	High Class III	Substance would not be expected to be a safety concern	Verify structural alerts for potential genotoxic carcinogenicity	Class 5 (Not possible to classify according to these rules)
PF-3-GLA-HA/ C3	High Class III	Substance would not be expected to be a safety concern	Verify structural alerts for potential genotoxic carcinogenicity	Class 5 (Not possible to classify according to these rules)
OF-3-GLA-HA/ C4	High Class III	Substance would not be expected to be a safety concern	Verify structural alerts for potential genotoxic carcinogenicity	Class 5 (Not possible to classify according to these rules)
LF-3-GLA-HA/ C5	High Class III	Substance would not be expected to be a safety concern	Verify structural alerts for potential genotoxic carcinogenicity	Class 5 (Not possible to classify according to these rules)
MF-3-GLA-HA/ C6	High Class III	Substance would not be expected to be a safety concern	Verify structural alerts for potential genotoxic carcinogenicity	Class 5 (Not possible to classify according to these rules)
CF-3-EA-GLA/ D1	High Class III	Substance would not be expected to be a safety concern	Verify structural alerts for potential genotoxic carcinogenicity	Class 5 (Not possible to classify according to these rules)
NF-3-EA-GLA/ D2	High Class III	Substance would not be expected to be a safety concern	Verify structural alerts for potential genotoxic carcinogenicity	Class 5 (Not possible to classify according to these rules)
PF-3-EA-GLA/ D3	High Class III	Substance would not be expected to be a safety concern	Verify structural alerts for potential genotoxic carcinogenicity	Class 5 (Not possible to classify according to these rules)
OF-3-EA-GLA/ D4	High Class III	Substance would not be expected to be a safety concern	Verify structural alerts for potential genotoxic carcinogenicity	Class 5 (Not possible to classify according to these rules)
LF-3-EA-GLA/ D5	High Class III	Substance would not be expected to be a safety concern	Verify structural alerts for potential genotoxic carcinogenicity	Class 5 (Not possible to classify according to these rules)
MF-3-EA-GLA/ D6	High Class III	Substance would not be expected to be a safety concern	Verify structural alerts for potential genotoxic carcinogenicity	Class 5 (Not possible to classify according to these rules)
CF-3-EDA-GLA/ E1	High Class III	Substance would not be expected to be a safety concern	Verify structural alerts for potential genotoxic carcinogenicity	Class 5 (Not possible to classify according to these rules)
NF-3-EDA-GLA/ E2	High Class III	Substance would not be expected to be a safety concern	Verify structural alerts for potential genotoxic carcinogenicity	Class 5 (Not possible to classify according to these rules)
PF-3-EDA-GLA/ E3	High Class III	Substance would not be expected to be a safety concern	Verify structural alerts for potential genotoxic carcinogenicity	Class 5 (Not possible to classify according to these rules)
OF-3-EDA-	High Class III	Substance would	Verify structural alerts	Class 5 (Not possible to

Compound/ID	Cramer rules	Kroess TTC		Verhaar scheme
	Toxic hazard	Estimate	Explanation	
GLA/ E4		not be expected to be a safety concern	for potential genotoxic carcinogenicity	classify according to these rules)
LF-3-EDA-GLA/ E5	High Class III	Substance would not be expected to be a safety concern	Verify structural alerts for potential genotoxic carcinogenicity	Class 5 (Not possible to classify according to these rules)
MF-3-EDA-GLA/ E6	High Class III	Substance would not be expected to be a safety concern	Verify structural alerts for potential genotoxic carcinogenicity	Class 5 (Not possible to classify according to these rules)
Etoposide	High Class III	Substance would not be expected to be a safety concern	Verify structural alerts for potential genotoxic carcinogenicity	Class 1 (narcosis or baseline toxicity)

Table S14. Carcinogenic (genotoxic and non-genotoxic) and mutagenic effects evaluated using Toxtree and Osiris Property Explorer

Compound/ID	Carcinogenicity			Mutagenicity	
	Genotoxic Toxtree	Non-genotoxic	Tumorigenesis OSIRIS	Toxtree (Ames test) *S. typhimurium*	OSIRIS
CF-3-GLA/A1	Negative	Negative	Negative	Negative	Negative
NF-3-GLA/A2	Negative	Negative	Negative	Negative	Negative
PF-3-GLA/A3	Negative	Negative	Negative	Negative	Negative
OF-3-GLA/A4	Negative	Negative	Negative	Negative	Negative
LF-3-GLA/A5	Negative	Negative	Negative	Negative	Negative
MF-3-GLA/A6	Negative	Negative	Negative	Negative	Negative
CF-7-GLA/B1	Negative	Negative	Negative	Negative	Negative
NF-7-GLA/B2	Negative	Negative	Negative	Negative	Negative
MF-7-GLA/B6	Negative	Negative	Negative	Negative	Negative
CF-3-GLA-HA/C1	Negative	Negative	Negative	Positive	Negative
NF-3-GLA-HA/C2	Negative	Negative	Negative	Positive	Negative
PF-3-GLA-HA/C3	Negative	Negative	Negative	Positive	Negative
OF-3-GLA-HA/C4	Negative	Negative	Negative	Positive	Negative
LF-3-GLA-HA/C5	Negative	Negative	Negative	Positive	Negative
MF-3-GLA-HA/C6	Negative	Negative	Negative	Positive	Negative
CF-3-EA-GLA/D1	Negative	Negative	Negative	Negative	Negative
NF-3-EA-GLA/D2	Negative	Negative	Negative	Negative	Negative
PF-3-EA-GLA/D3	Negative	Negative	Negative	Negative	Negative
OF-3-EA-GLA/D4	Negative	Negative	Negative	Negative	Negative
LF-3-EA-GLA/D5	Negative	Negative	Negative	Negative	Negative
MF-3-EA-GLA/D6	Negative	Negative	Negative	Negative	Negative
CF-3-EDA-GLA/E1	Negative	Negative	Negative	Negative	Negative
NF-3-EDA-GLA/E2	Negative	Negative	Negative	Negative	Negative
PF-3-EDA-GLA/E3	Negative	Negative	Negative	Negative	Negative
OF-3-EDA-GLA/E4	Negative	Negative	Negative	Negative	Negative

Compound/ID	Carcinogenicity			Mutagenicity	
	Genotoxic Toxtree	Non-genotoxic	Tumorigenesis OSIRIS	Toxtree (Ames test) *S. typhimurium*	OSIRIS
LF-3-EDA-GLA/E5	Negative	Negative	Negative	Negative	Negative
MF-3-EDA-GLA/E6	Negative	Negative	Negative	Negative	Negative
Etoposide	Negative	Structural alert for nongenotoxic carcinogenicity	Negative	Negative	Negative

Table S15. Irritant/corrosive effect on the skin and eyes, effect on the reproductive system, biodegradability, protein and DNA binding alerts, assessed using Toxtree and Osiris Property Explorer

Compound/ID	Irritation/corrosion			Effect on the reproductive system	Biodegradability	Protein binding Alerts	DNA binding Alerts
	Skin	Eye	OSIRIS	OSIRIS			
CF-3-GLA/A1	Not corrosive	No skin corrosion R34 or R35	Negative	Negative	Class 2 (persistent chemical)	Alert for Michael Acceptor identified.	Alert for Michael Acceptor identified.
NF-3-GLA/A2	Not corrosive	No skin corrosion R34 or R35	Negative	Negative	Class 2 (persistent chemical)	Alert for Michael Acceptor identified.	Alert for Michael Acceptor identified.
PF-3-GLA/A3	Not corrosive	No skin corrosion R34 or R35	Negative	Medium risk fragments	Class 2 (persistent chemical)	Alert for Michael Acceptor identified.	Alert for SN1 and Michael Acceptor identified.
OF-3-GLA/A4	Not corrosive	No skin corrosion R34 or R35	Negative	Negative	Class 2 (persistent chemical)	Alert for Michael Acceptor identified.	Alert for SN1 and Michael Acceptor identified.
LF-3-GLA/A5	Not corrosive	No skin corrosion R34 or R35	Negative	Negative	Class 2 (persistent chemical)	Alert for Michael Acceptor identified.	Alert for SN1 and Michael Acceptor identified.
MF-3-GLA/A6	Not corrosive	No skin corrosion R34 or R35	Negative	Negative	Class 2 (persistent chemical)	Alert for Michael Acceptor identified.	Alert for Michael Acceptor identified.
CF-7-GLA/B1	Not corrosive	No skin corrosion R34 or R35	Negative	Negative	Class2 (persistent chemical)	Alert for Michael Acceptor identified.	Alert for SN1 and Michael Acceptor identified.

Compound/ID	Irritation/corrosion			Effect on the reproductive system	Biodegradability	Protein binding Alerts	DNA binding Alerts
	Skin	Eye	OSIRIS	OSIRIS			
NF-7-GLA/B2	Not corrosive	No skin corrosion R34 or R35	Negative	Negative	Class 2 (persistent chemical)	Alert for Michael Acceptor identified.	Alert for SN1 and Michael Acceptor identified.
MF-7-GLA/B6	Not corrosive	No skin corrosion R34 or R35	Negative	Negative	Class2 (persistent chemical)	Alert for Michael Acceptor identified.	Alert for SN1 and Michael Acceptor identified.
CF-3-GLA-HA/C1	Not corrosive	No skin corrosion R34 or R35	Negative	Negative	Class 2 (persistent chemical)	Alert for Michael Acceptor identified.	Alert for Michael Acceptor identified
NF-3-GLA-HA/C2	Not corrosive	No skin corrosion R34 or R35	Negative	Negative	Class2 (persistent chemical)	Alert for Michael Acceptor identified.	Alert for Michael Acceptor identified
PF-3-GLA-HA/C3	Not corrosive	No skin corrosion R34 or R35	Negative	Medium risk fragments	Class2 (persistent chemical)	Alert for Michael Acceptor identified	Alert for SN1 and Michael Acceptor identified.
OF-3-GLA-HA/C4	Not corrosive	No skin corrosion R34 or R35	Negative	Negative	Class2 (persistent chemical)	Alert for Michael Acceptor identified.	Alert for SN1 and Michael Acceptor identified.
LF-3-GLA-HA/C5	Not corrosive	No skin corrosion R34 or R35	Negative	Negative	Class 2 (persistent chemical)	Alert for Michael Acceptor identified.	Alert for SN1 and Michael Acceptor identified.
MF-3-GLA-HA/ C6	Not corrosive	No skin corrosion R34 or R35	Negative	Negative	Class 2 (persistent chemical)	Alert for Michael Acceptor identified.	Alert for Michael Acceptor identified
CF-3-EA-GLA/D1	Not corrosive	No skin corrosion R34 or R35	Negative	Negative	Class 2 (persistent chemical)	Alert for Michael Acceptor identified.	Alert for Michael Acceptor identified
NF-3-EA-GLA/D2	Not corrosive	No skin corrosion R34 or R35	Negative	Negative	Class 2 (persistent chemical)	Alert for Michael Acceptor identified.	Alert for Michael Acceptor identified
PF-3-EA-GLA/D3	Not corrosive	No skin corrosion R34	Negative	Medium risk fragments	Class 2 (persistent chemical)	Alert for Michael	Alert for SN1 and

Compound/ID	Irritation/corrosion			Effect on the reproductive system	Biodegradability	Protein binding Alerts	DNA binding Alerts
	Skin	Eye	OSIRIS	OSIRIS			
		or R35				Acceptor identified.	Michael Acceptor identified.
OF-3-EA-GLA/D4	Not corrosive	No skin corrosion R34 or R35	Negative	Negative	Class 2 (persistent chemical)	Alert for Michael Acceptor identified	Alert for SN1 and Michael Acceptor identified.
LF-3-EA-GLA/D5	Not corrosive	No skin corrosion R34 or R35	Negative	Negative	Class 2 (persistent chemical)	Alert for Michael Acceptor identified	Alert for SN1 and Michael Acceptor identified.
MF-3-EA-GLA/D6	Not corrosive	No skin corrosion R34 or R35	Negative	Negative	Class 2 (persistent chemical)	Alert for Michael Acceptor identified.	Alert for Michael Acceptor identified
CF-3-EDA-GLA/E1	Not corrosive	No skin corrosion R34 or R35	Negative	Negative	Class 2 (persistent chemical)	Alert for Michael Acceptor identified.	Alert for Michael Acceptor identified
NF-3-EDA-GLA/E2	Not corrosive	No skin corrosion R34 or R35	Negative	Negative	Class 2 (persistent chemical)	Alert for Michael Acceptor identified.	Alert for Michael Acceptor identified
PF-3-EDA-GLA/E3	Not corrosive	No skin corrosion R34 or R35	Negative	Medium risk fragments	Class 2 (persistent chemical)	Alert for Michael Acceptor identified.	Alert for SN1 and Michael Acceptor identified.
OF-3-EDA-GLA/E4	Not corrosive	No skin corrosion R34 or R35	Negative	Negative	Class 2 (persistent chemical)	Alert for Michael Acceptor identified.	Alert for SN1 and Michael Acceptor identified.
LF-3-EDA-GLA/E5	Not corrosive	No skin corrosion R34 or R35	Negative	Negative	Class 2 (persistent chemical)	Alert for Michael Acceptor identified.	Alert for SN1 and Michael Acceptor identified.
MF-3-EDA-GLA/E6	Not corrosive	No skin corrosion R34 or R35	Negative	Negative	Class 2 (persistent chemical)	Alert for Michael Acceptor identified.	Alert for Michael Acceptor identified
Etoposide	Not corrosive	No lesions R34, R35, R36, or R41	Negative	Negative	Class 2 (persistent chemical)	Alert for Michael Acceptor	Alert for Michael Acceptor

Compound/ID	Irritation/corrosion			Effect on the reproductive system	Biodegradability	Protein binding Alerts	DNA binding Alerts
	Skin	Eye	OSIRIS	OSIRIS			
						and SN2 identified	identified

*R34 – causes burns; R35- causes severe burns; R36 – significant eye irritation (middle category of irritancy); R41 – severe eye irritation (risk of serious damage to eyes) [41-45].

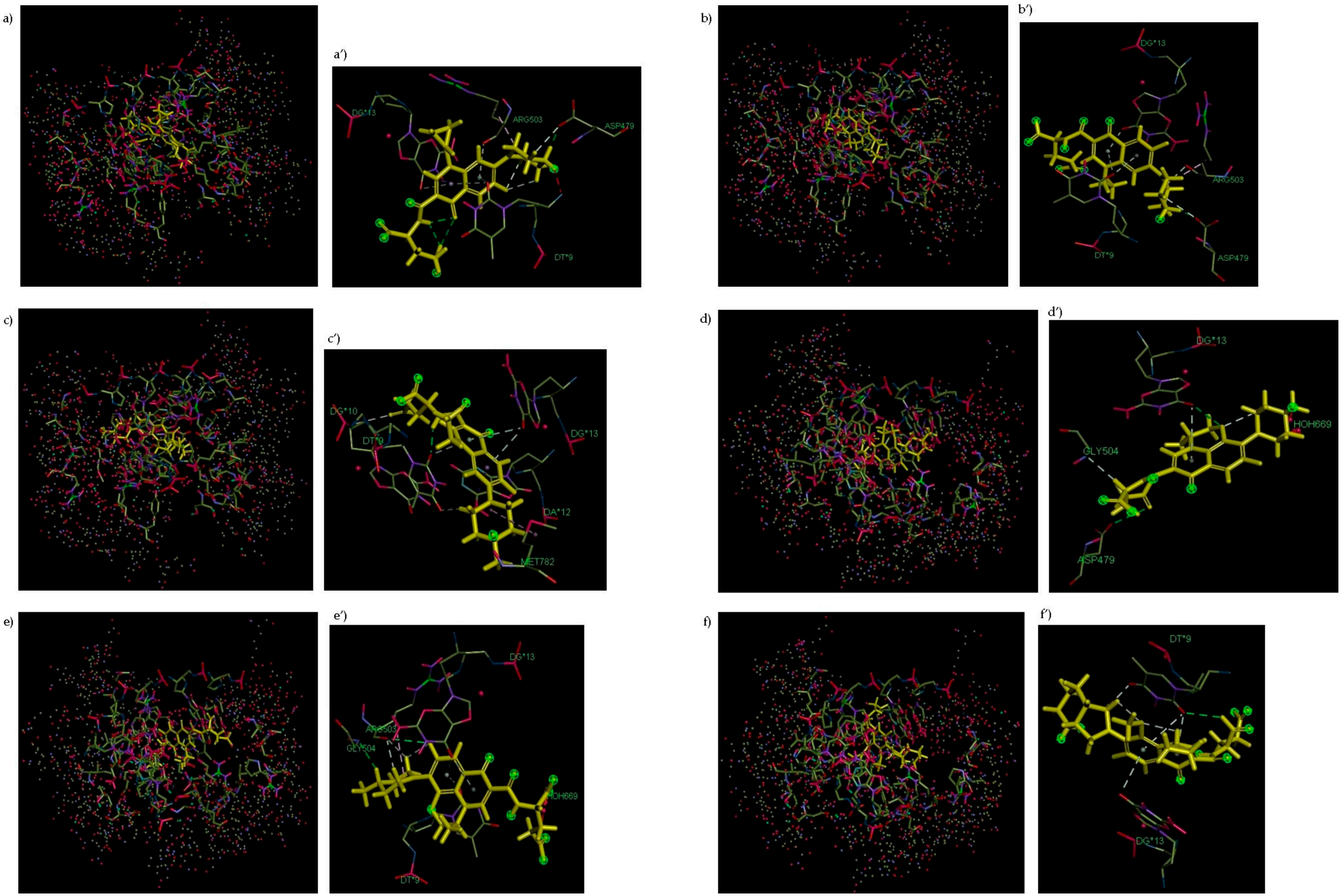

Figure S2. The conformations of hybrids A1-A6 (from class FQ-3-GLA) in the binding site (a-f) and their interaction with the enzyme-DNA complex (a′-f′)

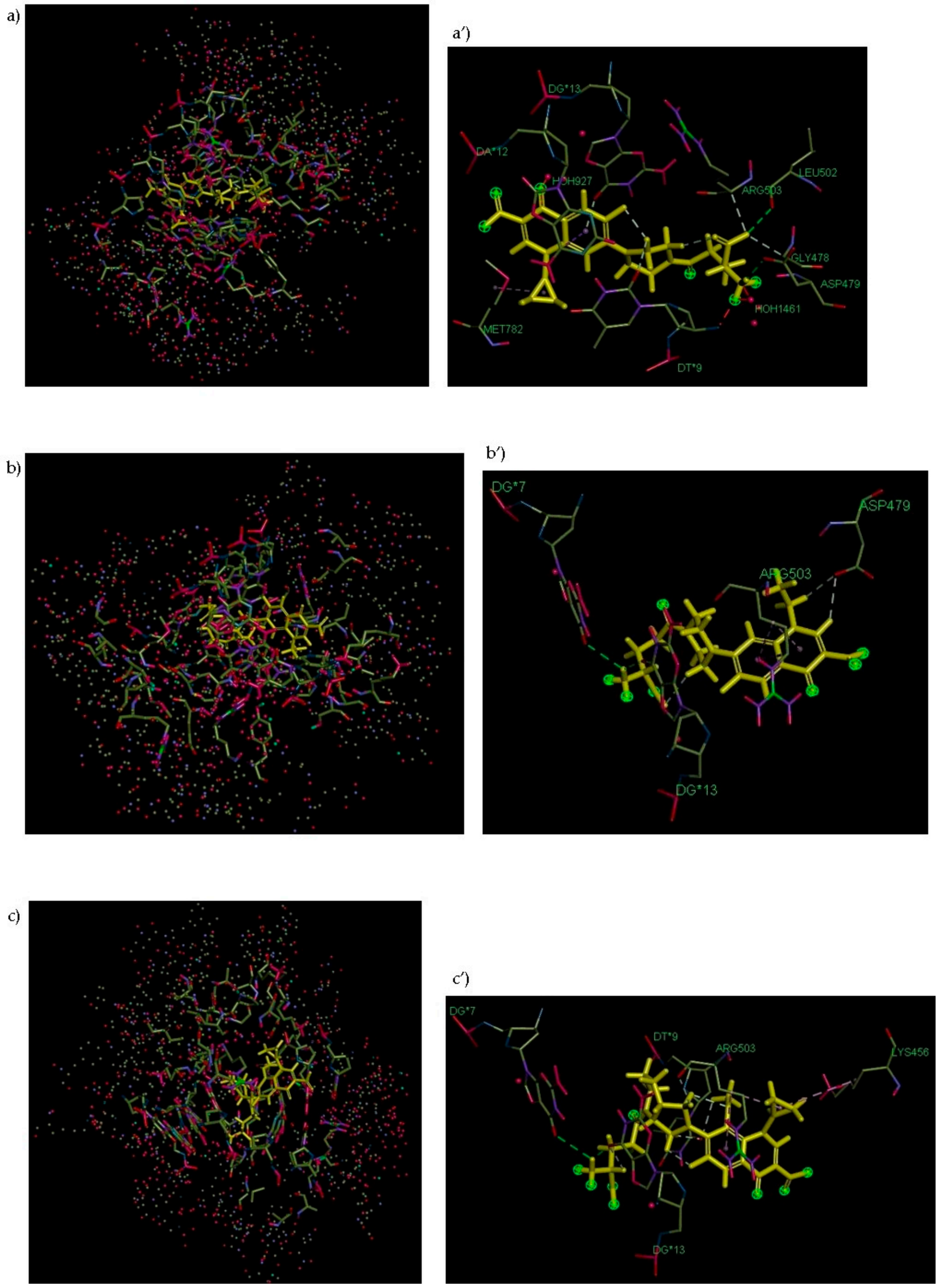

Figure S3. The conformations of hybrids B1, B2, and B6 (from class FQ-7-GLA) in the binding site (a-c) and their interaction with the enzyme-DNA complex (a′-c′)

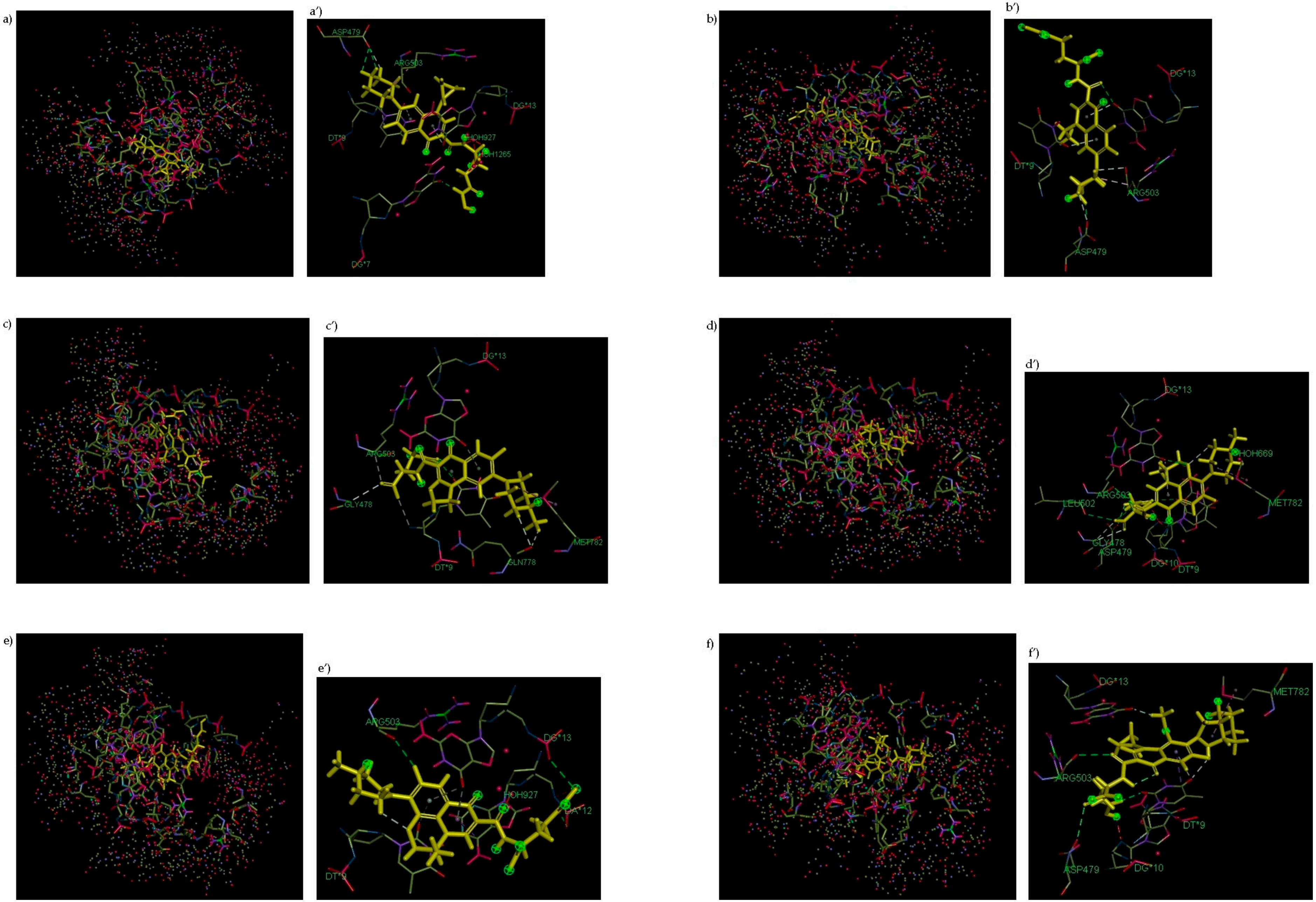

Figure S4. The conformations of hybrids C1-C6 (from class FQ-3-GLA-HA) in the binding site (a-f) and their interaction with the enzyme-DNA complex (a′-f′)

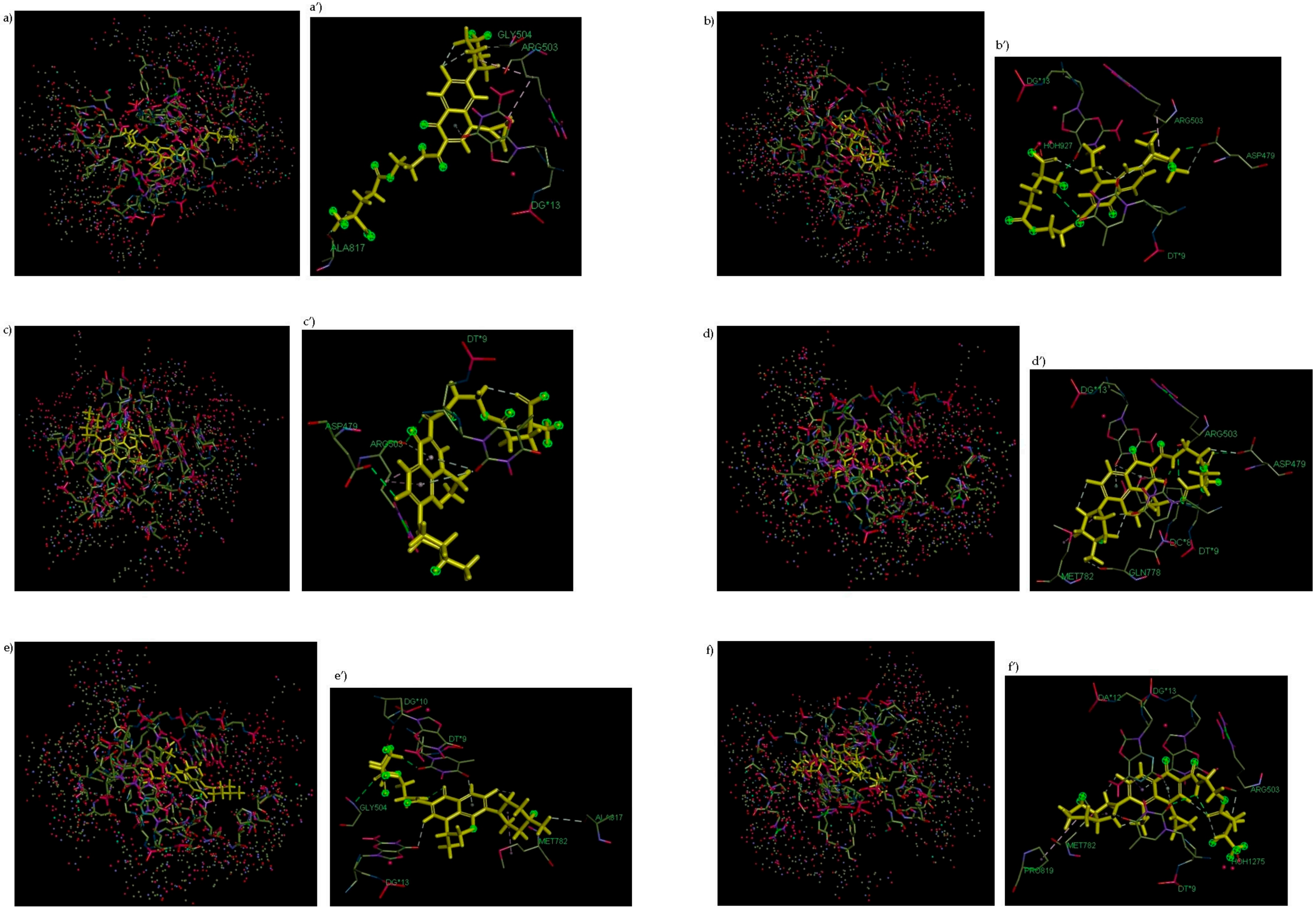

Figure S5. The conformations of hybrids D1-D6 (from class FQ-3-EA-GLA) in the binding site (a-f) and their interaction with the enzyme-DNA complex (a′-f′)

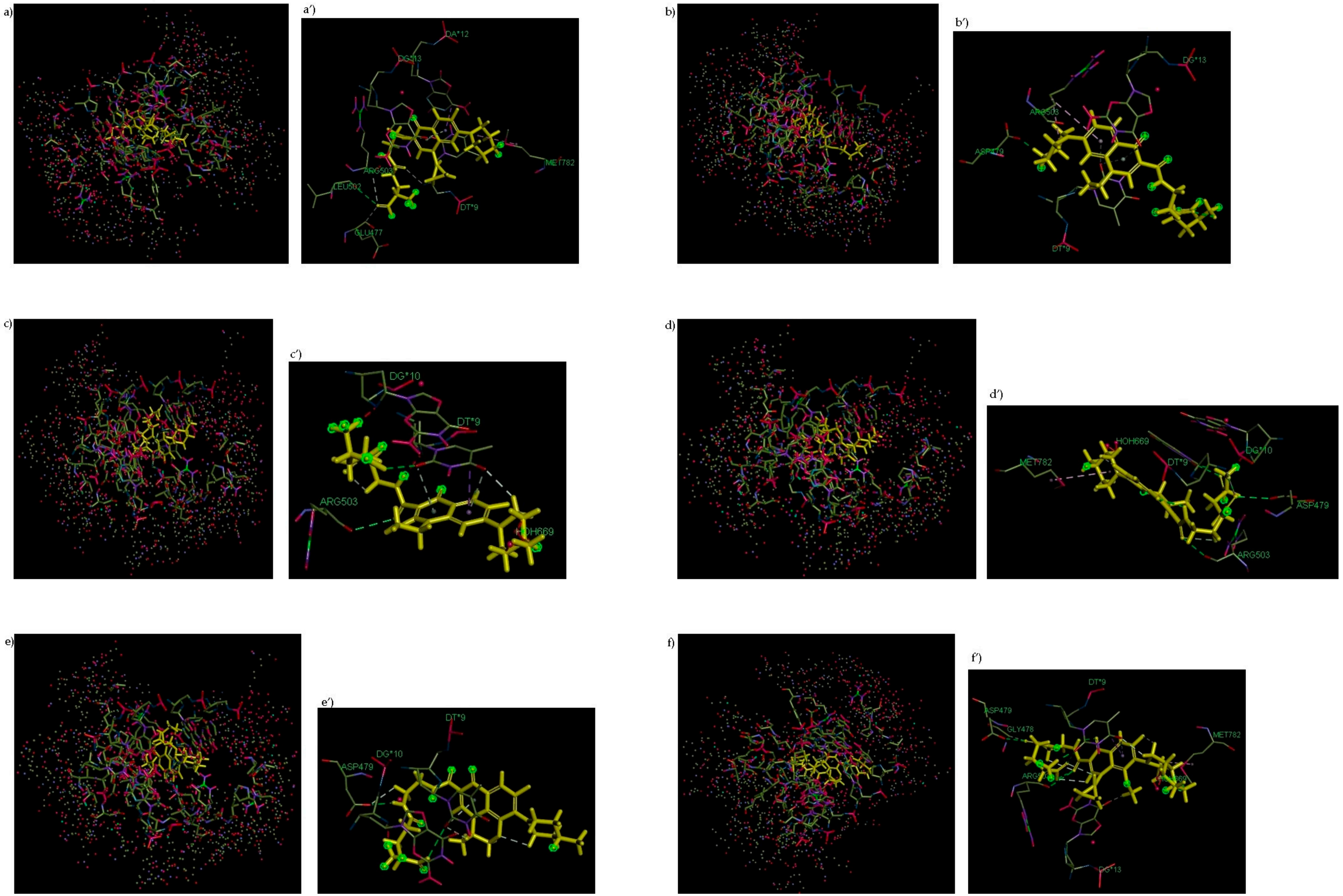

Figure S6. The conformations of hybrids E1-E6 (from class FQ-3-EDA-GLA) in the binding site (a-f) and their interaction with the enzyme-DNA complex (a′-f′)

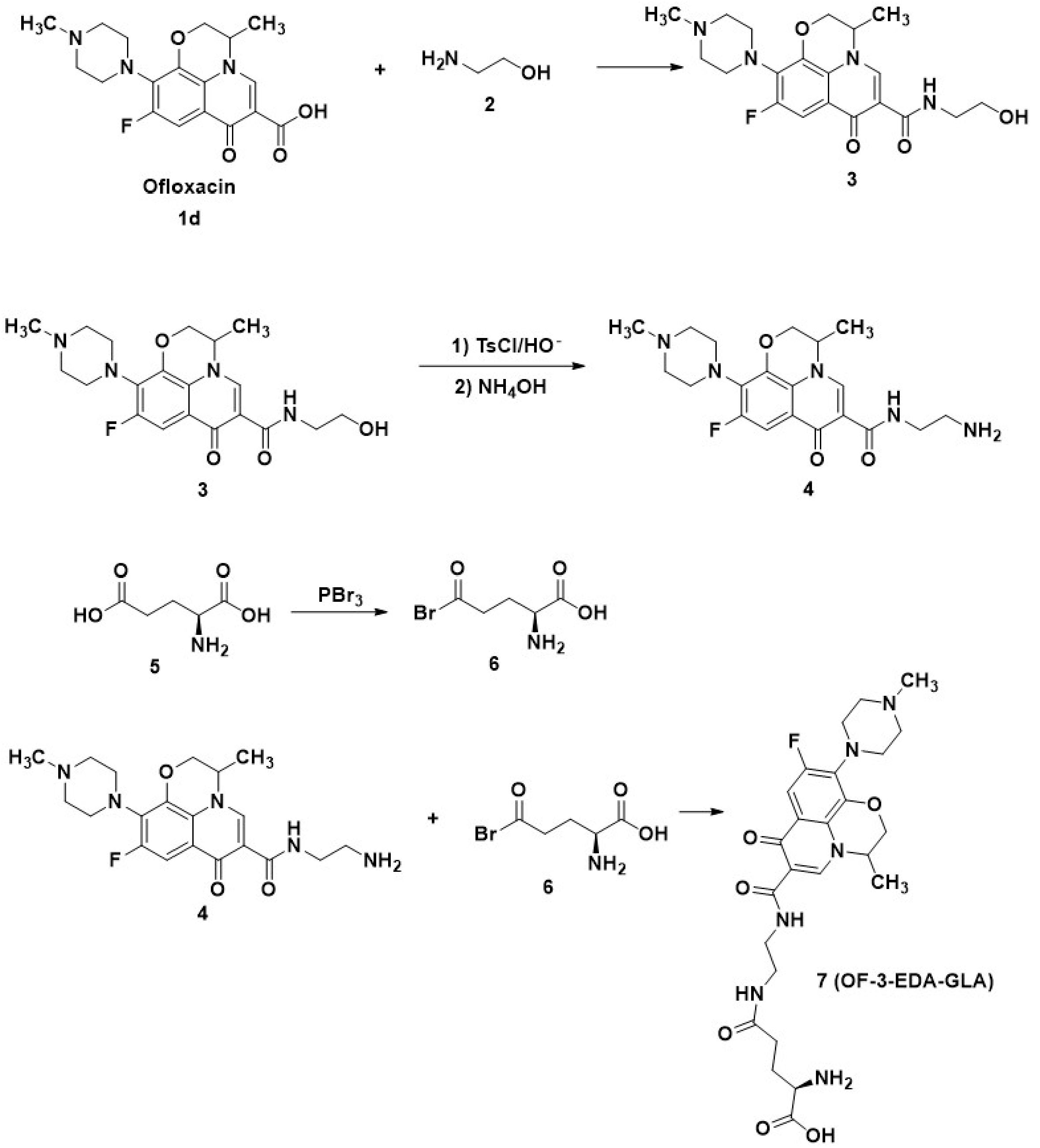

Figure S7. Synthesis proposal for hybrid OF-3-EDA-GLA.

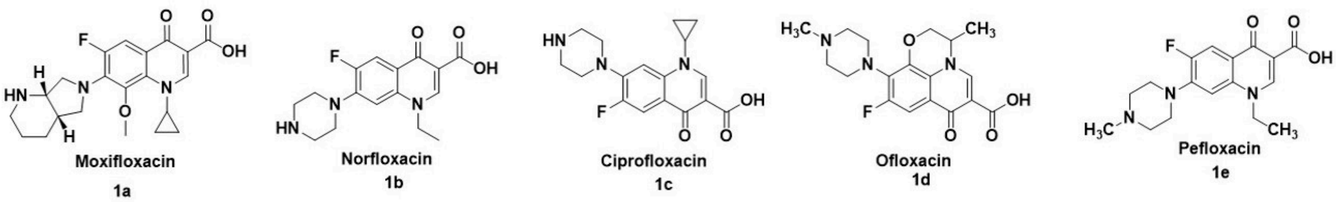

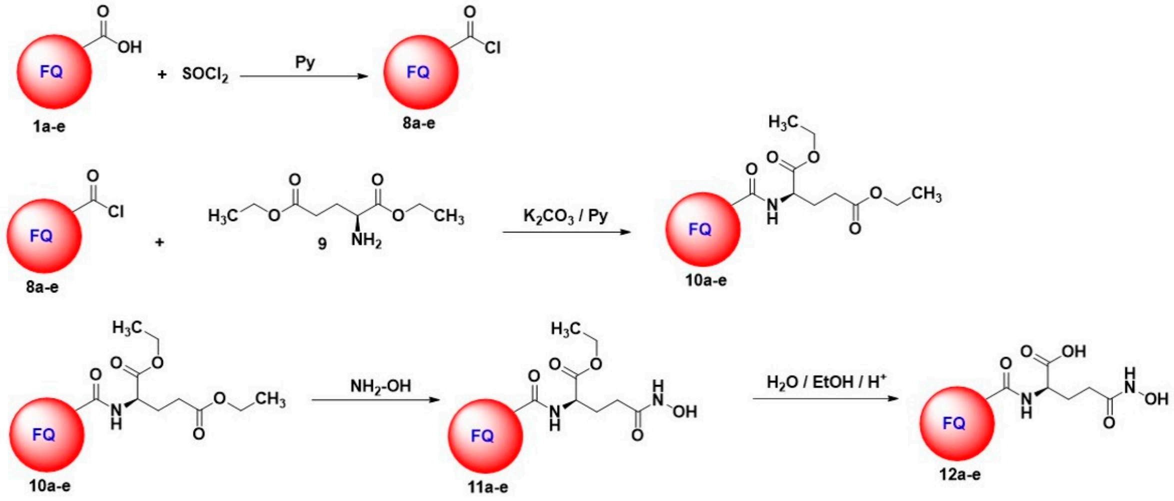

Figure S8. Synthesis proposal for hybrids FQ-3-GLA-HA.

Abbreviations

AD = Applicability Domain of models

ADME = Absorption, Distribution, Metabolism and Excretion

CLC-Pred = Cell Line Cytotoxicity Predictor

ESOL = Estimating Aqueous Solubility Directly from Molecular Structure

EtOH = Ethanol

FQ = Fluoroquinolone

IAC = Invariant Accuracy of Prediction

IP = Intraperitoneal route of administration

IV = Intravenous

LD50 = Lethal Dose 50

Pa = Probability to be Active

Pi = Probability to be Inactive

pI = Isoelectric point

Py = Pyridine

SC = Subcutaneous

SN1= Nucleophilic substitution type 1

SN2 = Aliphatic nucleophilic substitution type 2

TsCl = 4-Toluenesulfonyl chloride

TTC = Threshold of Toxicological Concern

References

1. Aranha, O.; Wood, D.P.; Sarkar, F.H. Ciprofloxacin Mediated Cell Growth Inhibition, S/G2-M Cell Cycle Arrest, and Apoptosis in a Human Transitional Cell Carcinoma of the Bladder Cell Line. *Clin. Cancer Res.* **2000**, *6*, 891–900.
2. Herold, C.; Ocker, M.; Ganslmayer, M.; Gerauer, H.; Hahn, E.G.; Schuppan, D. Ciprofloxacin Induces Apoptosis and Inhibits Proliferation of Human Colorectal Carcinoma Cells. *Br. J. Cancer* **2002**, *86*, 443–448. https://doi.org/10.1038/sj.bjc.6600079.
3. Kloskowski, T.; Olkowska, J.; Nazlica, A.; Drewa, T. The Influence of Ciprofloxacin on Hamster Ovarian Cancer Cell Line CHO AA8. *Acta Pol. Pharm.* **2010**, *67*, 345–349.
4. Fan, M.; Chen, S.; Weng, Y.; Li, X.; Jiang, Y.; Wang, X.; Bie, M.; An, L.; Zhang, M.; Chen, B.; et al. Ciprofloxacin Promotes Polarization of CD86+CD206- Macrophages to Suppress Liver Cancer. *Oncol. Rep.* **2020**, *44*, 91–102. https://doi.org/10.3892/or.2020.7602.
5. Beberok, A.; Wrześniok, D.; Minecka, A.; Rok, J.; Delijewski, M.; Rzepka, Z.; Respondek, M.; Buszman, E. Ciprofloxacin-Mediated Induction of S-Phase Cell Cycle Arrest and Apoptosis in COLO829 Melanoma Cells. *Pharmacol. Rep.* **2018**, *70*, 6–13. https://doi.org/10.1016/j.pharep.2017.07.007.
6. Kloskowski, T.; Gurtowska, N.; Olkowska, J.; Nowak, J.M.; Adamowicz, J.; Tworkiewicz, J.; Dębski, R.; Grzanka, A.; Drewa, T. Ciprofloxacin Is a Potential Topoisomerase II Inhibitor for the Treatment of NSCLC. *Int. J. Oncol.* **2012**, *41*, 1943–1949. https://doi.org/10.3892/ijo.2012.1653.
7. Beberok, A.; Wrześniok, D.; Rok, J.; Rzepka, Z.; Respondek, M.; Buszman, E.; Beberok, A.; Wrześniok, D.; Rok, J.; Rzepka, Z.; et al. Ciprofloxacin Triggers the Apoptosis of Human Triple-Negative Breast Cancer MDA-MB-231 Cells via the P53/Bax/Bcl-2 Signaling Pathway. *Int. J. Oncol.* **2018**, *52*, 1727–1737. https://doi.org/10.3892/ijo.2018.4310.
8. Zandi, A.; Zanjani, T.M.; Ziai, S.A.; Poul, Y.K.; Hoseini, M.H.M. Evaluation of the Cytotoxic Effects of Ciprofloxacin on Human Glioblastoma A-172 Cell Line. *Middle East. J. Cancer* **2017**, *8*, 119–126.
9. Yadav, V.; Varshney, P.; Sultana, S.; Yadav, J.; Saini, N. Moxifloxacin and Ciprofloxacin Induces S-Phase Arrest and Augments Apoptotic Effects of Cisplatin in Human Pancreatic Cancer Cells via ERK Activation. *BMC Cancer* **2015**, *15*, 581. https://doi.org/10.1186/s12885-015-1560-y.
10. Yadav, V.; Sultana, S.; Yadav, J.; Saini, N. Gatifloxacin Induces S and G2-Phase Cell Cycle Arrest in Pancreatic Cancer Cells via P21/P27/P53. *PLoS ONE* **2012**, *7*, e47796. https://doi.org/10.1371/journal.pone.0047796.
11. Kloskowski, T.; Frąckowiak, S.; Adamowicz, J.; Szeliski, K.; Rasmus, M.; Drewa, T.; Pokrywczyńska, M. Quinolones as a Potential Drug in Genitourinary Cancer Treatment—A Literature Review. *Front. Oncol.* **2022**, *12*, 890337. https://doi.org/10.3389/fonc.2022.890337.
12. Kloskowski, T.; Szeliski, K.; Fekner, Z.; Rasmus, M.; Dąbrowski, P.; Wolska, A.; Siedlecka, N.; Adamowicz, J.; Drewa, T.; Pokrywczyńska, M. Ciprofloxacin and Levofloxacin as Potential Drugs in Genitourinary Cancer Treatment—The Effect of Dose–Response on 2D and 3D Cell Cultures. *Int. J. Mol. Sci.* **2021**, *22*, 11970. https://doi.org/10.3390/ijms222111970.
13. Mamdooh, N.; Kasabri, V.; Al-Hiari, Y.; Almasri, I.; Al-Alawi, S.; Bustanji, Y. Evaluation of Selected Commercial Pharmacotherapeutic Drugs as Potential Pancreatic Lipase Inhibitors and Antiproliferative Compounds. *Drug Dev. Res.* **2019**, *80*, 310–324. https://doi.org/10.1002/ddr.21499.
14. Mohammed, H.H.H.; Abd El-Hafeez, A.A.; Abbas, S.H.; Abdelhafez, E.-S.M.N.; Abuo-Rahma, G.E.-D.A. New Antiproliferative 7-(4-(N-Substituted Carbamoylmethyl)Piperazin-1-Yl) Derivatives of Ciprofloxacin Induce Cell Cycle Arrest at G2/M Phase. *Bioorg. Med. Chem.* **2016**, *24*, 4636–4646. https://doi.org/10.1016/j.bmc.2016.07.070.
15. Chrzanowska, A.; Roszkowski, P.; Bielenica, A.; Olejarz, W.; Stępień, K.; Struga, M. Anticancer and Antimicrobial Effects of Novel Ciprofloxacin Fatty Acids Conjugates. *Eur. J. Med. Chem.* **2020**, *185*, 111810. https://doi.org/10.1016/j.ejmech.2019.111810.
16. Chen, R.; Zhang, H.; Ma, T.; Xue, H.; Miao, Z.; Chen, L.; Shi, X. Moxifloxacin/Gatifloxacin-1,2,3-triazole-isatin Hybrids with Hydrogen-Bond Donor and Their In Vitro Anticancer Activity. *J. Heterocycl. Chem.* **2019**, *56*, 2691–2694. https://doi.org/10.1002/jhet.3670.

17. Jiang, D.; Zhang, G. Ciprofloxacin/Gatifloxacin-1,2,3-triazole-isatin Hybrids and Their in Vitro Anticancer Activity. *J. Heterocycl. Chem.* **2019**, *56*, 2966–2969. https://doi.org/10.1002/jhet.3684.
18. Lancet, J.E.; Ravandi, F.; Ricklis, R.M.; Cripe, L.D.; Kantarjian, H.M.; Giles, F.J.; List, A.F.; Chen, T.; Allen, R.S.; Fox, J.A.; et al. A Phase Ib Study of Vosaroxin, an Anticancer Quinolone Derivative, in Patients with Relapsed or Refractory Acute Leukemia. *Leukemia* **2011**, *25*, 1808–1814. https://doi.org/10.1038/leu.2011.157.
19. Jamieson, G.C.; Fox, J.A.; Poi, M.; Strickland, S.A. Molecular and Pharmacologic Properties of the Anticancer Quinolone Derivative Vosaroxin: A New Therapeutic Agent for Acute Myeloid Leukemia. *Drugs* **2016**, *76*, 1245–1255. https://doi.org/10.1007/s40265-016-0614-z.
20. Hernández-López, H.; Sánchez-Miranda, G.; Araujo-Huitrado, J.G.; Granados-López, A.J.; López, J.A.; Leyva-Ramos, S.; Chacón-García, L. Synthesis of Hybrid Fluoroquinolone-Boron Complexes and Their Evaluation in Cervical Cancer Cell Lines. *J. Chem.* **2019**, *2019*, 5608652. https://doi.org/10.1155/2019/5608652.
21. Wang, X.; Jiang, X.; Sun, S.; Liu, Y. Synthesis and Biological Evaluation of Novel Quinolone Derivatives Dual Targeting Histone Deacetylase and Tubulin Polymerization as Antiproliferative Agents. *RSC Adv.* **2018**, *8*, 16494–16502. https://doi.org/10.1039/C8RA02578A.
22. Kumar, A.R.; Boddupally, V.L.; Rao, P.S.; Narsaiah, B.; Sriram, D.; Sowjanya, P. Synthesis and Biological Evaluation of Novel N1-Decyl and C7- Sec Amine Substituted Fluoroquinolones as Antitubercular and Anticancer Agents. *Indian. J. Chem. Sect. B* **2015**, *54*.
23. Rajulu, G.G.; Bhojya Naik, H.S.; Viswanadhan, A.; Thiruvengadam, J.; Rajesh, K.; Ganesh, S.; Jagadheshan, H.; Kesavan, P.K. New Hydroxamic Acid Derivatives of Fluoroquinolones: Synthesis and Evaluation of Antibacterial and Anticancer Properties. *Chem. Pharm. Bull.* **2014**, *62*, 168–175. https://doi.org/10.1248/cpb.c13-00797.
24. Abdel-Aziz, M.; Park, S.-E.; Abuo-Rahma, G.E.-D.A.A.; Sayed, M.A.; Kwon, Y. Novel N-4-Piperazinyl-Ciprofloxacin-Chalcone Hybrids: Synthesis, Physicochemical Properties, Anticancer and Topoisomerase I and II Inhibitory Activity. *Eur. J. Med. Chem.* **2013**, *69*, 427–438. https://doi.org/10.1016/j.ejmech.2013.08.040.
25. Saeed, B.B.; Al-Iraqi, M.A.; Abachi, F.T. Synthesis of New 1,2 Dithiol 3-Thione Fluoroquinolone Esters Possessing Anticancer Activity in-Vitro. *IJVS* **2012**, *26*, 115–121. https://doi.org/10.33899/ijvs.2012.168748.
26. Azéma, J.; Guidetti, B.; Korolyov, A.; Kiss, R.; Roques, C.; Constant, P.; Daffé, M.; Malet-Martino, M. Synthesis of Lipophilic Dimeric C-7/C-7-Linked Ciprofloxacin and C-6/C-6-Linked Levofloxacin Derivatives. Versatile in Vitro Biological Evaluations of Monomeric and Dimeric Fluoroquinolone Derivatives as Potential Antitumor, Antibacterial or Antimycobacterial Agents. *Eur. J. Med. Chem.* **2011**, *46*, 6025–6038. https://doi.org/10.1016/j.ejmech.2011.10.014.
27. Patitungkho, S.; Adsule, S.; Dandawate, P.; Padhye, S.; Ahmad, A.; Sarkar, F.H. Synthesis, Characterization and Anti-Tumor Activity of Moxifloxacin–Copper Complexes against Breast Cancer Cell Lines. *Bioorg. Med. Chem. Lett.* **2011**, *21*, 1802–1806. https://doi.org/10.1016/j.bmcl.2011.01.061.
28. Foroumadi, A.; Emami, S.; Rajabalian, S.; Badinloo, M.; Mohammadhosseini, N.; Shafiee, A. N-Substituted Piperazinyl Quinolones as Potential Cytotoxic Agents: Structure–Activity Relationships Study. *Biomed. Pharmacother.* **2009**, *63*, 216–220. https://doi.org/10.1016/j.biopha.2008.01.016.
29. Pardakhty, A.; Foroumadi, A.; Hashemi, M.; Rajabalian, S.; Heidari, M.R. In Vitro Cytotoxicity and Phototoxicity of N-Piperazinyl Quinolone Derivatives with a 2-Thienyl Group. *Toxicol. Vitr.* **2007**, *21*, 1031–1038. https://doi.org/10.1016/j.tiv.2007.03.011.
30. Rajabalian, S.; Foroumadi, A.; Emami, S. Functionalized N-(2-Oxyiminoethyl) Piperazinyl Quinolones as New Cytotoxic Agents. *J. Pharm. Pharm. Sci.* **2007**, *10*, 153–158.
31. Abdel-Aal, M.A.A.; Abdel-Aziz, S.A.; Shaykoon, M.Sh.A.; Abuo-Rahma, G.E.A. Towards Anticancer Fluoroquinolones: A Review Article. *Arch. Pharm. Chem. Life Sci* **2019**, *352*, 1800376, https://doi.org/10.1002/ardp.201800376.
32. Sharma, P.C.; Goyal, R.; Sharma, A.; Sharma, D.; Saini, N.; Rajak, H.; Sharma, S.; Thakur, V.K. Insights on Fluoroquinolones in Cancer Therapy: Chemistry and Recent Developments. *Mater. Today Chem.* **2020**, *17*, 100296. https://doi.org/10.1016/j.mtchem.2020.100296.
33. Samir, M.; Ramadan, M.; Hamed, M.; Osman, M.; Abou=Rahma, G. Recent Strategies in Design of Antitumor and Antibacterial Fluoroquinolones. *J. Adv. Biomed. Pharm. Sci.* **2021**, *4*, 134–151. https://doi.org/10.21608/jabps.2021.68305.1124.
34. Sissi, C.; Palumbo, M. The Quinolone Family: From Antibacterial to Anticancer Agents. *CMCACA* **2003**, *3*, 439–450. https://doi.org/10.2174/1568011033482279.
35. Yadav, V.; Talwar, P. Repositioning of Fluoroquinolones from Antibiotic to Anti-Cancer Agents: An Underestimated Truth. *Biomed. Pharmacother.* **2019**, *111*, 934–946. https://doi.org/10.1016/j.biopha.2018.12.119.
36. Swedan, H.K.; Kassab, A.E.; Gedawy, E.M.; Elmeligie, S.E. Design, Synthesis, and Biological Evaluation of Novel Ciprofloxacin Derivatives as Potential Anticancer Agents Targeting Topoisomerase II Enzyme. *J. Enzym. Inhib. Med. Chem.* **2023**, *38*, 118–137. https://doi.org/10.1080/14756366.2022.2136172.

37. Qashou, E.; Al-Hiari, Y.; Kasabri, V.; AlBashiti, R.; AlAlawi, S.; Telfah, A.; AlHadid, A. Antiproliferative Activities of Lipophililic Fluoroquinolones- Based Scaffold Against a Panel of Solid and Liquid Cancer Cell Lines. *Asian Pac. J. Cancer Prev.* **2022**, *23*, 1529–1537. https://doi.org/10.31557/APJCP.2022.23.5.1529.
38. Delaney, J.S. ESOL: Estimating Aqueous Solubility Directly from Molecular Structure. *J. Chem. Inf. Comput. Sci.* **2004**, *44*, 1000–1005. https://doi.org/10.1021/ci034243x.
39. Ali, J.; Camilleri, P.; Brown, M.B.; Hutt, A.J.; Kirton, S.B. Revisiting the General Solubility Equation: In Silico Prediction of Aqueous Solubility Incorporating the Effect of Topographical Polar Surface Area. *J. Chem. Inf. Model.* **2012**, *52*, 420–428. https://doi.org/10.1021/ci200387c.
40. Daina, A.; Michielin, O.; Zoete, V. SwissADME: A Free Web Tool to Evaluate Pharmacokinetics, Drug-Likeness and Medicinal Chemistry Friendliness of Small Molecules. *Sci. Rep.* **2017**, *7*, 42717. https://doi.org/10.1038/srep42717.
41. OECD Test Guidelines for Chemicals - OECD Available online: https://www.oecd.org/chemicalsafety/testing/oecdguidelinesforthetestingofchemicals.htm (accessed on 8 June 2024).
42. Croner-i. Harmonisation of Classification of Corrosives for Supply and Transport. Available online: https://app.croneri.co.uk/feature-articles/harmonisation-classification-corrosives-supply-and-transport?product=139 (accessed on 29 May 2024).
43. Barratt, M.D.; Dixit, M.B.; Jones, P.A. The Use of in Vitro Cytotoxicity Measurements in QSAR Methods for the Prediction of the Skin Corrosivity Potential of Acids. *Toxicol. Vitr.* **1996**, *10*, 283–290. https://doi.org/10.1016/0887-2333(96)00014-8.
44. Scott, L.; Eskes, C.; Hoffmann, S.; Adriaens, E.; Alepée, N.; Bufo, M.; Clothier, R.; Facchini, D.; Faller, C.; Guest, R. A Proposed Eye Irritation Testing Strategy to Reduce and Replace in Vivo Studies Using Bottom-Up and Top-Down Approaches. *ResearchGate* **2024**, *24*, 1–9. https://doi.org/10.1016/j.tiv.2009.05.019.
45. Prinsen, M.K.; Hendriksen, C.F.M.; Krul, C.A.M.; Woutersen, R.A. The Isolated Chicken Eye Test to Replace the Draize Test in Rabbits. *Regul. Toxicol. Pharmacol.* **2017**, *85*, 132–149. https://doi.org/10.1016/j.yrtph.2017.01.009.
